# Capacity Planning for Small Hospitals and Departments Illustrated Using Maternity and Paediatrics Departments: Roles for Weighted Population Density, Seasonality and Size, Myths Around Length of Stay and Factors Influencing Costs and Funding

**DOI:** 10.3390/ijerph23060711

**Published:** 2026-05-27

**Authors:** Rodney P. Jones

**Affiliations:** Healthcare Analysis and Forecasting, Wantage OX12 0NE, UK; hcaf_rod@yahoo.co.uk

**Keywords:** bed numbers, bed occupancy, forecasting demand, economy of scale, births, seasonality, Erlang equation, capacity planning, pregnancy and childbirth, maternity units, available beds, bed days per birth, queuing theory, hospital costs, medical deserts

## Abstract

**Highlights:**

**Public health relevance—How does this work relate to a public health issue?**

- Hospital capacity planning is important to meet population demand in a safe manner.
- Issues specific to small hospitals/departments are covered and two bed calculators are provided.

**Public health significance—Why is this work of significance to public health?**

- A role for weighted population density in the size distribution of hospitals/departments is identified and indicates that over 50% of world countries will have issues with sparsely populated ‘medical deserts’.
- Some countries have far smaller hospitals/departments than weighted population density would indicate, and this needlessly increases the average cost per patient.

**Public health implications—What are the key implications or messages for practitioners, policy makers and/or researchers in public health?**

- Small hospitals/departments require special funding arrangements with payment by HRG/DRGs not suited to their financial stability.
- The provision of healthcare to the population in ‘medical deserts’ requires national/federal intervention and hospital location must be optimized by ignoring artificial administrative boundaries.

**Abstract:**

The Erlang B equation is directly applicable to smaller hospital departments such as maternity and paediatrics departments. The bed occupancy margin is directly linked to size and not ‘efficiency’. A figure of 0.1% turn-away has been recommended as a planning target, i.e., only 1 in a thousand admissions suffer a delay before a bed can be found. Two bed calculators are provided which can be used for paediatric, obstetric, maternity, midwife-led, birthing wards and neonatal/paediatric critical care capacity. The negative effects of turn-away are likely to be context specific, hence, critical care > theatres > birthing unit > maternity unit. The uncertainty regarding future births is discussed along with the variable nature of seasonality in births. For paediatrics, much of bed demand is also influenced by the trend in births. Weighted population density (WPD) is associated with the size distribution of hospitals/units within countries and regions. This influences the average cost per birth/admission. The USA has a low WPD and a significant problem with small hospitals/departments. Only 10% of countries have WPD higher than England. Some countries choose to operate with even more hospitals than needed and this acts to elevate costs. Suggestions are made for a pragmatic approach to bed planning, especially where a dispersed population dictates a need for small hospitals, and hence, issues regarding size and costs. For maternity/paediatrics admissions (and other relatively short-stay admissions) the majority of overhead/indirect costs and most staffing costs should be apportioned based on admissions, and not LOS. Apportionment based on LOS creates the spurious illusion that LOS is the major cost driver and that reducing LOS will immediately save costs. Below 20 beds, Poisson statistical variation plus environment-induced randomness in daily arrivals imply that staff costs may become increasingly fixed irrespective of LOS. Around >30 beds, it looks possible to save costs by reducing LOS. Allocating total organizational costs to individual units and then to patients is less precise than realized and can be done in different ways, which all heavily rely on the steady-state assumption. When bed availability is the bottleneck, then reducing LOS may increase throughput per bed and increase income; however, is this for the benefit of the patient or for the benefit of the organization, and does it lead to higher unanticipated total costs including patient harm? The older economy-of-scale literature has been demonstrated to be flawed, with a recent focus on economy of scale at the department level being entirely consistent with the application of the Erlang B equation. A list of nine catastrophic pitfalls is given for doctors to identify dubious capacity advice from managers and external experts.

## 1. Introduction

### 1.1. Overview

This is part 4 in a series comparing international hospital bed numbers [1], bed demand and occupancy [2], and how to conduct ‘good’ hospital capacity planning [2,3,4]. This part is a linking study for both maternity [3] and paediatric capacity planning [4] due to their common reliance on trends in births as a primary factor in determining past and future bed capacity, and their relatively small size. They are used to illustrate issues common to many small hospital departments where the bed pool is reserved for a specific set of patients. The aim of the entire series is to present advice to capacity planners which is both pragmatic and academically sound.

To avoid self-citation a list of relevant publications covering 30 years of research is given in Supplementary Material S1. These references are grouped into alpha-numeric sections. They are referred to in the text as, see A.1 in Supplementary Material S1, etc. A separate series covering critical care is also available, see L.29, L.41, and L.55 in Supplementary Material S1.

Given that pregnancy and birth are a fundamental part of healthcare planning it is surprising that in England, over the last 35 years, no guidance has been given regarding how to calculate the required number of maternity/paediatric beds to support admissions for care during pregnancy and childbirth. How to make such calculations has seemingly been lost from the collective memory [3]. There is some guidance by the British Association of Perinatal Medicine regarding neonatal cot numbers and staffing [5], although the issue of determining cot numbers is somewhat vague. While a recent study outlined how to go about maternity capacity planning [3], a simple table linking annual births to the required number of available beds is lacking. Indeed, additional discussion regarding international trends in births is needed, as is a more detailed examination of the issue of variable seasonality (timing and magnitude) in births with more explicit advice on the best way to calculate ‘seasonality’.

Regarding a potential births-to-beds calculator, the basis for such a calculator lies in the field of Poisson statistics and queuing theory. Poisson randomness was presented in the paper by AK Erlang in 1909 and subsequently developed to include implications to queuing theory with the Erlang B and other queuing equations [6]. It was first used to size telephone exchanges and is now used with great confidence in multiple industries to calculate how many servers (beds, midwives) are required to meet incoming demand at various levels of ‘loss’, i.e., the customers/patients must wait or go away, which is called ‘turn-away’ in this study [6].

Using queuing theory and the Erlang B equation to calculate hospital bed capacity was first suggested in 1954 [7] and for obstetric units in 1959 [8]. It is now widely used in academic literature for all aspects of maternity and paediatric capacity planning [3,9,10,11,12,13,14,15,16,17,18,19,20], where it is especially relevant to unscheduled care [12,15]. Erlang B should therefore be widely used in hospital capacity planning, but widespread ignorance seems to prevail among hospital managers and government agencies. Indeed, almost every study on the adverse effects of high hospital bed occupancy has ignored the effect of size on turn-away.

This study demonstrates how such tables can be generated and is validated using maternity unit data from England and the USA. In many ways, that is the ‘simple’ part, because Erlang B assumes a steady state whereas births involve considerable seasonality. In addition, forecasting future births entails considerable complexity and uncertainty and this will be presented using examples from around the world.

Issues regarding whether reducing length of stay (LOS) in short-stay specialties such as maternity/paediatrics actually reduce costs are addressed in greater detail and it is proposed that most maternity/paediatric costs, both direct and indirect, should be apportioned on admission rather than LOS. Using LOS creates the illusion that LOS is ‘expensive’ and must be reduced to save money. An irrational focus on reducing LOS then ensues which may not benefit the patient or reduce total system costs.

Finally, the spatial distribution of population varies considerably between countries, and this dictates the size distribution of hospitals. Because size and costs are interrelated, the behaviour of costs is investigated and the thorny issue of how best to fund small hospitals/departments is discussed.

Before progressing further, the next section presents nine fatal errors in capacity planning. These are applicable to any capacity planning endeavour.

### 1.2. The Nine Fatal Errors in Capacity Planning

Over a 30-year career in hospital capacity planning, I have noticed that poor advice is often given by external ‘experts’ leading to the construction of hospitals or departments which are too small to be fit-for-purpose. This has led to the formulation of nine ‘never make these fatal errors’ in capacity planning.

1. Attempt to minimize capital and staff costs by devising the minimum case possible for all variables and assuming all schemes to reduce demand will simultaneously achieve 100% success. See point #8.
2. Use simplistic age-based forecasts for admissions based on a single year of data. Instead use more than 8 years of data (preferably 15 years), to follow the trend in each year of age. Then, take the trend into the future with multiple probable scenarios along with the observed (past) uncertainty associated with demand.
3. Calculate avLOS based on midnight stays. Always use real-time data because midnight LOS will consistently underestimate the real avLOS [3].
4. Assume that avLOS is a constant, rather than a variable with confidence intervals, and assume that avLOS decreases ad-infinitum. Most trends in LOS decrease toward an asymptote because there is a biological limit to recovery and/or the effect of medications. See K.1–K.9 in Supplementary Material S1.
5. Focus exclusively on those HRG/DRGs which show above-average LOS. These will generally be matched by other HRG/DRGs with lower-than-average LOS. These arise due to the ambiguities in the local clinical coding process compared to that applying to the national average. This includes how doctors record diagnoses and the depth of local coding with complications and existing conditions affecting health. Local LOS is subject to sampling error as it is a small subset of national data [21].
6. Use annual averages for admissions and avLOS. Many conditions show seasonality due to multiple causes and LOS can also show seasonal variation.
7. Assume that lower avLOS means better care or that lower avLOS results in large savings in costs. It is the volatility in admissions which often dominates bed demand not the calculated avLOS—this directly contradicts the accepted dogma that reduction in LOS is one of the key ingredients to reducing bed demand. Reducing LOS only benefits a steady-state system (such as elective/scheduled care) or the baseline bed demand which lies beneath the volatile changes in emergency/unscheduled care, see L.6 and L.9 in Supplementary Material S1.
8. Assume that 85% occupancy is a proven figure. Proof of 85% as an optimum occupancy does not exist and most categorically does not apply to small bed pools, see references and discussion in [1,2,3].
9. Make simplistic models comprising all of the variables and proposed schemes to reduce admissions and LOS. An alternative is to use Monte Carlo simulation (including seasonality), which will show the full range of probable outcomes. This is a subset of operational research [22,23,24]. The alternative is to use past data to illustrate the sources of variability—upon which Monte Carlo simulation will be based but without the full nuances of the real world. Hence, simultaneous variation in admissions and LOS implies that the actual trend in occupied bed days is a preferred approach.

The nine fatal errors have been regularly observed by the author relating to capacity planning in England [1,2,3]. These were forced on the English NHS in an environment where politicians had an erroneous belief that the NHS had too many beds [2]. This was in addition to a serious policy fiasco where the Private Finance Initiative (PFI) for building hospital capacity necessitated Treasury rules for the affordability of PFI projects, where the fiscal rules contradicted the real world of how bed demand behaved [2].

These nine fatal errors apply across all aspects of hospital bed planning and are illustrated in both the maternity [3] and paediatric [4] capacity planning studies.

Because data and relevant studies are readily available for maternity and paediatrics, and both are relatively small departments, they are used to illustrate many of the concepts in this study.

## 2. Materials and Methods

### 2.1. Sources of Data

Data relating to births and available beds for US maternity units in 2000, 2009 and 2019 were from the study of DeSisto et al. [25], while data on English maternity units were from NHS maternity statistics for births in 2023/24 [26], and data on available beds in 2023/24 were from the NHS England bed availability KH03 series [27]. Neonatal unit data are from winter daily SITREPS [28].

Births by hospital site in various countries were from Northern Ireland [29,30], Wales [31], Scotland [32], New Zealand [33,34], Sweden [35], Switzerland [36], and Belgium [37,38,39].

International births were from the United Nations [40], annual births in the USA were from [41], births in Arizona were from [42] and births in Hawaii were from [43]. Monthly births in European countries were from Eurostat [44], and annual births in Australian regions were from [45].

The number of available maternity/midwifery beds at New Zealand public hospitals was from [34].

Monthly deaths by age and sex, in England and Wales, were from [46]. Data relating to vaginal delivery and caesarean section (C-section) in Australian hospitals were from [47].

The population of England, by age band since 2012, was obtained from [48], and hospital admissions by specialty or by ICD-10 diagnosis were obtained from [49]. Population density in lower super-output areas, in England and Wales, was obtained from [50], population density in Scottish output areas was obtained from [51], population in US states and counties, 2024, was obtained from [52], and the surface area of US states and counties was obtained from [53]. Raw population density in US counties was obtained from [54]. Weighted population density (WPD) in US states and the largest cities was obtained from [55,56]. Deaths in US states and counties were from [57], and births in US states from 2007 to 2010 were from [58] and from 2011 to 2014 were from [59]. Age standardized mortality rate in US states was obtained from [60].

Magnetic resonance imaging (MRI) scanners and scans in the UK [61], MRI scanners in the USA [62], and scans in the USA [63] were obtained. Weighted population density (WPD) in world countries was obtained from [64].

### 2.2. Additional Data from English Hospitals

Additional data covering births in 2023/24 split by site were obtained by Freedom of Information requests to larger English NHS Trusts.

### 2.3. Manipulation of Data

#### 2.3.1. Estimating Births in Australian and Welsh Hospitals

Data regarding the number of admissions at each Australian hospital for vaginal delivery and caesarean section [47] were added together and this total was then uplifted to total births in 2023 [45]. Hospitals were then grouped in 1000 increments in births up to >7000.

For Northern Ireland, data for admissions in the women’s and children’s group [29] were taken for each hospital and factored down to match with total births in 2024 [30].

#### 2.3.2. Births in US Hospitals by 1000 Birth Increments

Data from DeSisto et al. [25] cover only 7 broad intervals up to 7000+. The first 3 bands up to 999 births were added together. Above 1000, each broad band was approximately split, i.e., band 4000 to 6999 was split into 5 bands to give a smooth transition between the adjacent bands, namely, 2000 to 3999 and 7000+. High precision is not required.

#### 2.3.3. Births in US Hospitals and the Ratio of Births per Bed

Data from DeSisto et al. [25] cover 7 broad intervals up to 7000+. For the purpose of plotting births versus available beds per 1000 births data on a chart, births were taken as a single value by estimating that the average was reasonably close to the maximum value in each band. High precision is not required.

#### 2.3.4. Linking Births and Beds for English Maternity Sites

For each NHS Trust, annual births and beds were linked in an Excel spreadsheet. For the larger NHS Trusts, a Freedom of Information request was sent to obtain the number of births at each site run by the Trust.

#### 2.3.5. The Occupancies at 0.1%, 1% and 3% Turn-Away

Data from the Erlang equation at 0.1%, 1% and 3% turn-away were taken from Jones [3,4], where occupied bed days were converted to births by assuming that each birth was associated with 3.2 occupied bed days per birth. This is the total of all bed days (in real time) associated with admissions during pregnancy and during childbirth, but excluding time spent in the birthing unit. The tabular data of Jones [3,4] goes in 1 bed increments up to around 50 beds and wider increments (usually 5 beds) were converted to 1 bed increments by linear interpolation.

#### 2.3.6. Cost Weights Applied to the Proportion of Births in Each 1000 Increment of Births

The band > 7000 births per hospital was the reference point with a weight of 1.0; then, in decreasing order, the bands were 6–6999 births (+3%), 5–5999 (+6%), 4–4999 (+9%), 3–3999 (+15%), 2–2999 (+25%), 1–1999 (+42%) and <1000 (+220%). Due to the unusually high proportion of very small maternity units in the USA, additional weights were derived for <250 births (+805%), 250–499 (+380%), and 500–999 (+295%).

#### 2.3.7. Adjusting Weighted Population Density (WPD) for Different Calculation Methods

The method with the highest resolution is that of Edwards et al. [64] and uses 1 km grid squares. The method used in the USA is based on census tracts [55,56], which contain approximately 4000 people and have an average size of 112 km^2^. Due to the peculiarities of US population distribution, multiply these figures by 1.015-times to approximate the method of Edwards et al. [64]. The method in England, Wales and Scotland [50,51] is based on lower super-output areas (LSOA) which contain an average of 1700 people and a size of 4.8 km^2^. Multiply by 1.05-times to approximate Edwards et al. [64].

## 3. Results

### 3.1. Defining a Bed Pool and Real-Time Length of Stay

A bed pool is a collection of beds dedicated to a set purpose such as adult/child, maternity/other care, the critical care unit, the birthing unit, etc. Other types of care are not allowed to cross into the defined bed pool, and most importantly, the bed pool size must be determined at each site if the organization operates across multiple sites.

The bed pool then becomes the fundamental unit for capacity planning. The planner must therefore understand which patients use the different bed pools. In most instances this can be achieved using the ward name. Some discretion is also allowed if patients can be quickly transferred between sites to balance demand among the smaller bed pools. These become intra-organization transfers as opposed to officially recognized inter-organization transfers.

Regarding small specialties, in the English NHS, there are around 80 consultant (of care) specialty and sub-specialty codes [49]. Half of these will have the need for <3 beds per hospital and hence, their patients will be accommodated within a larger bed pool. In addition, all of the paediatric sub-specialties will generally only occur in larger children’s hospitals. In general, a hospital with <30 beds will have fluid boundaries, see L.12 in Supplementary Material S1. In small community/rural hospitals, out of necessity, the hospital itself has become a multi-specialty bed pool, usually via single rooms.

All hospital patient management systems document admission and discharge as day:hour:minute. Midnight-based length of stay (LOS) simply counts the number of nights that the patient was in hospital and gives an integer value. Real-time LOS therefore counts the difference between admission and discharge as days, hours and minutes, which is then converted into a decimal number. Same-day-stay admissions will therefore have a decimal value less than one for LOS. The average LOS (avLOS) is therefore calculated from the entire set of decimal values. The difference between midnight and real-time LOS has been noted to have a significant impact on the evaluation of real bed demand in both critical care units [65] and maternity units [3].

### 3.2. Maternity and Pediatric Units at Different Hospitals Have Different Sizes

**Figure 1:**
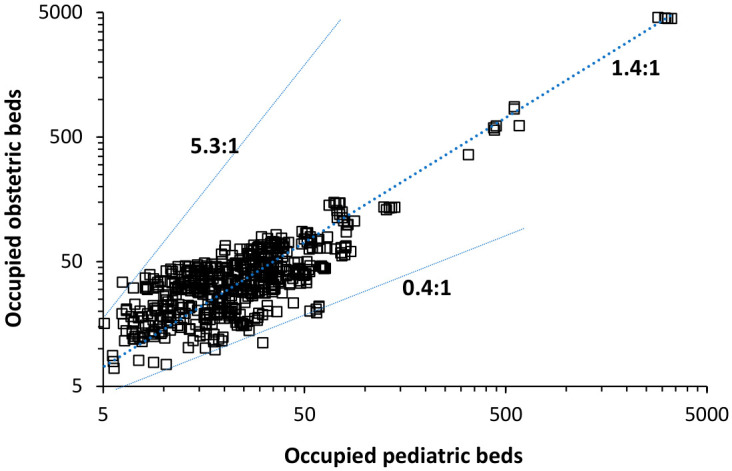
Occupied paediatric and maternity beds (at midnight) in English hospitals and English regions. Quarterly data (March 2025 to December 2025), then regional and England at the far right [27].

This study forms a common overlap between maternity and paediatric capacity planning and so it is useful to note that when located at the same hospital, they can have very different sizes; hence, they are examples of variation between any two small departments/units at different hospitals. Data for English hospitals in Figure 1 cover four consecutive quarters in 2025 [27] and are restricted to hospitals that have both a maternity and paediatric unit.

Figure 1 excludes around 11% of acute hospitals such as children’s hospitals without a maternity unit such as Great Ormond Street and vice versa. It is based on midnight occupancy and so excludes any LOS associated with same-day-stay admissions. Figure 1 displays the data for all English hospitals meeting the criteria, then data for English regions where the ratio will also include free standing women’s and paediatric hospitals and finally, data for the whole of England.

On the left-hand side of Figure 1 are quarterly and annual average data for individual hospitals, and then quarterly and annual average data covering English regions and finally, on the right, the ratio for the whole of England. Figure 1 shows that there is wide variation in the ratio of obstetric to paediatric occupied beds from 0.4:1 up to 5.3:1 at different hospitals, 1.1 to 1.6 at the regional level and 1.4 (range 1.3 to 1.6) for England. The flow of patients to each depends on the ring of surrounding hospitals, and their reputation, accessibility and capability.

At the hospital level, the standard deviation associated with bed occupancy is higher for paediatrics, being 1.4-times higher at the largest site rising to 2-times higher at the smaller sites. Maternity seasonality is mainly restricted to higher births around September [3] but is far more complex for paediatrics [4]. In addition, the scatter increases as the size of the unit decreases, which is an expected outcome from Poisson-based randomness in arrivals and case-mix variation for units serving different populations. In addition, view this scatter in the light of financial risk arising from income instability.

Figure 1 also illustrates the important point that all capacity planning should be conducted using real-time length of stay (LOS) and not midnight occupancy [3]. In terms of same-day-stay admissions in England, the specialty paediatrics only has 36% of total admissions that are same day, while obstetrics has 41% and midwifery has 46% [47]. Hence, when looking at acute hospital totals across England (excluding mental health) over the years 1998/99 to 2023/24, where occupied bed days includes a contribution from same-day admissions, it is observed that occupied beds for the specialty paediatrics (specialty code 420) account for 5.6% to 6.8% (range over 17 years) of acute occupied beds, while maternity only accounts for 3.5% to 4.5% of the total. This data source will attribute LOS in over 10 large children’s hospitals (often on the same site as a multi-specialty hospital) to the paediatric total. Regarding available maternity beds, in Belgium, available maternity beds account for an average of 6.8% of total hospital beds [37] compared to 7.5% in England [27], which excludes mental health.

The ratio between paediatrics and maternity will vary between countries, especially if the average LOS is markedly different than that in England [66], and if real-time LOS is used [3]. The scatter at the regional level will also occur because of the unequal distribution between free-standing children’s and women’s hospitals, which will distort flows to nearby surrounding general paediatric and maternity units.

In conclusion, whatever the exact ratio at each hospital, both specialties are small in relation to total acute care. Due to the relationship between size and bed occupancy (see later) it is important to use real-time occupied bed days rather than available beds. The main point is that similar sizes imply approximately the same issues with economy of scale and the volatility in income. In addition, a small paediatric/maternity unit will usually imply that they are located in a small hospital.

### 3.3. How Many Beds Are Needed for Various Levels of Births or Admissions?

As presented in previous studies [2,3], see also L.1–L.58 in Supplementary Material S1, the Erlang B equation can be used to link available beds, bed occupancy and turn-away. Turn-away measures the proportion of time that a bed is not immediately available for the next patient and is therefore a measure of delays to treatment, hidden queues, cancelled operations, transfers to other hospitals and a chaotic operational situation. For scheduled care, the turn-away is reflected in the delay between the decision to intervene and the actual intervention.

All Erlang calculators exist to help make management decisions by performing alternate calculations under various assumptions. The output must always be compared against real data. Avoid annual averages to size a unit as the idea is to estimate peak demand.

To assist hospital managers, two bed calculators will now be discussed and are available in Supplementary Material S2, namely, the following:

1. A modified Erlang B calculator using births and total bed days per birth, which calculates required beds at an assumed 0.1%, 1% and 3% turn-away.
2. An Erlang B calculator using daily admissions and average length of stay, which shows average occupancy and turn-away for 1 bed increments in available beds.
3. A sheet showing the base data.

Given that all care during pregnancy and childbirth requires immediate attention, a turn-away of 0.1% or less is recommended. Strictly speaking, the admission rate and length of stay associated with the peak month for births should be used since an annual average will underestimate real bed requirements. This is covered in the section regarding seasonality. If the unit is currently operating at high turn-away, then the total bed days per birth will be lower than the required level and will need an uplift. One solution is to estimate the bed days per birth during the months when births are lowest and turn-away will also be lower.

Erlang depends on accurate length of stay (LOS), which must be derived from real-time data. Average LOS based on midnight stays give averages which are lower than reality [3]. In addition, an allowance must be made for the time it takes to get the bed ready for the next patient. There is no point in fooling yourself by feeding a low LOS figure into the model.

#### 3.3.1. A Births-to-Beds Calculator

The first calculator is a births-to-beds calculator, which uses outcomes from Erlang B that meet the criteria of 0.1% turn-away; however, it uses the expected number of annual births (a readily available statistic) and the ratio of total bed days per birth. This is found in the Read Me 1 section of the spreadsheet in Supplementary Material S2. A calculator at 1% and 3% turn-away is also given, and the base data are provided in another tab. The reason for the 1% and 3% turn-away calculators is covered later.

For the maternity unit, total bed days include all stays during pregnancy and after discharge from the birthing unit. For the birthing unit, this will simply be the average stay in the birthing unit. For the neonatal unit, the total bed days will be directly from that unit divided by the total births. Hence, there is a hidden conversion for the proportion of births that are admitted to neonatal critical care. The same reasoning applies to any ambulatory ‘assessment’ units whose beds and occupancy must be calculated separately. This calculator asks the user to input the value for seasonality in cell A2. This is a locally derived figure or nearest estimate from a later section dealing with seasonality.

This calculator also shows the ratio of required beds per 1000 births, which is a measure of bed throughput. It immediately reveals that smaller units rapidly lose economy of scale and incur inescapable and escalating costs per birth. Table 1 illustrates the output from this calculator at three different levels of total bed days per birth. In this example, no adjustment has been made for seasonality. For each increment in available beds, the annual births column shows the maximum births consistent with 0.1% turn-away. The next column shows this as a ratio of beds per 1000 births. The ratio of beds per 1000 births demonstrates that diseconomy of scale is operating powerfully below 10 beds.

As can be seen in Table 1, a 6-bed unit can only accommodate 131, 105 or 84 births at 3.2, 4 or 5 total bed days per birth. Such small units are usually not resourced for complex patients and/or will not have neonatal capacity and so mother/baby will be rapidly transferred to a larger unit. Hence, their effective bed days per birth will be lower than the 3 bed days per birth in the first column. Note that maternity length of stay varies considerably between countries [66].

The calculator also contains provision to input variables relevant to the birthing unit such as day-of-week (cell A3) and hour-of-day (cell A4). The study of Dexter and Macario [14] describes the day-of-week and hour-of-day factors relevant to birthing units.

Should you wish to check the overall size of an Obstetric or midwife-led unit using this calculator, then use the same number of annual births but the total bed days per birth will be the sum of all LOS (in real time) across all types of beds such as antenatal/postnatal, birth centre, labour ward, triage or recovery beds. This will give a total unit size which is slightly too small due to the economy of scale implied by the larger total of beds. As a rough guide, total beds should be around 1.3- to 1.4-times the number of maternity beds. The higher figure is likely for an obstetric unit while the lower figure is likely for a mixed obstetric/midwifery unit.

Also included in Supplementary Material S2 are sheets covering 1% and 3% turn-away. These sheets are included to cover situations where there may be one or more competitor units in the same town (common in the USA), and when mothers can be diverted to an alternate unit. Some insurers in the USA restrict access to a specific unit and on this occasion, 0.1% turn-away should be used.

The alternative use of 1% or 3% turn-away is for small midwife-run units surrounding a central obstetric unit (as is common in England). At points when the smaller midwife unit is full, the mothers can be diverted to the larger obstetric unit. The choice of 1% or 3% turn-away will depend on the size of the midwife unit.

#### 3.3.2. Validating the Births-to-Beds Calculator

Erlang B has been used with great confidence over many years for emergency/unscheduled care such as maternity care [3,7,8,9,10,11,12,13,14,15,16,17,18,19,20] and the study of De Bruin et al. [15] specifically noted that Erlang B is an excellent match with maternity care. However, it is nevertheless useful to validate the outputs against various real-world situations.

##### Maternity Occupancy in USA Versus England

**Figure 2:**
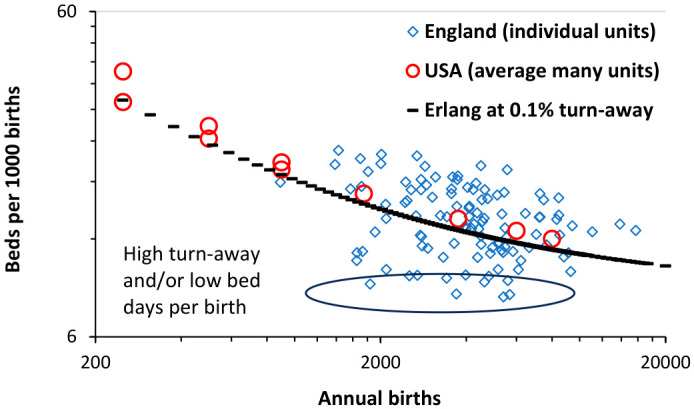
Ratio of available beds per 1000 births in the USA and England with an assumed 3.0 bed days per birth using Erlang B at 0.1% turn-away. Data from [25,26,27].

The first check against the real world is Figure 2, which shows the relationship between available beds per 1000 births and births for two data sets, namely individual maternity units in England [26,27] and national averages for units of different sizes in the USA [25]. Data for the USA are the highest and lowest values observed over the years 2000 to 2019. Data for England have been trimmed to exclude implausibly high/low values. Regarding implausibly low figures for bed occupancy, I have contacted a number of Trusts and have discovered that some are incorrectly reporting the data, with different error sources.

Figure 2 uses the ratio of beds per 1000 births to illustrate the effect of size on bed utilization as shown in Table 1. As can be seen, both the US and English data lie along the line described by the Erlang equation at 0.1% turn-away and an assumed 3 bed days per birth. England will have slightly higher LOS than the USA; hence, the data lie slightly above that for the USA.

There is some scatter in the English data simply because around half of English units operate above 0.1% turn-away [3].

For context, the smallest general maternity unit in England has around 10 beds because England has far higher population density than the USA. England and the USA tend to operate at very low length of stay compared to other countries [66]. By way of comparison, there are around 23.3 to 25.9 beds per 1000 births in Belgium where 37% of maternity units are in the range 500–1000 births per annum and the three largest units have just over 3000 births p.a. [37,38,39]. The two largest maternity units in New Zealand (births in brackets) operate at 11.2 beds per 1000 births (6060) and 10.4 (7240), respectively [33,34]. Both lie close to the trend line in Figure 2.

##### The 2012–2013 Maternity Capacity Shock in England

Due to a long-term cycle in births arising from the World War II baby boom, England experienced a cyclic maximum in births at the 12-month period ending October 2012. This was 24% higher than the cyclic minimum reached at the 12 months ending in June 2002 [3]. No planning guidance was ever issued regarding this impending cyclic maximum [3].

The impact of this failure to plan was documented in a 2013 National Audit Office (NAO) [67] report conducted during the 2012 part of the peak in births which showed that 12% of maternity units were capping entrance to pregnant mothers, while in the period from April to September 2012, some 28% were closed to admission for more than 12 h, with an average closure of 3 days. Some 11% were closed to admission for >14 days [67]. Another study published in 2011 [68], around the time of this peak, identified variation in staffing levels, vacancy rates and staff turn-over. Considerable variation between units regarding throughput per bed indicates issues with available capacity and turn-away. Hence, this represents an independently validated capacity crisis.

Data for available and occupied maternity beds in England for each of 140 NHS Trusts with maternity services for the quarter ending in September for the 2011/12, 2012/13, and 2013/14 financial years were extracted from the KH03 data collection [27]. The data were compared against the 0.1% turn-away line, and the size of the bed deficiency/excess was quantified. This is shown in Supplementary Material S3. Instances of a bed deficit are highlighted with a red background while surplus beds are highlighted with a green background. The chart in S3 shows all units relative to the 0.1% turn-away line. The September quarter was chosen because this represents the seasonal maximum in births [3]. Some 57% of units had at least one occurrence above 0.1% turn-away.

Maternity units near to 100% occupancy correspond to those capping admissions or closed to admissions for extended periods of time as in the NAO report [67]. Unfortunately, the NAO was unaware of the importance of Erlang B in identifying such problems.

The surprising thing to note is that while some hospitals had clear bed deficiencies, others had excess beds to which patients were probably diverted from those which were limiting admissions [67]. Clearly there was no consistency in maternity bed capacity across England [3]. One problem here is that the Department of Health had devolved all capacity planning to individual hospitals but had not provided any guidance as to what good maternity capacity planning may look like [3].

The final point is the use of Erlang B as a data quality check. Note the cluster of units lying below 20% average occupancy in the tab ‘Chart compared to turn-away’ in Supplementary Material S3. My own investigation reveals that some units are incorrectly reporting occupancy data. In England, the definition of a maternity bed is for antenatal + postnatal (AN+PN) care and specifically excludes beds in the birthing unit. Some units appear to count birthing beds in the available bed total but correctly count occupied AN+PN beds. In addition, note instances where the reported beds occupied for a particular unit will be unusually low compared to the other quarterly figures. On these occasions, other data errors look likely.

In summary, Erland B confirms the capacity shock identified by others [67,68], exposes huge variations in bed availability and staffing identified in other reports [68], and quickly identifies possible instances of erroneous data reporting. It is eminently applicable to the real world.

##### Daily Measurement of 100% Occupancy

**Figure A1:**
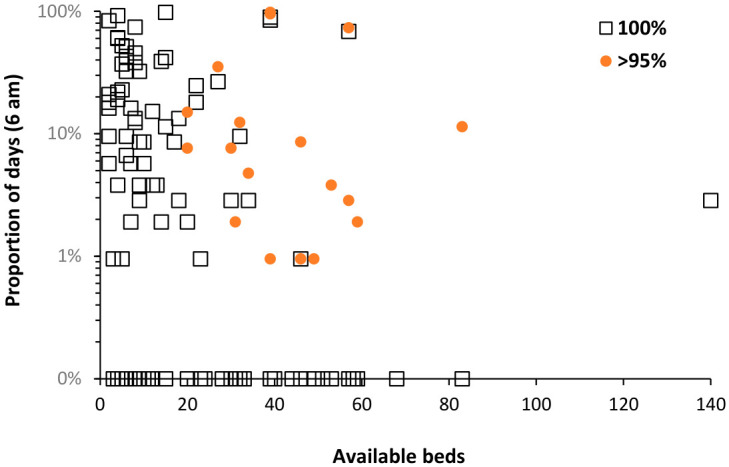
Proportion of days that English neonatal intensive care units (NICUs) are 100% or 95% occupied at 6am. Data are from a 105-day period covering 24 November 25 to 8 March 26 [28]. This period is not at the seasonal peak for births. The data at 140 beds covers multiple sites and should be moved to the left to reflect a smaller per-site average size. NICU are mainly level 1 (approx. 75%) but also contain level 2 and 3 units.

There is a final way to validate the births calculator which is independent of the Erlang equation. This approach relies on daily measurements of percentage occupancy at a fixed time each day. Since 100% occupancy is equivalent to 100% turn-away, the proportion of time that the unit is at 100% occupancy is measured. This is illustrated in Figure A1 in Appendix A using data from English neonatal intensive care units where occupancy is measured at 6 am [28]. Proportion of days with >95% occupancy was also measured as it is an indicator of a high risk of 100% occupancy at times other than 6 am.

The data are for NHS Trusts, which may have more than one maternity unit in the same district; hence, bed numbers and occupancy are aggregated and will therefore underestimate the real proportion of days at 100% occupancy at the individual sites. However, with this limitation aside, it is clear that around 40% of units do not experience 100% occupancy and therefore would be deemed to meet the 0.1% turn-away criteria. For the other units, those with fewer than 20 NICU beds tend to experience higher proportions of days at 100% occupancy. Hence, 10% of days at 100% occupancy is approximately the same as 10% turn-away, etc. These units require more available NICU beds, although with periods of one nurse covering two babies. The approach is very different but gives the same answers as those from Erlang B. This method of presenting the data is probably more easily understood by the public and avoids complex terminology and charts with lines of turn-away.

##### Avoiding Misplaced Attempts to Impose Occupancy Targets

All will be aware of the 85% occupancy target for hospitals. Many will be unaware that this is the equivalent to an often repeated ‘urban myth’ which has never been proven [2,3]. Hence, Erlang B provides a genuine basis for misplaced but well-intended attempts to ‘improve’ maternity bed occupancy. In Belgium, one such universal occupancy rate for maternity units was imposed but thankfully was ignored by all hospitals [37].

#### 3.3.3. A Calculator Using Erlang B for Other Departments

The second calculator uses the Erlang B equation [12] where the daily admission rate and average length of stay are entered. In Supplementary Material S2, this calculator is found in the Read Me 2 section.

To those unfamiliar with Erlang B, the equation has two parts, namely, a top part which is divided by the bottom part [12]. Erlang B involves the manipulation of very large numbers which even computers struggle to handle; however, the calculator will handle almost all scenarios applicable to the majority of units, especially below 30 beds. Above 30 beds, multiple Erlang B calculators can be located by an internet search. These use a variety of estimation methods to handle the very large divisions required by Erlang B.

The sheet ‘Turn-away and occupancy’ is where the daily admission rate and avLOS are input and the user simply scrolls along row 4 to find the number of beds compatible with the desired level of turn-away. Row 6 gives the average occupancy at this level of turn-away while row 8 shows the average number of occupied beds. This calculator can be used for all types of bed pools including the maternity ward, the birthing unit, neonatal and other critical care, paediatric units, a cardiac ward, etc.

The excellent study of Dexter & Macario [14] gives an example of how to use this calculator when there are day-of-week and hour-of-day patterns in the arrival rate and bed occupancy. In addition, note that day-of-week factors will be required to cope with the impact of ‘elective’ maternity (such as C-sections, etc.) admissions. In parturition, there is some discretion to move otherwise unscheduled events to scheduled intervention. Hence, the arrival rate will have to be adjusted upward to account for the general Monday to Friday workload from any ‘elective’ events.

In the case of small community/rural hospitals where pregnancy and childbirth are part of a wider mix of services, then it is appropriate to use the (all-specialty) admission rate to the entire hospital and the resulting whole-hospital avLOS as the inputs. Use historic data to scan for seasonal profiles and if bed demand varies by the day of the week and hour of the day. Adjust the inputs into the calculator appropriately. It is assumed that maternity/birthing care can occur in any appropriate hospital bed and even in the ED.

Finally, do not assume that avLOS is a constant and check for any seasonal variation and associated subtle changes in case mix.

#### 3.3.4. Comparing Differently Sized Neonatal Critical Care Units

Previous studies have used Erlang B loss tables to show multiple lines of constant turn-away where the unit size and average occupancy is input, thereby allowing a like-for-like comparison between multiple units of disparate sizes [2,3,4], see also L.2–L.6, L.9, L.12, L.17–L.22, L.29, L.30, and L.44 in Supplementary Material S1.

**Figure 3:**
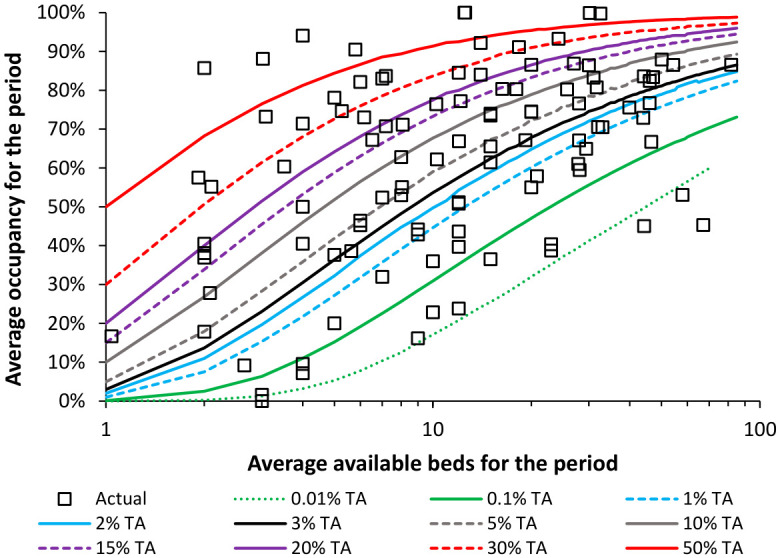
Average bed occupancy and available beds in English neonatal intensive care units (NICUs) for the autumn/winter/spring period in 2025/26, along with turn-away lines from Erlang B. Data from [28]. TA = turn-away.

Figure 3 shows the situation for neonatal intensive care units (NICUs) in England for the autumn/winter/spring period in 2024/25. Figure 3 takes the lines of turn-away from Supplementary File S2 and plots the average of the daily occupancies at 6 am over the 127-day period from 25 November 2024 to 30 March 2025 [28]. The *X*-axis is a logarithmic scale. Note that Figure 3 is the equivalent to Figure A1 in Appendix A, which uses the daily measurement of instances of 100% occupancy. Occupancy and turn-away for these units in the winter of 2023/24 have been previously reported [2,3]. Some 19 units achieve 0.1% turn-away or lower (down to 0.01% turn-away) and a further 14 units achieve between 0.1% turn-away and 1% turn-away. A surprising number of units operate above 20% turn-away (equivalent to 100% occupancy occurring on 1 in 5 days), and 7 units operate above 50% turn-away (based on reported data), which has not been investigated in terms of adverse outcomes. This type of chart is recommended for regulatory agencies with units falling higher up on the chart relative to the lines of turn-away needing to be investigated to see if this is associated with higher mortality or other adverse outcomes.

One limitation of Figure 3 is that the data are from types I, II, and III NICUs and ideally, each type should be plotted on a separate chart. Level I care implies stabilization and transfer to a more appropriate unit and may therefore be expected to operate at higher turn-away. NICU is characterized by high levels of immediate access but with only around 20 units functioning near to or below the 0.1% turn-away line. English units tend to operate in a hub and spoke arrangement centred around larger regional hospitals (lying at the right-hand side of Figure 3); however, units above the 20% turn-away line are likely to need more beds. For both PICUs and NICUs in England [2,4], several units operate above 85% average occupancy and will therefore experience the combined deleterious effect of high turn-away and high busyness [2]. Compared to the USA, England has fewer NICUs per 1000 births and this is reflected in higher overall turn-away in Figure 3 [4].

Regarding the level of safety for maternity units, note in Figure 3 that around 20 units consistently function above 85% average occupancy. Based on the principle of the link between busyness and patient safety [2,3], such units should be on the hospital’s risk register and questions should be raised as to why this situation has not been addressed. Recall that higher turn-away is a risk factor but that the expression of this risk depends on the level of staff busyness (which is sometimes approximated by the 85% occupancy figure) [2,3]. Busyness should be directly measured by staff to patient ratios, not occupancy.

### 3.4. The Size of Maternity Units in Different Countries

**Figure 4:**
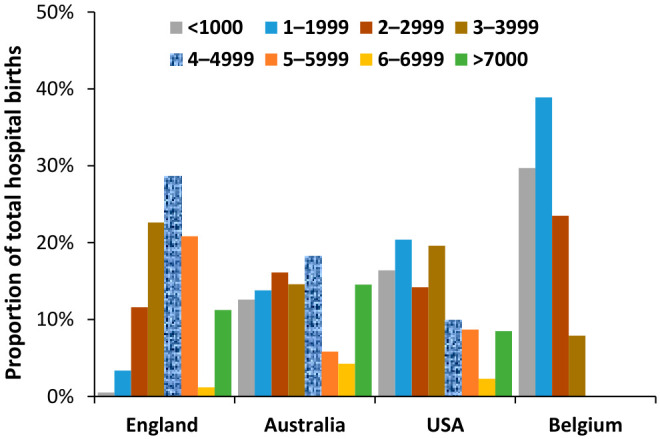
Proportion of total maternity unit births by unit size (births as 1000 births bands) in Australia, Belgium, England and the USA as number of annual births. Data from [25,26,37,38,39,45,47].

While the USA has several large cities, only two states have more than 1000 persons per square mile (equal to 390 persons per square kilometre) [52,53,54]. Over 90% of the surface area encompasses low-density rural areas [52,53,54]. This implies a disposition to small maternity and paediatric units commencing with <5 beds [25,69,70]. In contrast, England has very high population density and this is reflected in almost all maternity units having above 10 beds (1000 births). Australia has another geographical distribution with large cities along the coastline. Prior to 2019, Belgium had a system focussed on numerous small units [37,38,39]. These issues are illustrated in Figure 4, where the distribution of maternity units by size in Australia is somewhat like the USA due to a disparate rural population. Belgium had a disproportionately high proportion in the bands <1000 births, and the band 1–1999 births, especially outside of Brussels—although this is intended to undergo rationalization once legislation is passed [37,38,39].

The data for England have been broken down to the level of single sites, while those for the USA may include some organisations operating from multiple sites, especially in the >7000 births category.

### 3.5. Population Dispersion and Healthcare Costs

**Figure A2:**
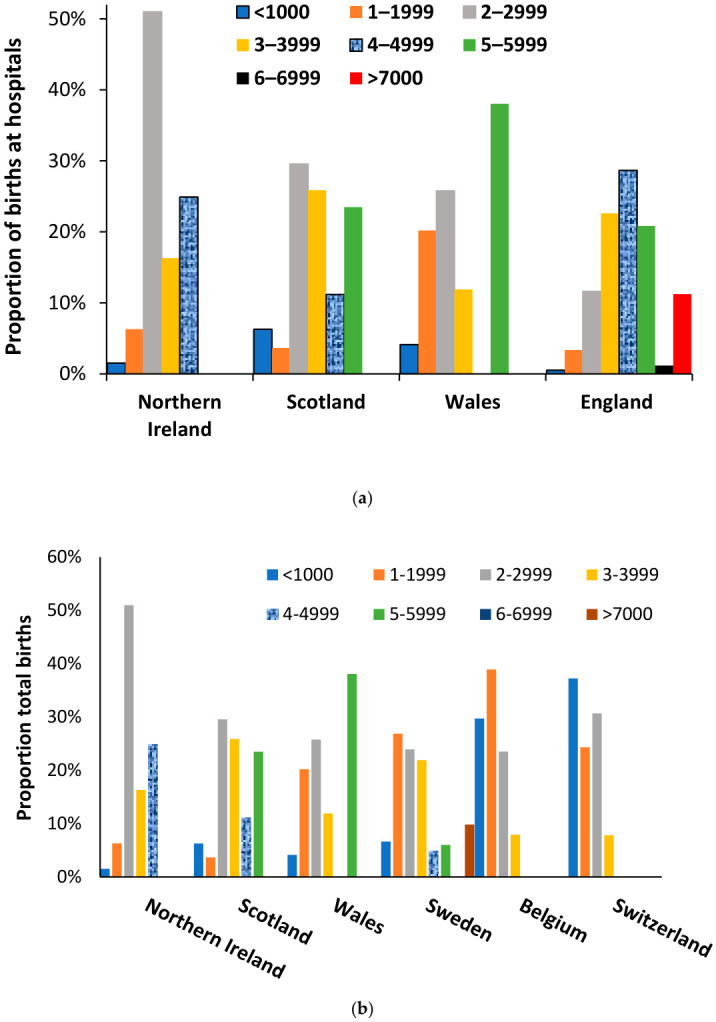

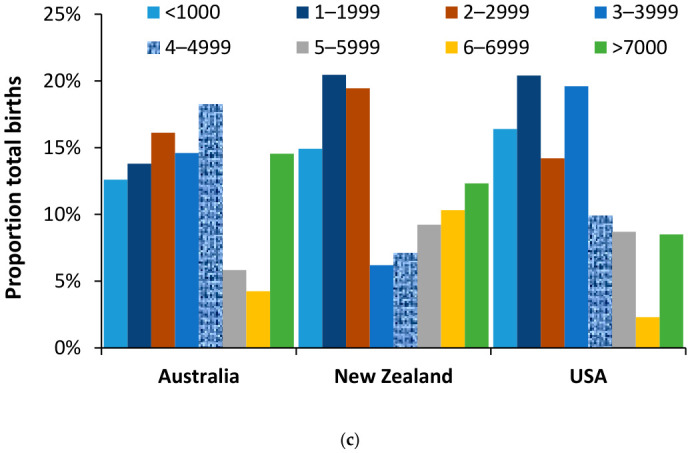
Distribution of number of births in different countries. (**a**) The four countries of the UK [26,30,31,32], (**b**) countries with smaller maternity units [29,30,31,32,35,36,37,38], and (**c**) countries with highly dispersed populations [25,33,34,44,46].

**Figure A3:**
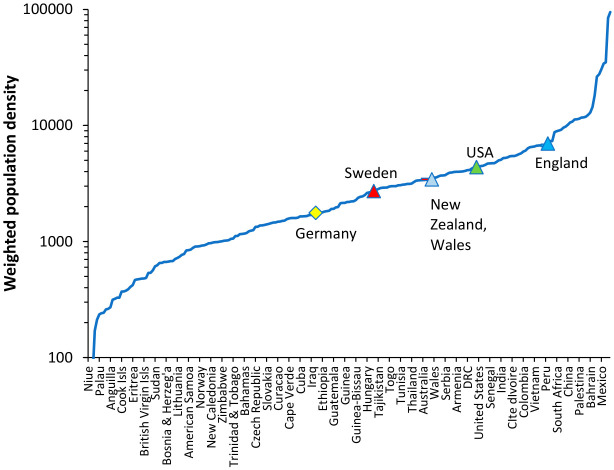
Weighted population densities of world countries. Data from [53]. The *Y*-axis is a logarithmic scale which has been truncated at 100. *Y*-axis shows every fifth country. DRC = Democratic Republic Congo.

The disparity in size has huge implications for capital costs and the cost per birth in each country. Figure A2a–c in Appendix A [25,26,29,30,31,32,33,34,35,36,37,38,39,45,47] present a wider array of countries with varying degrees of population dispersion. One measure of population dispersion is the weighted population density (WPD), which is calculated from the sum of the population density of each small area weighted by the population in that area [55,56,64]. The WPDs of world countries are shown in Figure A3 [64]. To the left of Figure A3, large hospitals become less common and ‘medical deserts’ more likely [71]. In Figure 4, England has the highest WPD followed by the USA > Australia > Belgium. Of relevance to Figure A2a in Appendix A, is that the WPD of England is twice that of Wales [50]. Countries with a WPD higher than England will be able to increasingly reap greater economies of scale as described by Erlang B in Supplementary Material S2. This applies to large cities such as greater London, which has a WPD some 44% higher than England and 183% higher than Wales [50]. See Table A1 in Appendix A, which shows a variety of geographies with both higher and lower WPD [50].

The shape of the distribution of maternity units in Figure 4 and Figure A2a–c in Appendix A is determined by two factors:

1. The distribution of the population with low density/highly dispersed areas having what are termed ‘medical deserts’ [71].
2. Historical factors leading to the choice to have more numerous but smaller hospitals. Examples include Belgium, Germany, Austria and the USA [1].

The USA is used as an example of these two factors, and a table of relevant metrics (including WPD of American states) is given in Supplementary Material S4 [27,28,29,48,49,52,53,54,55,56,57,58,59,60,61,62,63,64]; these are compared with norms from England [4,27,28,29,48,49,50,61,69,70], where data from England is compared to US states and counties to show how geography and history interact to influence hospital/department size and costs. England is used as the reference point because it has universal healthcare that is free to all citizens and has a relatively compact geography (as in Figure A3 in Appendix A), avoiding issues of limited access due to great distance.

Table S1 in the Supplementary Materials provides English levels of occupied beds (real-time LOS) for an acute hospital's (along with various population adjustment factors) paediatric and maternity departments (based on occupied beds per birth) applied to US states and counties. Note how many US counties cannot support a viable acute hospital and even less so a paediatric or maternity department. The situation for maternity and paediatric departments has been comprehensively covered by Interrante et al. [69] and Cushing et al. [70] along with maps showing the areas where there is a ‘medical desert’ in each state. One interesting feature includes the data for MRI scanners and scans per 1000 population in both the UK and the USA [61,62,63]. These data give an indication of how MRI scans are equivalent to a consumer product in the USA, leading to higher costs per scan.

Table S1 in the Supplementary Materials contains embedded formulae allowing countries other than the USA to add state- and county-equivalent data, which can then be populated with English equivalents. An example is provided for Milton Keynes in England which is around half the size of the District of Columbia (Washington DC) in the USA and contains just one acute hospital. Then, further down Table S1 in the Supplementary Materials, there comes a point where it is increasingly difficult to have a single hospital covering multiple specialties including maternity and paediatric units. Hence, line 1785 in Table S1 in the Supplementary Materials shows a 51-occupied-bed small hospital with just 2.1 occupied beds for a maternity unit and 3.5 for a paediatric unit. Some 45% of US counties lie below this point. The implications of the above issues are covered in Section 4. This presents a unique spatial problem because the optimum location for a hospital servicing these areas may not lie in the same state. Note the diversity of the trends in births at both the state and county level, which is covered in Section 3.6, Section 3.7, Section 3.8 and Section 3.9 below.

A relationship between size and costs for maternity units which approximated Erlang B was demonstrated in 2013, see L.21 in Supplementary Material S1. Hence, the Erlang B formula in Supplementary Material S2 and the ratio of beds per birth allows the size distribution in Figure 4 and Figure A2a–c to be converted into a national average cost per birth relative to England. This assumes that costs in small units are fixed and is addressed later. Hence, countries with smaller units, especially in the <1000 births category, will be structurally more expensive. As can be seen in Table 2, which presents a preliminary analysis, Belgium and Switzerland have an estimated cost per birth which is 42% and 46% higher than England.

Given the huge change in the ratio of beds per birth in the <1000 births category (Supplementary Material S2), the cost ratio is very sensitive to the distribution of individual unit sizes. England has the lowest proportion of births in this band at just 0.5% compared to 29.7% in Belgium and 37.2% in Switzerland. A single ratio of beds per birth was applied to each 1000 birth band for all of the countries other than the USA, which is sufficient to illustrate the concept that structural factors play an important role in international cost comparison. Further analysis is given in Section 3.14.

**Figure A4:**
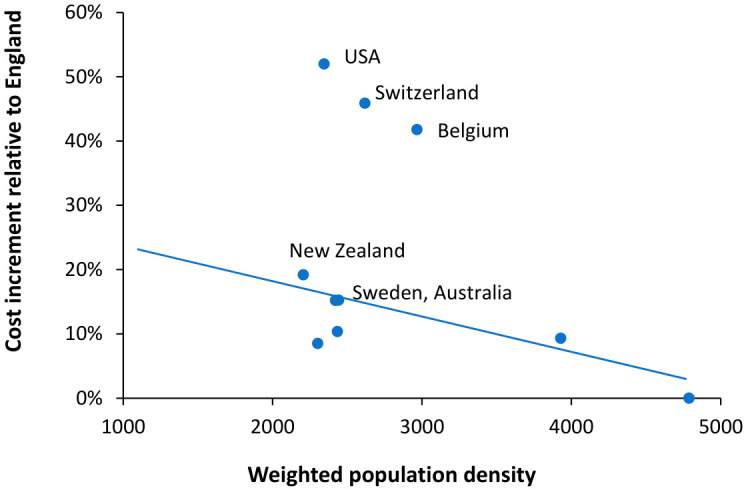
Effect of weighted population density and decisions regarding hospital size on the average price per birth relative to England. Data from [25,29,30,31,32,33,34,35,36,37,38,39] and Figure A2a–c. Note that additional cost weights (Section 2.3.6) were used for the USA due to the very high number of very small maternity units with less than 1000 births [25]. The unlabelled data points represent the four countries of the UK.

Figure A4 combines the factors discussed above into a unified link between WPD, choice around hospital size and the incremental effect on average cost per birth. The line in Figure A4 represents those countries which have attempted to maximize the size of hospitals. It must be emphasized that Table 2 and Figure A4 represent preliminary analysis, and further research is required which uses the size of individual hospitals and additional research on disaggregated unit costs, namely, direct, variable and overhead costs.

Other countries such as Switzerland, Belgium and the USA have chosen to have more numerous but smaller hospitals leading to higher average country costs per birth due to the size of units. Note from Figure A3 that Germany has a relatively low WPD (some 75% lower than England), which is one of the reasons why this country has a network of smaller hospitals leading to a very high number of available beds [2,72]. The resulting high ratio of available beds per 1000 population in Germany is therefore not a valid benchmark against which other countries should aspire.

Within the USA, only New York and Washington D.C. have higher WPD than England. On a world ranking, they would be eighth and ninth, respectively, with Macao first. New Jersey and Hawaii are similar to England, while Mississippi would only rank 206th in the world, similar to Greenland. In Table S1 in the Supplementary Materials, the largest county in Mississippi (Harrison) only ranks 334th and in England, would have a single hospital with around 19 maternity and 32 paediatric beds. Instead, it appears to have at least seven small hospitals [73].

One area to note is that the distribution of maternity unit sizes does not explain the very high healthcare costs in the USA. One possible explanation for this discrepancy lies in the data on MRI scans in Table S1 in the Supplementary Marterials [61,62,63]. The USA has a massive, 6-times greater number of MRI scanners per head of population than the UK, yet only generates 2-times more MRI scans per head of population. The difference implies that the capital and staffing costs per scan are 3-times higher in the USA. Indeed, are the 2-times more MRI scans per population medically justified or have MRI scans become a consumer product?

An interesting comparison is that the average cost per birth in the USA is $20,416 compared to $7313 in England (at $1.35 to £1), a 2.8-times difference [74,75]. Clearly, factors other than unit size are driving the difference. As a hint to the root cause of this problem in the USA, the two tabs “Minimum units by state” and “Size distribution county” at the far right in Supplementary Material S4 give an indication of the minimum number of maternity units achievable and the resulting average births per unit, and the resulting size distribution of maternity units if English standards of capacity planning were applied. The capacity planning process in the USA has been seemingly held captive to a set of historical factors, which further amplifies costs.

### 3.6. International Trends in Births

Given that both maternity and paediatric services are related by the trends in past and future births, such trends are an important aspect of capacity planning [3,4].

In the previous study investigating maternity bed capacity [3], it was suggested that births in England could rise by up to 24% higher than in 2023 over the next 15 years. An expanded birth forecasting tool is available in Supplementary Material S5 which is also relevant to paediatric capacity planning [4]. Supplementary Material S5 provides a library of trends in births at the local authority level in the UK. It is suggested that other countries compile similar libraries and employ tools such as artificial intelligence to attempt to generate estimates for future births at a local level. Greater detail has been given in the maternity study [3].

**Figure 5:**
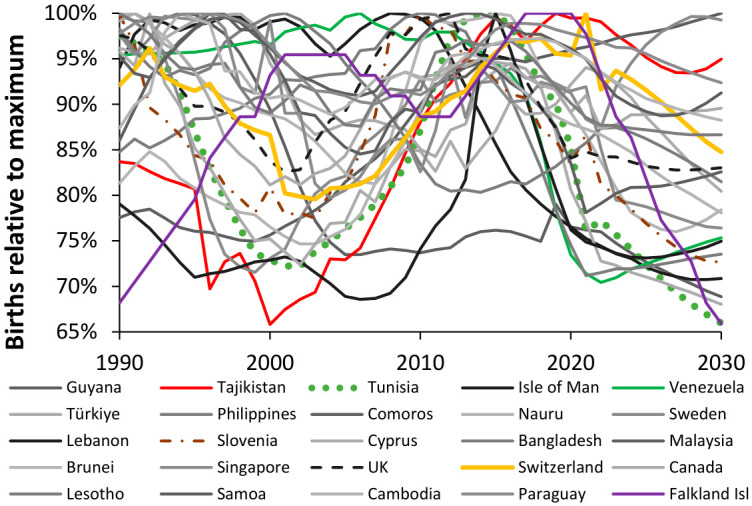
Trend in births relative to the maximum year for 25 world countries, 1990–2023. Data from [29].

It must be emphasized that the much-publicized declining fertility rates [76] do not necessarily mean declining numbers of births, and to this end, Figure 5 shows a surprising variety of non-linear trends in the absolute number of births since 1990 in 25 countries [40]. Births in each country are relative to the point of maximum births. Data between 2023 and 2030 are forecasts based on demographic trends and expected fertility rates.

It is clear from the multiplicity of trend lines that a wide variety of causes are simultaneously operating. As a point of comparison, the ratio of the maximum to minimum births for 239 countries ranges from just 1.03 in Bolivia to 5.8 in Montserrat, with a median value of 1.7. Values for the USA and UK are 1.16 (a 16% difference) and 1.21 (a 21% difference), respectively. The ratio for the world population is 1.11 due to high-growth countries (mainly Africa, Afghanistan, Iraq, etc.), counterbalancing those with declining births (mainly India, Nepal, Asia, South America, etc.).

Each country reflects a unique combination arising from the timing of previous peaks and troughs in births, government birth control policies, net migration, economic cycles, etc. [3].

Note the somewhat artificially smooth trends produced by the World Bank for the period 2024 to 2030 [40]. This issue was covered previously [3]. The key message from Figure 5 is not to assume straight-line trends; obtain suitably long-term data sources, and consider which forces may dominate in the future [3].

### 3.7. The Dilemmas Regarding Forecasting Future Births

**Figure 6:**
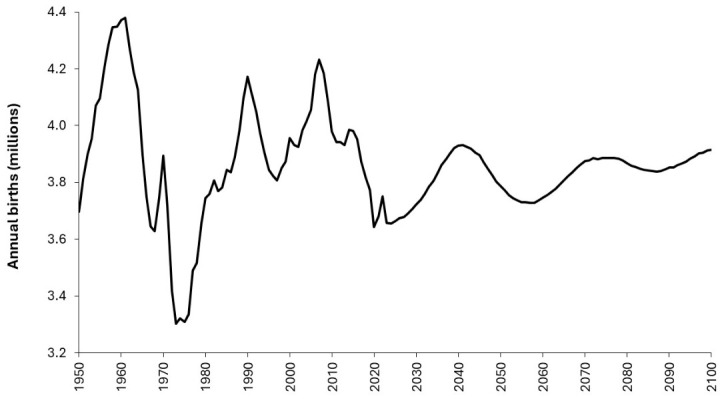
Trend in past (1950 to 2024) and future births in the USA. Data from [30] under a medium fertility rate scenario.

While it is true that the fertility rate is decreasing around the world [76], it is not true that the number of births is decreasing in every country (as in Figure 5) or location. The previous study on maternity capacity planning [3] devoted considerable attention to the unreliability of birth forecasts and the local factors affecting these trends. To illustrate these concepts, Figure 6 shows the trend in births for the USA between 1950 and 2024 along with a forecast to 2100 [41].

The trend for the USA encompasses the combined effects of past trends in births, immigration, birth control, and fertility rates, and how these have a knock-on effect in the present and future. It was previously noted that England has a similar cyclic pattern to the USA arising from the World War II baby boom [3], while Figure 5 suggests that cyclic trends are fairly common in disparate countries, although probably with different causes.

Note the jagged nature of the long-term trend. It has been proposed that infectious outbreaks (possibly working via the processes of pathogen interference) lead to intermittent fluctuations in the fertility rate and gender ratio at birth, see A.5–A.7, I.2, Q.4, R.13, R.14, and S.9 in Supplementary Material S1.

Each maternity and paediatric unit sits within a bigger national, regional and local context which must be understood to construct reasonable scenarios for future demand. National and state statistical agencies will have the relevant data and may be able to assist with local birth forecasts under various assumptions. Key factors will be approval for new dwellings and potential expansion/contraction of major local employers.

### 3.8. Local Trends in Births

**Figure 7:**
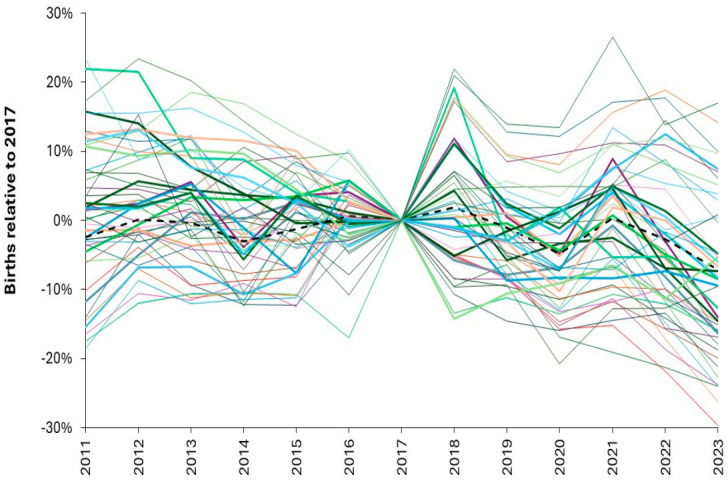
Trend in births for several Australian regions between 2011 and 2023 relative to 2017. Black dashed line is for Australia. Colored lines are for selected Australian regions. Data from [45].

To further illustrate the issue regarding local trends, Figure 7 shows the trend in births from 2011 to 2023 relative to 2017 in selected Australian regions.

Australia has around 300,000 births per annum (black dashed line). Most regions have over 1000 births per annum but the smallest region, namely, South East Tasmania, has less than 400 per annum. Figure 7 demonstrates the highly regional nature of birth trends and that even at the regional level, there is considerable volatility between years.

Even at the level of Australia (black dashed line), there are occasional minimum years, as in 2014, 2020, and 2023.

The minimum in 2020 (−3.7% compared to 2019) is repeated in other countries, despite an almost total lockdown in Australia including international travel, i.e., COVID-19 infection per se is an unlikely cause. In England and Wales, the minimum births in 2020 only commence from June 2020, i.e., conceptions after November 2019, which is before the arrival of COVID-19. Births stay depressed until the 12 months ending in June 2021 and then show a significant surge for the 12 months ending in February 2022, i.e., conceptions before June 2021. Hence, while tempting to invoke COVID-19, the real cause(s) may lie elsewhere.

However, even for Australian regions with 1000 births, the change ranges from −12% to +6% (1 STDEV of Poisson variation is ±3%). Changes for the other years will have various contributory factors.

**Figure 8:**
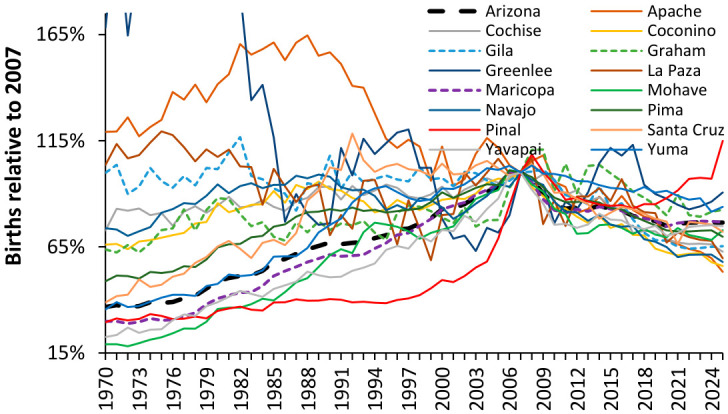
Trend in births for Arizona counties, 1970 to 2024 relative to 2007. From [27]. Note that 2007 is the year when births peak in the USA.

Finally, returning to the USA, Figure 8 [42] shows the trend in births for counties in Arizona, where trends are unlike that for the USA in Figure 6 and look to be dominated by population changes, especially in Pinal (5127 births in 2022, 31 persons per km^2^) and Greenlee (114 births, 2 per km^2^) counties. Since 2016, growth in Pinal is compensating for other counties. Note that the dispersion between counties is higher before 2007 than after.

Hawaii also shows a maximum in 2008 followed by a 22% decline to 2023 and no immediate sign of reaching a minimum [43]. The importance of local factors cannot be overstated. Table S1 in the Supplementary Material also shows trends in births in the USA at the state and county level. The birth forecasting tool in Supplementary Material S5, therefore, relies heavily on local knowledge, especially where there is growth in young families.

### 3.9. Using Births to Forecast Pediatric and Neonatal Admissions

Given that both maternity and paediatric services are related by births, Figure 1 [27] explored the relationship between the respective size of these units in the same hospital or region. In England, a paediatric unit has an average of 1.4-times the number of beds occupied as an associated maternity unit, with wider variation in this ratio as the hospital gets smaller or the comparison is made based on quarterly data.

The approach taken in S5 is to gather data on births at a local government level over a 20-to-30-year period or more, i.e., to establish a library of time-profiles which can be extrapolated using any number of methods. Since the UK has a strong cyclical pattern, this can be used to estimate the next cycle. This library of time-profiles approach can also be used for other countries such as those in Figure 5. In the absence of cyclical trends, a number of potential trends can be explored. Available government statistical agency forecasts should be considered to arrive at alternate scenarios. A moving 12-month total of local births can then be used to see which scenario looks to be the most viable.

Supplementary Material S6 presents one potential tool for forecasting non-maternity admissions such as paediatric (or other) demand. The main point of the forecasting tool is to force the use of a single year of age in the understanding of why paediatric and other demand is so volatile. The issue of single-year-of-age behaviour in paediatric deaths will be explored in the discussion. This method is suited to state/regional analysis, which relies on larger numbers where birth forecasts are more reliable. It can be used at the state/Area Health Board level to gain insight into the fundamental issues surrounding uncertainty in capacity planning and to issue guidance regarding possible future scenarios.

In ‘Forecast admissions’, data have been added for births to the residents of Milton Keynes in England where actual data are available up to 2023. Neonatal and paediatric admissions are merely example data from national ratios. Neonatal admissions assume an approximate ratio of one neonatal admission per seven births (see below). Each region will substitute their own actual births and admissions data.

Note how the forecast admissions rely on a cascade of ages arising from births. Hence, births in 2002 become the population of 1-year-old individuals in 2003, while births in 2011 become the population of 14-year-old individuals in 2025, etc. For the sake of simplicity, childhood deaths and inward/outward migration are ignored. The unit will also substitute their best forecast for future births (as alternate scenarios).

The aim of the sheet in S6 is to calculate a time series for admissions per birth/population by single year of age over 10 to 15 years to visualize the extent to which the trends in admissions show variation over time. The next step is to attempt to estimate how the admission rate will trend over time. In the example shown in the ‘Forecast admissions’ sheet, the maximum admission rate from the past has been chosen to estimate the likely worst-case years in the future. The worst case will not simultaneously happen for all ages.

The tab ‘Admission rate by age’ in S6 also shows that the variable admissions are dominated by the first year of life. This explains why the ratio of admissions per birth works as an international comparator and why the trend in births is so important. Gestational age along with other risk factors plays a vital role in admissions during the first 10 years of life and especially in the first year [77].

This approach has a major limitation in that its usefulness decreases as the size of the area decreases, giving results dominated by Poisson randomness in small units.

A highly recommended alternative is to substitute admissions with occupied beds or even occupied bed days. Since bed numbers are the goal, the occupied bed days (average occupied beds = bed days ÷ 365 days per annum) approach is highly recommended. This sheet gives an annual average and an adjustment for seasonality will be required which can be achieved by an analysis of past daily occupied beds, which is covered in the paediatric study [4]. As mentioned in the birth trends section, the difficulty comes when attempting to forecast the future.

### 3.10. Issues Specific to Neonatal Intensive Care

**Figure 9:**
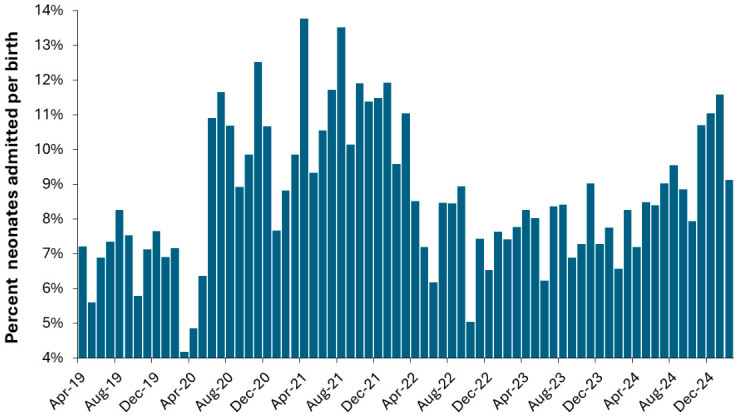
Proportion of births admitted to the neonatal unit at the Barts group of hospitals in London, England (2019 to 2024). Freedom of Information data provided by Barts Health NHS Trust.

Regarding neonates, Figure 9 shows that the proportion of births resulting in an admission to the neonatal unit shows systematic variation.

**Figure 10:**
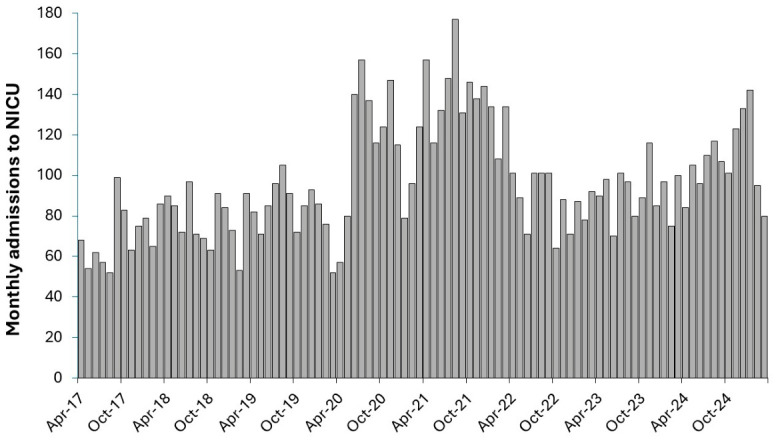
Monthly admissions to the NICU for the Barts group in London. Data provided via Freedom of Information request.

The actual monthly admissions shown in Figure 10 are the combination of births times the proportion progressing to the NICU.

While it is tempting to assume that the first peak in Figure 9 is due to COVID-19, it is important to point out that the timing does not exactly coincide, and the data do not reflect the minimum points in the summer for COVID-19 infections. It is not widely appreciated that COVID-19 had a profound effect on the frequencies of pathogens via pathogen interference [4], and that lockdowns only temporarily altered the transmission of different pathogens. In addition, many neonatal conditions originate during pregnancy, especially during the first trimester. Hence, births from April-20 onward will be influenced by events during the preceding 9 months of pregnancy. Before the arrival of COVID-19, pathogen outbreaks were already associated with subtle changes in the gender ratio at birth, still births and congenital conditions, see Q.4 and R.13 in Supplementary Material S1.

Figure 9 and Figure 10 also demonstrate that annual averages can be very misleading. Indeed, due to the systematic changes in the ratio of neonatal admissions per birth, a 12-month total will give different answers depending on when the 12-month period starts and finishes. This is called the calendar year fallacy, see M.28 in Supplementary Material S1.

Hence, the actual admissions to the NICU are a complex combination of the (variable) seasonality in births and the (variable) proportion of births progressing to the NICU. It would seem that irrespective of the trend in births, NICU admissions are increasing over time and show periods of high demand. The period of high demand during the first two years of COVID-19 should be investigated to see if the spectrum of diagnoses associated with admission was different from ‘normal’ [78]. However, admissions during the winter of 2024/25 were nearly as high as the two peaks during the first 2 years of COVID-19.

Note that the age in weeks for admission of premature neonates is decreasing over time as technology and medications improve [79], and that a higher proportion of births result in a NICU admission where the mother is aged over 30 years [79,80]. Very pre-term babies have a higher PICU admission rate in the first 2 years of life [79,80]. This brings us back to the issue of uncertainty about future demand and the need for flexible floor space to cope with future uncertainties.

### 3.11. Potential Roles for Pathogens in Maternal and Neonatal Morbidity/Mortality

It is well recognized that pregnancy leads to enhanced risk to mother and foetus due to certain pathogens and immune-related disorders [81,82,83]. For this reason, it is wise to scan the long-term trends for unusual events.

**Figure 11:**
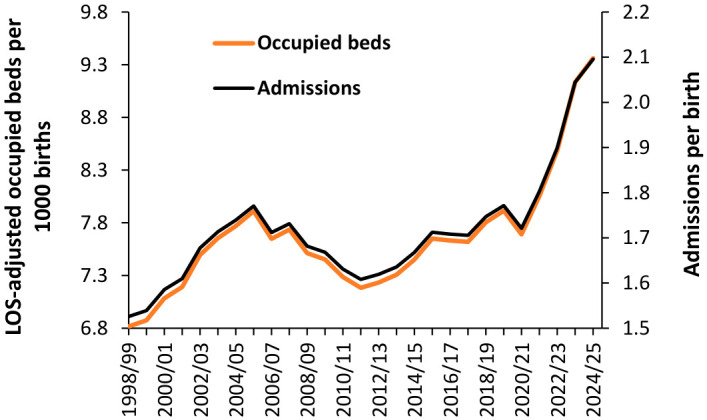
Trend in England (1998/99 to 2024/25) for obstetric and midwife occupied beds per birth after adjusting for changes in average LOS over time, and admissions per birth. Past avLOS was adjusted to the 2024/25 equivalent. Data from [49]. LOS includes time spent in labour and delivery.

Figure 11 shows the trend in admissions per birth and occupied beds per birth in the specialties of obstetrics and midwifery between 1998/99 and 2024/25 in England.

Occupied beds are the estimated real-time LOS and include an allowance of 0.5 day stay for any same-day admissions [3]. The occupied bed days were then adjusted for changes in avLOS over time. The avLOS for admissions underwent a reduction from 2.3 days in 1998/99 to around 1.61 ± 0.04 days from 2007/08 onward. In Figure 11, the majority of the same-day-stay admissions are for observation and tests during pregnancy. The proportion of same-day stays had a maximum of 43.9% in 2001/02, declined to around 41.8% in 2018/19 and then rapidly increased to 46% in 2024/25.

There is no reason for hospitals in England to collectively reduce obstetric and midwifery admissions per birth between 2005/06 and 2011/12. The introduction of the maternity pathway tariff in 2013/14 gives no financial incentive for the same hospitals to collectively increase admissions per birth so rapidly from 2020/21 onward.

Whatever the cause(s), infectious or otherwise, it leads to significant changes in the composition/case mix of costs per birth at different points in time and does not conform to simplistic explanations such as increasing maternal obesity. As far as I am aware, these trends have not been investigated. Figure 11 is a pertinent example of how the principle of the unexpected operates in the field of capacity planning, see Q.1–Q.18 in Supplementary Material S1.

**Figure 12:**
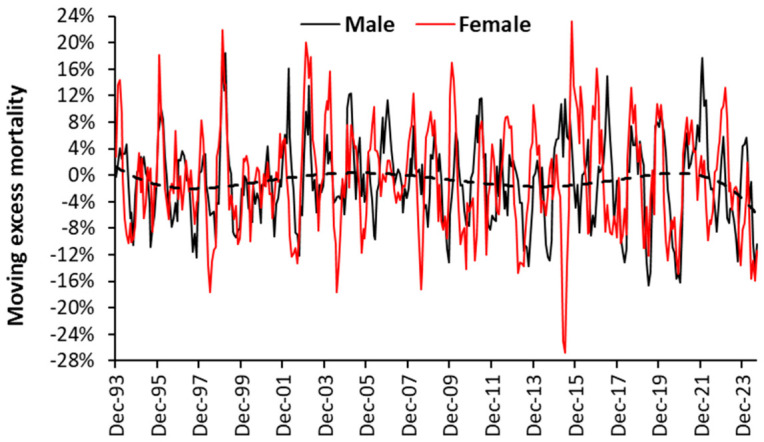
A moving excess-mortality calculation for infants in the first year of life in England and Wales. Data from [46].

Regarding the issue of potential roles for pathogens in neonatal morbidity, Figure 12 uses a moving excess-mortality calculation to locate periods of unexplained higher deaths. An excess-mortality calculation compares the average number of deaths in the most recent 4 months to the average for the preceding 8 months. Then, it moves forward by 1 month and repeats the calculation. A 4-month window gives time for any agent to spread across England and Wales. The majority of deaths in the first year of life are for neonates, usually in the first week of life.

As can be seen in Figure 12, the highest period for female deaths occurred in the 4 months ending October 2015 (+23%), while for males this occurred for the 4 months ending in March 1999 (+18%). The lowest levels of excess mortality for both males and females occurred in 2000 and 2001.

For females, a 4-month deficit in mortality occurred in the period ending in June 2015 which did not occur in males. Major deficits in female mortality all occurred in the 4 months ending in June or July.

This method has seemingly not been used to detect the impact of uncharacterized infectious outbreaks on females and males, either during the first trimester or after birth. The dotted line suggests that there may be long-term undulations in excess mortality.

There was no noticeable excess mortality during COVID-19 when paediatric admissions appeared to reduce [4]. As opposed to adult mortality, there is no clear winter-only peak in deaths. Epidemics are known to have seasonal patterns [84] and a multiplicity of pathogens seems implicated.

These hidden patterns will impact NICU bed demand and so it is highly recommended that NICU bed occupancy be followed at daily intervals for as long as each unit has data. Recall that 100% occupancy signifies too few beds relative to demand and these days need to be adjusted to estimate real bed demand. The data can be extrapolated using anticipated trends in future births and in neonatal health, due to maternal obesity and other factors [85], and fewer weeks since conception at which neonates are admitted. Given this uncertainty, it is advised that neonatal units be built with excess floor space to allow for unexpected disease outbreaks and trends in pre-term neonatal medicine.

### 3.12. Seasonality in Births and the Effect of Unit Size and Staffing Profiles

Seasonal profiles are well recognized in biology, human health and reproduction and are subject to metrological and social factors including major holidays [84,86,87,88,89,90,91]. The previous study [3] highlighted that births show seasonality, which will immediately impact neonatal demand and paediatric demand in the first year of life.

When approaching the quantification of seasonality, it is important to realize that there are two possible approaches.

1. Assume that seasonality has a fixed profile and hence, calculate averages for each month. This approach is most suited to planning staff numbers by month of the year.
2. Recognize that seasonality may show variability due to changes in all of the contributory factors. On this occasion, a moving 12-month calculation is more appropriate. This approach is most suited to determining the maximum size of the bed pool to cope with the seasonal maximum in births.

**Figure 13:**
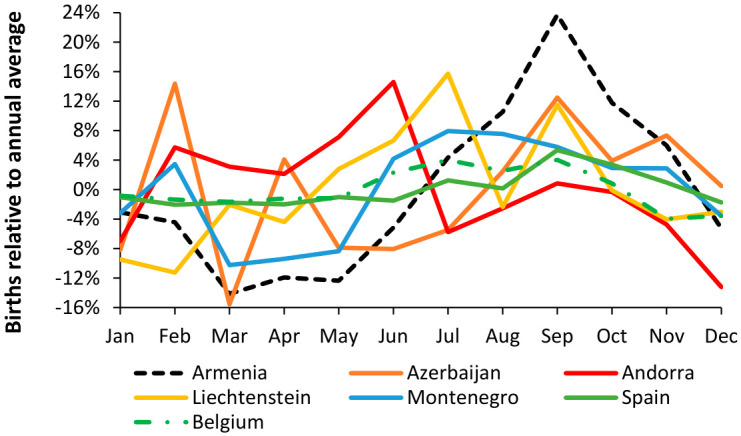
Seasonal average profile for births in several European countries (2006–2015). Data [44].

Figure 13 (using method #1) demonstrates that all European countries show unique (average) patterns in the seasonality of births and hence, each neonatal/paediatric unit should be aware that the local seasonal pattern will subtly affect bed demand and staffing profiles.

Note the diversity in the profiles with different months for the peak in births, different magnitudes for the peak and differences in the gap between the maximum and minimum, i.e., Armenia had the largest gap and Belgium the least. Across Europe, 4% of countries show the average maximum in February/July, 4% in June, 6% in July/September, 29% in July and 57% in September. The maximum in September may arise in countries with a strong Christmas/New Year holiday tradition, with December or early January being the month of conception.

Recall that this is an average and that the maximum birth rate in every year may not always be at the seasonal maximum average month. However, for staff planning for each month this approach is appropriate.

Systematic factors are clearly involved. The key point is that the local pattern of seasonality in births must be established for each neonatal/paediatric unit, with lagged effects in the paediatric unit, i.e., seasonality in births will have a 6-month lag for admissions for 6-month-old infants, etc.

**Figure 14:**
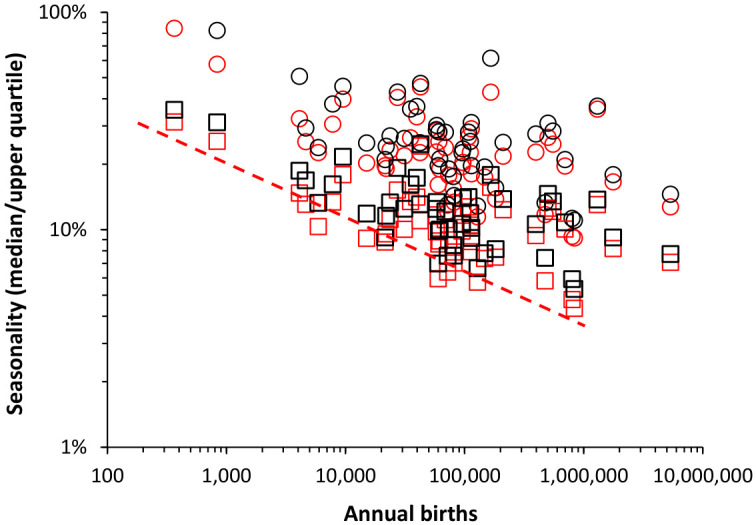
Results of a moving 12-month calculation of seasonality (maximum versus 12-month average), 2006 to 2015, with the median and upper quartile values shown for each country along with average annual births. Data from [44]. Circles (o) are for the ratio of maximum to minimum, squares (▫) are the ratio of maximum to the 12-month average. Red signifies the median while black signifies the upper quartile. The red dashed line shows the trend expected from Poisson statistics, i.e., statistical variation without additional country-specific social- and environmental-based variation.

Figure 14 (using method #2) demonstrates that the apparent seasonality depends on the geographic size of the country. In Figure 14, the data on the extreme right are for the European Union. The dashed red line shows the minimum possible seasonality and a log-log decline as size increases. A more homogeneous population mix may account for some of the lower percentage increase values in some countries such as Finland, Belgium, France, etc. Countries of large geographic size such as Germany, the Russian Federation and Ukraine can be expected to show region-specific profiles. At the local level, small-number Poisson variation adds to the volatility in the seasonal profile.

**Figure A5:**
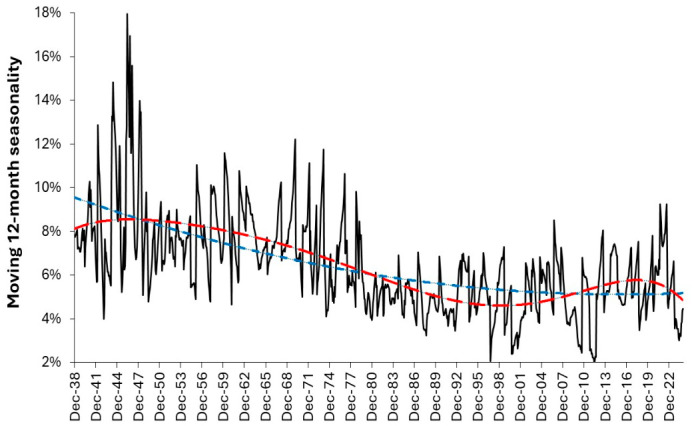
A moving 12-month seasonality calculation for monthly births in England and Wales after adjusting all months to 30.44 days per month. The blue dashed line is a simple second-order polynomial, while the red dashed line is a sixth-order polynomial indicating potential longer-term cycles.

It should be noted that seasonality is not only volatile but is changing over time. For example, Figure A5 in Appendix A shows a moving 12-month seasonality calculation (maximum month versus 12-month average) in England and Wales from 1938 onward. The dashed line in Figure A5 is a polynomial curve fit. The ratios of the maximum to minimum and the maximum to the average births had the highest value of 38%/18%, respectively, in 1946, a local maximum of 23%/12% in 1970, and another local maximum of 21%/9% in 2021. An all-time minimum of 5%/2% occurred in 2012. More recently, in 2024, there was another minimum of 7.6%/3.0% (data from [3]). The ratio appears to decline through to the 1980s and may have reached an asymptote since then. Note that before 1978, February is the most common month for the seasonal maximum and then this shifts to around July to September in the 2000s, with the 1980s and 1990s showing a transition between the two. Seasonality also seems to go through a minimum in the 1980s. Such longer-term patterns are poorly understood or investigated.

Given that births in the majority of maternity units fall below 10,000 per annum, the minimum possible seasonality in Figure 14 is 10% more than the annual average at 10,000 rising to 20% at 1000 births.

It is suggested that for the calculation of bed numbers, the upper quartile from method #2 may be the appropriate figure to use in the two bed calculators in Supplementary Material S2.

Supplementary Material S7 gives a summary of the seasonality calculations for European countries between 2006 and 2015 [44]. Note how a calculation based on the average for each month relative to the calendar year average (method #1) gives a lower value for apparent seasonality than the moving 12-month method.

### 3.13. Length of Stay (LOS) and the Benchmarking Fallacy

This section is very important because there are many fallacies surrounding LOS benchmarking and the supposed reduction in costs when LOS is reduced. Detailed analysis of LOS is therefore a very important defensive step, since there is a widespread perception that LOS ‘should/ought’ to reduce ad infinitum. While LOS did reduce somewhat rapidly during the 1970s and 1980s, the rate of reduction dramatically reduced from the 1990s onward [4].

In LOS benchmarking, it is often insinuated that “your LOS is higher than the national average, and therefore you must be inefficient and could save x% beds by moving to the national average”. This can be called the steady-state fallacy, i.e., LOS is only of primary importance in bed demand when admissions and case mix are at a steady state and assuming that all socio-economic, environmental, and infectious factors are identical to the ‘average’.

**Figure 15:**
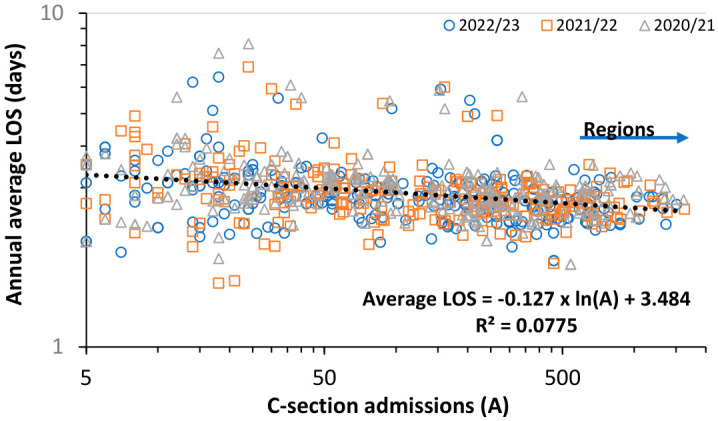
AvLOS and admissions for C-section at Australian hospitals over three consecutive years (2020/21 to 2022/23) [47]. Both avLOS and admissions are a log scale.

Figure 15 shows annual average admissions and avLOS for caesarean section (C-section) in Australian hospitals over three consecutive recent years, and illustrates the concept that avLOS may be unique to each hospital with its admission thresholds (private/public, size, etc.), surrounding community (socio-economic, ethnic, cultural, social group, travel distance) and how these interact with government structures (health policies, health authorities, social services, etc.). This system also interacts with the meteorological and infectious environment leading to simultaneous variation in admissions and avLOS.

In Figure 15, LOS is measured at midnight and avLOS is only calculated when there are five or more admissions and has been truncated to remove unusually long stays. As can be seen, there is a trend to higher avLOS as unit size reduces; however, the dominating factor is the high annual and systematic scatter around this trend, hence, the low R^2^. The majority lie at an average below 3 days, but a subset lies above 3 days.

It is my opinion that no benchmarking tool exists to adjust for the simultaneous interaction between all factors influencing avLOS. It is merely interesting, but not prescriptive, to attempt such comparison.

**Figure 16:**
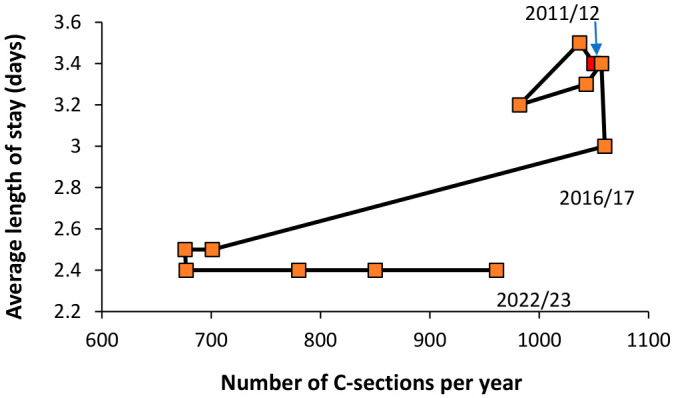
Average length of stay and number of C-sections per year at the Mater Women’s Hospital in Brisbane, Australia [47,92]. The arrow indicates the start of the time series.

In the case of C-section, the question as to whether the procedure is clinically justified is of greater importance than arguments around avLOS. This is illustrated in Figure 16 for C-sections per year at the Mater Women’s hospital in Brisbane, Australia [47,92]. As can be seen, prior to 2016/17, there were typically >1000 per annum at an average LOS of between 3.2 and 3.5 days stay (using midnight occupancy). In 2016/17, the number of C-sections remained >1000 per year but avLOS had declined to 3.0 days, followed by a massive shift to both lower avLOS and procedures per year. However, the number per year has been steadily increasing since 2019/20. Note that the data in Figure 15 covers the years after the transition to a lower avLOS at this hospital.

There are several issues relating to this data.

1. LOS is measured at midnight and avLOS must be calculated using real-time data [3].
2. It requires feedback from the mothers regarding their perception regarding the benefit of this change.
3. Why has the number of C-sections been increasing since 2019/20?

If decreasing LOS is in the interest of the patient, then it should be pursued because the focus is on the patient. However, such reduction may require changing both the hospital and surrounding social systems.

As an example, many hospitals now run ‘fit for surgery’ and ‘fit for pregnancy’ programs where patients attend managed fitness/nutritional classes to ensure optimum recovery after elective surgery or delivery. In the case of children, nutrition prior to surgery would probably be the main focus.

### 3.14. Unit Size and Cost per Patient

**Figure 17:**
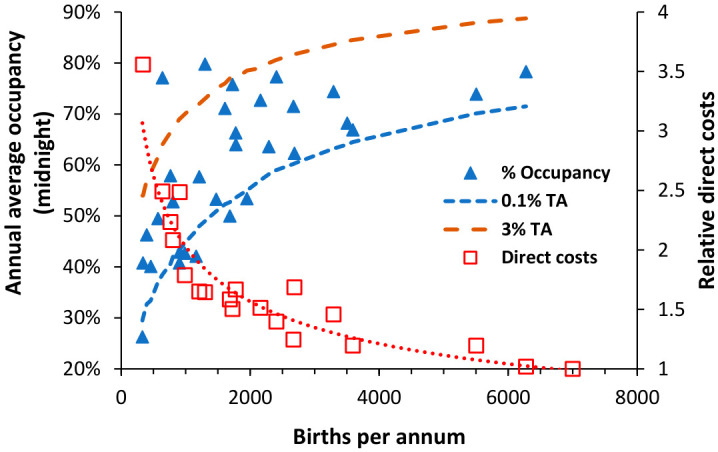
Annual average occupancy and direct cost per birth for US maternity units. Data from [93]. The direct cost line excludes units running at high average occupancy. TA = turn-away.

Reanalysis of the data of Thompson and Fetter [93], which covered 33 obstetric units in Connecticut, USA, in the early 1960s, is shown in Figure 17. Note that this study contains high scatter regarding direct costs because each of the 33 hospitals may have used different methods to allocate ‘direct’ costs.

In Figure 17, the trend for size and average occupancy follows Erlang B at 5.8 bed days per birth between 0.1% and 3% turn-away. A figure of 6 bed days per birth would shift the lines of turn-away lower on the chart, etc. Recall that these data were in the early 1960s, hence, higher bed days per birth. However, the relationship between size and direct costs can be modified by running smaller units with a higher average occupancy. This is prevalent below 3500 births per annum, i.e., where smaller size places pressure on costs, which is then compensated for by increasing average occupancy. This strategy is used in the absence of the awareness that turn-away may have deleterious consequences.

A similar situation was observed in Belgium [37,38,39], where some smaller units operated at large-hospital-equivalent bed occupancy in an attempt to mitigate the effects of small size on higher cost [37]. In the absence of the births-to-beds calculator (Supplementary Material S2), this behaviour was considered ‘normal’. Note also that the minimum economic size estimated for Belgian maternity units of 557 births per annum (1.6 per day) [38,39] corresponds to the point where the power law relationship of size and costs rapidly escalates. Note that the power law relationship in Figure 17 approximates to the beds per birth ratio given in Supplementary Material S2, indicating that direct costs are subject to the same forces. Figure 17 has excluded data from small units running at high occupancy and excluding these data generates a relationship between relative cost and average occupancy:

Relative direct cost = 0.942 − 1.385 × ln(average occupancy), where r-squared = 0.61

Average occupancy is a decimal, so the log value is negative and lower average occupancy adds to the relative direct cost (as expected) while higher average occupancy is able to mostly mitigate the effect of the smaller size of costs. This relationship implies that increasing average occupancy from 26% (equivalent to around 300 births p.a.) to 80% (around 7000 births p.a.) effectively reduces costs by around 45%, irrespective of size. The effect of this overriding relationship upon patient safety has not been investigated because it generates the adverse effects of turn-away.

However, the above trends were from the USA, where there can often be more than one obstetric unit per town or city. The authors noted that one small unit managed to operate at high average occupancy because a larger unit also operated in the same town [93]. Hence, when the smaller unit hit 100% occupancy the higher turn-away was counterbalanced by diverting patients to the larger unit. The smaller unit was effectively using the ability of the larger unit to intermittently absorb patients, leaving the small unit to run at a very competitive low cost per patient—which effectively mimicked allowing a part (say one ward) of a larger (combined) unit to operate at high occupancy. This is a ‘cunning’ but somewhat parasitic relationship.

The relevant point here is that when there is only one unit per town, then smaller size looks to incur both higher capital and staff costs. Once again, around 0.1% turn-away looks to indicate somewhere close to good practice.

## 4. Discussion

International capacity planning can be understood from three perspectives.

1. WPD, which regulates the distribution of hospital/unit sizes within a country.
2. Poisson statistics and the Erlang B equation, which explain the relationship between size, bed occupancy, and patient turn-away and hence, costs.
3. How the history of healthcare development in each country can complicate current attempts to reduce costs.

The hidden complexity behind capacity planning in small units has been illustrated using pregnancy, childbirth and paediatrics as examples. Diverse examples have illustrated the multi-dimensional aspects of uncertainty and complexity, which imply that a flexible design approach is required. Several key points will now be discussed.

### 4.1. Fundamental Roles for Weighted Population Density (WPD)

WPD is somewhat akin to the missing piece of the international hospital capacity planning jigsaw. WPD answers the question posed in part 1 of this series [1] as to why countries with identical per capita wealth have such widely disparate levels of bed supply. WPD has already been linked with infectious disease spread [56,94,95,96,97] and there is a logical relationship between hospital capacity and the direct and indirect effects of infectious diseases.

WPD may have hidden associations with other factors. For example, in the USA, the states with the lowest WPD tend to have the highest crude birth rates (births per 1000 population) and the highest ASMR, although there are exceptions (see Supplementary Material S6). There may be further explanatory factors relating to poverty and the ability to pay for health insurance. Low WPD states will mainly be agriculture based with the additional complication of exposure to herbicides and pesticides.

Table A1 (Appendix A) showed considerable variation in WPD across England and Wales with all cities containing larger and more numerous hospitals. Supplementary Material S6 gave WPD at the state level in the USA and for the 10 highest WPD cities and provided a framework to estimate the capacity required in each location using ratios from the English NHS as a preliminary starting point.

Table 2 and Figure A4 in the Appendix A made a preliminary attempt to link WPD with the average cost per birth in different countries. Figure A4 also attempted to highlight which countries had gone down historical routes leading to a proliferation of small hospitals and hence, higher costs as per the estimate in Table 2.

WPD has a direct impact upon the issue of medical deserts and the spatial analysis conducted relating to maternity and paediatric unit availability has a common link [69,70] demonstrating that artificial county/state/regional borders need to be ignored to tackle this serious problem.

These preliminary findings suggest that greater international research can be profitably directed into this area.

### 4.2. The Fundamental Role of the Trends in Births

The previous study discussed the uncertainty surrounding birth forecasts [3] and highlighted that such forecasts, especially those using the total fertility rate (TFR) methodology, were (in hindsight) extremely unreliable simply because TFR follows complex time trajectories. Traditional fertility rate-based forecasts, as for the USA in Figure 6, typically appear to underestimate the future and lead to forecasts which are overly smoothed, compared to the more peaked and sometimes erratic behaviour seen previously. Three-parameter models have been shown to give a flexible range of more realistic forecasts [98,99,100,101,102,103,104]. Unfortunately, such models require assistance from government statistical agencies who may be unwilling to do such analysis—perhaps not wanting to challenge the widely used TFR-based forecasts.

Uncertain/unreliable birth forecasts then invalidate subsequent maternity, paediatric and neonate population forecasts. The pragmatic and simple methods given in [3] and Supplementary Material S5 allow any unit to construct alternative scenarios for future births and maternity/paediatric demand.

The whole idea is to derive a set of realistic forecasts which tend to focus on the maximum to the above-average cases. The best way to prepare such forecasts is to use a moving 12-month total using historical data. Then, add the alternative forecasts and compare these with the actual emerging trend. Plans are made (with flexibility) based on the alternatives. The actual births are then compared to the forecasts to derive the best-case scenario just prior to commencing any construction or refurbishment and flexibility is deliberately incorporated into any physical space constructed.

### 4.3. Seasonality and Circadian Patterns

It is known that the seasonality of births varies according to the mother’s age, education, social group, parity and geography, plus additional factors [87,88,89,90,91]. Figure 13 shows that the profile across the year for births differs by country, while Figure 14 demonstrates that the seasonal maximum in each country shows different timing and magnitude. The USA may realistically be considered as a collection of 50 countries. This suggests that the seasonality profile may be specific to each paediatric unit and this needs to be confirmed. The analysis of births in England and Wales demonstrated that the seasonality of births shows long-term trends with associated high volatility (Figure A5 in Appendix A). Volatility in any form drives the real-world bed occupancy margin [2].

Seasonality in births, or more correctly, seasonality in conception [87,88,89,90,91], is clearly more important for neonatal demand profiles, but will also impact paediatric demand during the first year of life as a delayed time cascade.

This form of seasonality will then interact with other factors such as temperature, pollution, weather types, infectious outbreaks, etc. [105,106,107,108], all affecting those of different ages to different degrees. Once again, this suggests that the actual profile of bed demand will vary by location and needs to be confirmed using actual data.

It is of interest to note, from Figure 13, that Belgium has numerous small maternity units; however, the relatively flat seasonal profile for this geographically small country partly mitigates against the small size of the units [37,38,39]. From Figure A5, the calculation of seasonality should be restricted to recent data from 2000 onward.

Regarding the circadian cycle for births, research over many years has shown a peak in normal spontaneous delivery after midnight and around 2 am to 5 am, although induction of birth has altered this pattern to between 8 am and 4 pm [108,109]. There is evidence to suggest that C-section rates may be higher on high workload days [110]. Levels of induction and C-section may indicate resource constraints rather than clinical need.

### 4.4. Unit Size (Beds), Occupancy and Turn-Away

Issues around size and occupancy have been previously discussed [2,3]; however, the Erlang B formula has been widely applied over many years to determine adequate bed numbers [6,7,8,9,10,11,12,13,14,15,16,17,18,19,20]. The surprising thing is that government health departments fail to use it as a capacity/quality/safety measure. Is this an effort to avoid public scrutiny?

The concept of turn-away lines was first introduced in 2001, when it was used to document turn-away in different types of English hospital bed pools, see L.2 in Supplementary Material S1. Then followed three discussion documents (L.3–L.5) regarding its application, and a study applying them to emergency admissions (L.6), a study on psychiatric beds (L.22), three studies for maternity (L.19–L.20) and several studies for critical care (L.29, L.41, and L.55). An important study in 2011, see L.12 in Supplementary Material S1, used lines of turn-away to understand whole-hospital occupancy in England and the USA. This study emphasized that whole-hospital occupancy was the weighted sum of individual department occupancies. American hospitals with <30 beds effectively had only two bed pools while at 200 beds there were effectively five bed pools, i.e., the weighted equivalent to five bed pools.

Figure 2 gives the occupancy at 6 a.m. while occupancy is often measured at midnight. As pointed out by Riahi et al. [111], bed occupancy also varies by the day of the week, and the time of the day such that measurement of hourly occupancy becomes a part of genuine capacity planning.

Hence, Erlang B is good enough to provide clarity, to scan for flawed data, to conduct what-if scenarios and to ask awkward questions such as why does our unit operate at such higher turn-away compared to everyone else [2,3,4]?

The major observation is that while Erlang B was designed for a specific situation, the lines of turn-away act as a framework which applies in wider circumstances than its original intended purpose. Section 3.3.2 used a variety of real-world situations to establish the method's wider applicability. Figure 17 demonstrated that in the USA, maternity bed occupancy lies in the range of 0.1 to 3% turn-away, with the higher figure arising from the common situation where there is more than one unit in a town/city. In this situation, expectant mothers can be directed to the unit having spare beds.

For most unscheduled care applications, 0.1% turn-away represents a sensible starting point. Units can operate at higher turn-away where there is considerable transfer to other units or where there is a mix of scheduled/unscheduled care. When I was developing the method, my conclusion was that around 3% turn-away approximated an acute hospital unit with mixed unscheduled/scheduled care. Scheduled care does not magically remove the fact that referral to acute outpatient care is itself subject to seasonality and Poisson variation, see D.1–D.12 in Supplementary Material S1. The highest turn-away lines will be reserved for instances where a receiving unit operates on the basis of one-out triggers one-in, such as a stand-alone rehabilitation unit. As a technical note, always compare like with like function, i.e., level I critical care versus other level I units, and if possible, use the same time period, i.e., month, quarter, or year.

An excellent example of the effect of turn-away comes from a study on waiting time in the paediatric ED, often called access blocking [112]. In a 347-bed paediatric tertiary hospital (whole-hospital average occupancy of 68% at 0.1% turn-away), when inpatient paediatric occupancy was at or more than 80%, every 5% increase in hospital occupancy was associated with an increase in length of stay of 18 min for discharged patients and 34 min for admitted patients. With a 5% increase in inpatient occupancy, there was an increase in the odds of either a patient leaving without being seen OR = 1.21 or being treated in a hallway bed, OR = 1.18. Unfortunately, this author was unaware of the calculation of turn-away and presenting the data against lines of turn-away would have been extremely useful and allowed extrapolation to other sized units.

Another study involving 116,235 paediatric admissions for 19 common conditions in hospitals across the states of Pennsylvania and New York showed that admission day occupancy (crowding) in the paediatric units affected the LOS for less complex conditions [113]. On this occasion, percent occupancy is used as a proxy measure for busyness.

Further to the deleterious consequences of turn-away, a 2013 National Audit Office [67] report conducted during the 2012 peak in births in England showed that 12% of maternity units were capping annual total births, while in the period from April to September 2012, some 28% were closed to admission for more than 12 h, with an average closure of 3 days. Some 11% were closed to admission for >14 days [67]. Considerable delays to admission will have occurred as mothers are diverted to alternative maternity sites. Supplementary Material S3 demonstrates how the line of turn-away can identify those units most affected. The birth-to-bed calculator in Supplementary Material S2 could have been used to proactively avoid this situation. Indeed, such problems still persist in 2026, with over 40 units having inadequate beds even at the point of minimum births [27,28], as inaction has prevailed over many years.

The key concept from Figure 3 is to compare your unit with other similar units using the lines of turn-away to illustrate higher operational chaos as turn-away increases. An adequately resourced unit will have an annual average occupancy rate consistent with around 0.1% turn-away or lower. However, as explained previously [2,3,4], for something like a children’s hospital with a high level of elective surgery, it may be possible to operate at 3% turn-away if there is a considerable amount of ‘routine’ surgery which is not overly time-critical. The same applies to critical care units, which can become the rate-limiting step even for emergency/urgent surgery.

The suggested closure of the 17 smallest maternity units in Belgium [38,39] would represent an ideal application of the births-to-beds calculator as it would enable managers in the ring of hospitals surrounding each of the smallest units to calculate if the additional births at their unit would necessitate additional resources (beds/staff).

Supplementary Material S2 also includes a births-to-beds calculator at 3% turn-away. This is because small maternity units tend to operate above the 0.1% turn-away line. This is probably driven by financial necessity; however, 3% turn-away is probably the maximum recommended. Up to the present, there has been no research regarding the interplay between staffing ratios and turn-away and the risk of adverse outcomes. The suspicion is that staffing takes precedence, because on many occasions a healthy baby and mother can be discharged early if a bed is needed. However, in the labour/birthing unit closer to 0.1% turn-away would seem sensible.

As a final comment, Erlang B assumes a constant average arrival rate; hence, it needs to be applied relative to the arrival rate for the different seasons/periods as in Figure 11. Most neonatal units simply cannot have enough beds to achieve 0.1% turn-away at the infrequent points of maximum demand which may occur during an infectious outbreak. The huge spread in occupancy and turn-away in English units is symptomatic of a poor planning process [2,3,4].

The most pragmatic solution is to look at the daily arrival rate (as admissions, not discharges) over many years and attempt to be at a high occupancy level, perhaps close to 100%, during those infrequent high events. This is a risk assessment judgement which balances the capital costs of the floor space and physical equipment against the frequency of such events. This also raises the issue of how do you staff a unit in the face of volatile demand? But first, we need to understand the definition of a small unit.

Regarding the size of maternity units in the USA, supposedly commencing at one birth [25,69], it should be noted that rural areas of the US without access to an obstetric unit have increased levels of births in community hospitals without birth facilities [69,114,115].

### 4.5. Poisson Variation Is a Hard Taskmaster Especially to the Small Unit

It has been observed by DeSisto et al. [25] that in the USA, the size for a maternity unit commences at a nominal one bed and that 55% of maternity units handle fewer than 1000 births per annum. So, how do we define a small unit? Poisson statistics describe the variation around the average for integer events (patients) relating to their average daily arrival rate. This has been used extensively for over 100 years in epidemiology and capacity planning [6,7,8,9,10,11,12,13,14,15,16,17,18,19,20]. At high numbers (generally >100 per unit of time), it can be approximated by a normal distribution but at smaller rates, there is an increasingly skewed distribution. While the standard deviation is always equal to the square root of the average arrival rate, there is a minimum of zero, the average and the average minus one are the most common arrivals, and to compensate for the minimum of zero there is a tail of higher arrivals possible per unit of time. The tab ‘Poisson at x per day’ to the far right in Supplementary Material S2 shows the distribution of arrivals at an average of four or eight per day.

How does this dictate the definition of a small unit? At an (assumed constant) average of eight arrivals per day, one standard deviation (STDEV) is equal to 8 ± 2.83 arrivals such that zero or one arrival occurs about once a year, as do 16+ arrivals. Both seven and eight arrivals occur on around 51 days each per year. If the avLOS is 2 days (calculated at midnight), there will be an annual average of 16 occupied beds, and at an average occupancy rate of 80% (a high figure), the unit has 20 beds. What do you do on that 1 day when 16 patients arrive, and you already have 16 occupied beds? You can immediately admit the four most acute patients and the other 12 must wait in a queue (with triage). You can possibly arrange a hasty discharge for eight patients, leaving four still waiting.

Hence, the reasoning is that eight arrivals per day (around 3000 births per annum) represent a small unit, which lies at the upper quartile of units in the USA [25], i.e., 75% of US maternity units are small to very small. This illustrates the importance of size and the lines of immediate turn-away in Figure 3. The USA has far lower WPD than England and this is a common problem for many countries such as Australia, large parts of Africa and even the rural parts of India and China. The result is that capacity planning in most US maternity units is dominated by Poisson-based chance variation in admissions.

The supposed economic minimum of 557 births for Belgium [38,39] is only 1.5 per day, hence, zero arrivals on 79 days per year, one arrival on 121 days, two arrivals on 93 days and 6+ arrivals on 1.8 days per annum. At this number, there is no possibility of full obstetric consultant cover and it most probably represents a midwife-led unit handling exclusively the lowest-risk normal deliveries. Similar calculations were conducted using the 1963 study based on US maternity units [93], as shown in Figure 17.

The study of DeSisto et al. [25] shows that 20% of births in the US occur in maternity units with <700 births per annum, while 80% of US maternity units have fewer than 2000 births per annum. Units with >7000 births per annum only account for 1.2% of hospitals. The above definition of eight per day, i.e., around 3000 births per annum, places around 90% of US births in small to tiny units. Figure 5 demonstrated a similar picture for paediatric units in the USA while Kozhimannil et al. [114,115] associate higher adverse outcomes with small maternity units and the same would be expected for smaller paediatric units.

Monte Carlo simulation and other operational research tools, which will partly rely on Poisson statistics and Erlangs equations, can therefore be employed to examine whether staffing and bed occupancy benchmarks are likely to work in the real world [116].

The situation in the USA is further compounded by the operation of a ‘free market’, some may call it a free-for-all, where hospital chains compete for market share resulting in multiple hospitals in each city/town where a system of rational planning would only have one larger hospital with consequent lower costs per patient. See Supplementary Material S4. As observed in England, the minimum size for a consultant-led obstetric unit is around 10 beds plus additional birthing beds.

### 4.6. Benchmarking avLOS

Before discussing avLOS, it is important to dispel misunderstanding regarding its role in bed demand. In elective/scheduled care it is absolutely true that reducing avLOS reduces bed demand and increases throughput per bed and therefore reduces the capital cost per patient and increases income per bed. Hence, implement a full range of patient-centred strategies to achieve this aim. While such strategies can also be applied to emergency/unscheduled care, they have less impact on bed demand because it is the volatility in admissions and seasonality which sets the required number of beds and their average occupancy. Reducing avLOS is thus appropriate for all of the elective/scheduled aspects of wider adult care. Since maternity and paediatrics are mostly emergency/unscheduled care, the capacity planning process requires different thinking.

Interestingly, from 1998/99 onward, the number of occupied beds in the NHS stayed approximately constant, leading to escalating bed occupancies as the supply of beds declined as the PFI hospitals replaced the previous old hospital buildings [1,2,3,4]. This then created a somewhat obsessive need to decrease LOS by employing aggressive LOS benchmarking between hospitals. The lowest LOS was always declared to be the best/optimum LOS. The problem is that this type of benchmarking can lose sight of what is best for the patient—within a wider social context for children or mother/baby in maternity.

Figure 15 used Australian data for C-section to show high variation between units of similar size but in different parts of Australia. Any benchmarking requires far greater understanding of the complex contribution from societal, social group, distance, size and pressure on units with too few total beds.

### 4.7. Forecasting Future avLOS

**Figure A6:**
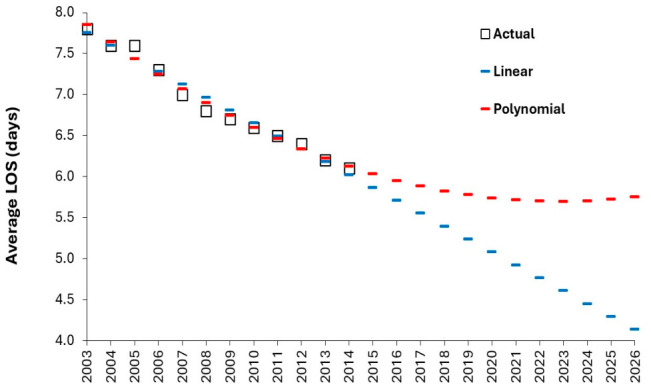
Trend in average LOS for C-section in Belgium (2003–2014) and extrapolation to 2026 using a simple linear trend or a second-order polynomial. Data from the study of Van de Voorde et al. [43].

One of the bigger dilemmas in capacity planning is forecasting future avLOS. In a world where reducing costs is the holy grail of governments there is a perverse incentive to devise reasons why future avLOS is going to be lower. Having built hospitals with too few beds, this becomes self-fulfilling, with higher bed occupancy and turn-away ensuing. Figure A6 illustrates some of the dilemmas using data from Belgium (2003–2014) covering the trend in avLOS for C-section. Two scenarios have been applied, namely, a linear trend or a polynomial trend. By 2026, the linear trend predicts an avLOS of 4.1 days while the polynomial trend gives 5.8 days. Note that there is no international norm for the avLOS associated with common maternity outcomes [66].

In the face of rising obesity among mothers [85] and associated complications, common sense would be to investigate a polynomial relationship. In the extreme, linear forecasts always end up extrapolating to 0 days. Part 3 of this series highlighted the dangers of pursuing lower and lower LOS [3].

### 4.8. The Illusionary Effect of LOS on Costs

Having established that the primary driver for bed occupancy is the volatility in admissions, it is apposite to investigate some of the myths surrounding LOS and costs [117,118,119,120,121,122,123,124]. Logically, any disease/procedure dictating an extended stay will have a higher cost; however, this does not automatically mean that reducing LOS will make a significant reduction in cost. In 1970, Lave and Lave [120] pointed out methodological difficulties and pitfalls for costing in a multiproduct setting such as a hospital. These have likely been ignored in the rush to demonstrate large savings. There are numerous studies claiming large reductions in cost by reducing LOS. However, the suspicion is that most of these studies have made erroneous assumptions around the behaviour of costs in the real world.

Firstly, most studies are conducted in large hospitals, which allows for higher average bed occupancy and diminishes Poisson randomness. Hence, the hidden assumption is that costs in smaller hospitals behave the same way as in larger hospitals. This has not been investigated.

Perhaps one of the most important studies demonstrated that the cost elasticity for the effect of reducing avLOS on costs in the USA was very low, falling in the range of 0.09–0.12 [121]. It shows that common perceptions regarding the extent of cost savings resulting from LOS reductions have been substantially overestimated.

Another erroneous assumption is that the cost per day is the average for the entire stay [122,123,124]. The daily cost typically shows a logarithmic decay with time [123]. The cost for the first day of admission depends greatly on surgical versus medical care. For example, percutaneous transluminal coronary angioplasty (PTCA) has 66% of total costs on day 1, while heart failure has 27% on the first day [122]. A study relating to CCU costs showed that the first day accounted for 67% of total direct costs. Daily costs had declined to 40% of the average on the fifth day [124].

One study demonstrated that the last full day of hospital stay for 12 365 patients with a stay of 4 or more days accounted for only 2.4% of total costs, 6.8% for a 4-day stay [118]. For patients without a major operation, the last day accounted for 3.4% of costs [118]. The authors stated that “physicians and administrators must deemphasize LOS and focus instead on process changes that better use capacity and alter care delivery during the early stages of admission, when resource consumption is most intense”. It is unsurprising that reducing LOS does not yield the anticipated benefits promised by simplistic cost assumptions. Part of the problem was that for the trauma centre, only 42% of the costs were directly associated with care, the other 58% being indirect hospital overheads [118].

An additional fallacy is to assume that fixed costs are variable. A US study calculated that 84% of hospital costs were fixed. Of these, 32% were for support functions such as utilities, employee benefits, and housekeeping salaries, while 52% included direct costs of salary for service centre personnel [125].

It is sometimes forgotten that to reduce LOS involves more intense input into the earlier days of the stay, which can counterbalance cost savings from decreasing the later, less-intensive days [126]. Hirani et al. [127] suggest that the cost of follow-up care may sometimes unexpectedly increase or have been omitted from the cost calculations. Indeed, identifying a problem while mother and baby are still in the maternity ward can avert a more serious readmission from the community [3].

Bowers et al. [128] point out several issues, namely, that you cannot reduce staffing in proportion to a reduction in avLOS, that reducing avLOS increases the intensity of nursing care per patient, and that community resources have to be expanded to cope. The net cost saving is uncertain.

The study of Baretta [129] recommended that ‘indirect’ costs should be excluded from benchmarking studies because their inclusion led to serious errors in the determination of cost ‘efficiency. This issue is covered in the next section.

To conclude this section, this does not mean that reducing LOS via new technology/medications or optimum care pathways is not helpful. As Taheri et al. observed [118], it is far better to focus elsewhere and this will include issues around how to avoid clinically unnecessary admission and/or intervention [130,131,132,133,134,135], or how best to provide non-admission-based care which saves total system costs but reduces hospital income [135]. Optimal care results in a shorter hospital stay [136], i.e., focus on the clinical needs of the patient and shorter LOS will naturally ensue without trying to force the issue. This includes eliminating hospital-acquired infection, postoperative complications, need for critical care, medication errors and interactions, etc. [122].

#### 4.8.1. The Fixed (Indirect) Costs Dilemma

The greatest fallacy around LOS lies in ‘the fixed costs dilemma’ [3], where up to 60% of the cost of admission arises from the fixed (sometimes called indirect) costs of shared hospital supporting departments such as the hospital board of directors, finance, human resources, press and PR, etc., and depreciation on capital assets (buildings and equipment), etc. Note that the figure of 60% applies to the USA, where private healthcare imposes high transaction costs on hospitals such as excessive documentation of every cost item, individual invoices for every patient, offering payment plans to those who are uninsured, debt collection, etc. This proportion will be lower in other countries. Such fixed costs do not go away but can be partly mitigated by economy of scale for the non-patient-facing departments [137].

The fallacy lies in how these costs are shared (apportioned) with the total cost assigned to the patients. The moment that these fixed costs are apportioned based on LOS, then LOS suddenly becomes ‘expensive’. The correct way to share most fixed costs is based on admissions rather than LOS. This is a logical basis since the first day of the stay is the most expensive (as shown above) and the administrative costs occur at admission/discharge [118,122,123,124]. As a simple example, a hospital has $10 million in fixed costs with 10,000 admissions and 50,000 bed days. Apportionment based on admissions adds $1000 to each patient while that based on LOS adds $200 per bed day. If LOS is reduced by 1 day per patient, the fixed cost per day simply rises to $250 per bed day. As soon as the shared overhead costs are allocated by admission it becomes far clearer that the route to reducing costs may have more to do with administrative costs and the cost of capital (buildings, etc.), which becomes excessive in small hospitals, than in the direct medical costs [125,131].

Similar logic applies to ED and outpatient attendance, namely, allocate most of the fixed costs per attendance and not based on the length of the consultation, although with additional modification for complexity where time implies higher staff costs.

In addition, every department has largely semi-fixed costs for staffing, which stays roughly the same independent of how admissions may fluctuate [118,119,138,139]. Hence, if admissions are 15% lower or higher in one year, frantically seeking to reduce LOS will have virtually zero effect on total staff costs or upon the fact that the real problem is that income is variable.

Regarding fixed costs for staffing, studies in England have shown that the average nursing cost per occupied bed day reached a minimum around 35 average occupied beds and was 3-times higher at 10 average occupied beds. The relationship was exponential with size [138]. Another study showed that a 5-bed unit had 4-times higher costs per bed than a 35-bed unit. Costs rapidly escalated exponentially below 15 beds [139]. These results confirm the strong diseconomy of scale noted in Table 1 (from Supplementary Material S2) and in Figure 17.

My own research published in 2013 used NHS reference costs submitted by each hospital every year to show that the costs of the two HRGs covering normal and assisted deliveries followed the same cost curve relating to unit size as that from Erlang B in Supplementary Material S2, see L.21 in Supplementary Material S1. This was at a time when the English Department of Health was publicly insisting that economy of scale did not apply to HRGs.

The previous study on maternity capacity highlighted that dubious cost assumptions are often involved in justifying shifting postnatal work into the community [3]. The maternity/paediatric department must question any source of advice claiming that reducing LOS will make substantial (total cost) savings.

Less well recognized is the fact that the overall apportionment process is highly unreliable when independently applied across multiple organizations. NHS reference costs include apportioned capital, direct and overhead costs. The 2013 study of maternity costs in England looked at the 2009/10 submitted reference cost for every maternity inpatient and outpatient HRG at every hospital in England. Irrespective of the number of inpatient/outpatient admissions/attendance, the bulk of submitted costs lay in the range of 0.5- to 2-times the national average for each HRG. The full range lay between 0.2-times to 5-times the national average, i.e., the submitted data defy the normal behaviour expected from simple sampling uncertainty [21]. A calculation of the standard error of the average would suggest that there was limited confidence in the national average, see L.21 in Supplementary Material S1.

It should come as no surprise that the calculated national average cost for each HRG is subject to extreme year-to-year variation, see O.17 in Supplementary Material S1. The idea that a particular HRG actually costs, say £1245.56, is shrouded in uncertainty and greatly depends on apportionment decisions which increasingly fail even for direct costs allocated to a single HRG/DRG. HRG/DRG tariffs have the illusion of accuracy and fairness.

An extreme example of the overhead cost dilemma would be the choice of where to locate a rehabilitation unit. As soon as the unit is located within an acute hospital, it immediately attracts a share of all overhead costs, CEO, Chairman, Directors, Finance, Personnel, etc. [3]. Recall that all acute hospitals have substantial financial and regulatory responsibilities. Given the relatively long avLOS for rehabilitation, apportionment based on LOS then attracts even more overhead costs and the unit has the illusionary appearance of being extremely expensive. The apportionment of fixed/overhead costs can lead to flawed decision making.

As recommended in the maternity study [3], staff should discuss with their finance department issues around fixed costs, variable costs, step costs, marginal cost, average cost, and total cost, and the risks implied by payment/financing systems, e.g., DRG/HRG, capitation funding and institutional variations.

#### 4.8.2. Economy of Scale and the Cost per Patient

Economy of scale (EOS) is a well-recognized concept which has a defining impact upon the cost per patient [86]. However, the question remains as to why English hospitals and departments are generally larger than the supposed optimum size and English maternity units are far bigger than the optimum size proposed for Belgium [38,39]. The review of Giancotti et al. [140] identified a wide variety of methods, each with different hidden assumptions and with models blindly applied to whole hospitals. Perhaps the greatest criticism is that all studies have no common theoretical basis and hence, there is no way to sense-check their outputs. Most importantly, all have ignored the defining impact of the Erlang equations on the unavoidable effect of size on individual department costs. Hence, the key issue is not around size per se but the size of the individual bed pools, which was identified in a study published in 2012, see L.12 in Supplementary Material S1, and then applied to costs in maternity units, see L.21 in Supplementary Material S1. More recently, it has been used to explain why different countries have different average bed occupancy rates [2].

Economy of scale is thus a function of the weighted average of the size of the individual units/departments and their bed occupancy rates [2], see L.12 in Supplementary Material S1. Unsurprisingly, the most recent research has swung toward the defining effect of department size on costs [119,141,142], although seemingly without realization that the Erlang equations provide the necessary theoretical framework.

The births-to-beds calculator in Supplementary Material S2 clearly shows that the optimum average bed occupancy rate increases with size up to 1000 beds; however, the greatest increase in average occupancy (and hence lower costs) occurs up to 100 beds, with crippling costs >6-times higher below 5 beds, as discussed earlier [138,139].

Freeman et al. observe that EOS is stronger for unscheduled care [119]. A 1993 study by Perkins [13] details research on EOS in obstetric care going back to the 1930s. EOS was shown to be highly relevant to the Belgian system of obstetric care via a multitude of small hospitals [38,39]. A minimum economic EOS in obstetric care was observed at 557 birth p.a. with increasing EOS up to at least 900 births p.a. [39,40]. Seemingly little has changed since the 1930s [13].

It should come as no surprise to find that US hospitals which invest heavily in capital (buildings/equipment) to increase market share end up with inflated costs arising from the higher costs of capital depreciation and running costs per patient [143]. This issue was identified earlier for MRI scans in the USA [61,62].

Despite the above limitations, at the whole-hospital level, EOS studies show that below 200–300 beds (equivalent to less than 13–19 paediatric or 8–11 maternity beds) there is diseconomy of scale, and the total cost per patient only shows economy of scale above this size and reverts back to diseconomy of scale above 600 beds (equivalent to 39 paediatric or 23 maternity beds) [86]. The increase above 600 beds probably arises from the fact that larger hospitals can offer increasing specialization and treat the most complex cases. As a reference point, in England, maternity comprises around 7% of acute hospital beds [27]. This ratio implies an optimum size of 32 maternity beds and an economic minimum size of 14 beds.

In Belgium, where the minimum economic size for a maternity unit was evaluated to be 557 deliveries per year [39,40], it was noted that 17 small units (15% of units) could be closed while still maintaining a 30 min drive time [39]. Inspection of the data associated with this study indicates that minimum cost occurs above 1000 births. Given the small size of even the largest Belgian hospitals, the slight diseconomy of scale probably reflects the fact that the larger units are more ‘tertiary’ and will receive more complex cases. Note that in Belgium, the three largest maternity units (probably with a tertiary focus) were only slightly higher than 3000 births (and could easily go into the 2–3000 births band), with the 4–5000 band for births being the most common size in England (from Figure 4). A similar situation also appears to apply in Denmark [144], which has a WPD of 2938 per km^2^, which is similar to Belgium [64]. As mentioned earlier, 557 deliveries per annum probably refers to a midwife-led unit rather than an obstetric unit.

In the USA, 557 deliveries would exclude 35% of small hospitals with more than 25 births per annum, see Supplementary Material S4 [25]; however, drive times remain an issue since only 61% of the population are within 30 min of obstetric care, while only 33% have 30 min access to level 3 neonatal care [145]. By way of contrast, in England, 79% of mothers live within a 30 min drive time and 99% are within 60 min of the nearest ED and only 8% have no obstetric unit within 30 min [68].

The issue here is that small units are unable to reduce their staffing costs because there is a minimum staffing level required simply to run a ward/unit [138,139]. This is a fixed cost set against a highly variable daily number of admissions (as per Section 4.4) and bed occupancy. Attempting to reduce avLOS to reduce costs is futile, although still encouraged provided it benefits the patient.

#### 4.8.3. HRG/DRG Tariffs, Fair Costs and Long-Stay Patients

While the reality exists that most hospital costs behave as if they were fixed [124], uninsured patients, insurance companies, and governments all demand that they be presented with a ‘fair’ price. Most of these ‘purchasers’ believe that staying longer costs more—hence, the push to reduce LOS. While I have indicated that many costs can be apportioned based on a count of admissions, a more nuanced approach is required.

Length of stay could be argued as being related to certain direct costs such as catering, laundry of linen, consumables, and the depreciation costs associated with the bed, ward equipment and floorspace. These will not account for a large cost per day of stay. Recall that costs decline in an approximate exponential decay as time since admission increases [123].

Staff costs become more problematic and should probably be split between the counts of admissions and total bed days. However, recall that occupied bed days depends on admission and admissions are subject to both Poisson and environmental variation. You only know the total at the end of each financial year, which suggests that you need to estimate the minimum number of admissions and occupied bed days for each year. To assume an average is to invite financial disaster.

In 2004, I conducted a large Monte Carlo simulation to see how income would vary based only upon Poisson variation associated with an assumed average number of elective admissions into each of the available elective HRG/DRGs with their unique prices for payment to the hospital [146]. This was for a medium/large English hospital with 670 beds (large/very large by US standards), with an expected 31 200 elective admissions at a present-day elective income around £63 million (multiply this by 2 to get a $US equivalent). This best-case simulation gave around a £5 million range in income (£60.4 to £65.2 million) and a 1500 range in elective admissions (30,400 to 31,900). In the real-world, elective admissions have 2-times higher variation than Poisson statistics, while emergency admissions are 3-times higher than Poisson. Hence, the range in income doubles from £5 million to £10 million, etc. Clearly, the inclusion of emergency admissions would give a greater range in income and admissions. As an aside, note that the probability distribution for income versus admissions is shaped like a tilted ellipse with sectors like low admissions/low income having a higher probability than low admissions/high income, etc.

The dilemma is obvious, with largely fixed costs having to be met by variable income, and actual costs needing to be allocated against variable admissions and case mix, while simultaneously needing to deliver the surplus/profit required for the purchase of new equipment and other capital investment. That, which may at first seem simple, is profoundly complex, and made more complex when the HRG/DRG tariff makes no allowance for economy of scale.

It is suggested that all small departments have a discussion with their finance department around the proportion of their price which is due to overhead costs and the alternative ways in which prices could be calculated, compared to what purchasers are willing to pay—all part of any decision regarding new investment to cope with rising demand or disinvestment.

The issues around a ‘small’ department discussed in Section 4.5 are equally applicable to referral from primary to secondary care and to attendance at the ED. Seasonality and Poisson randomness continue to apply and create financial risk to both the purchaser and provider alike within HRG/DRG-based systems, see B.1–B.11, C.1–C22, and D.1–D.12.

Many will not be aware that HRG/DRG tariff prices contain multiple hidden assumptions regarding how costs behave, see O.1–O.21. The price of an English HRG calculated using reference costs submitted by NHS Trusts each year shows considerable variation from one year to the next, see O.17 and O.19 in Supplementary Material S1.

The formulae used to calculate how much government funding should be paid to each state/region/purchaser (capitation funding) may also contain hidden assumptions, see P.1–P.8. All fee-for-service systems have hidden assumptions which inadvertently place greater financial risk on some and not others. The aim of government policy should be to minimize such risks.

### 4.9. International Benchmarking Using Ratios from the English NHS

Supplementary Material S4 gave a large table which used US data compared against English benchmarks. The English NHS represents a good benchmark since it offers universal healthcare, which is free of charge. However, English benchmarks need to be understood in the context that on a like-for-like basis, it has already been established that Australia has higher expressed bed demand (as occupied beds) than England [2], while the USA has substantially lower expressed bed demand. This is largely due to the fact that health insurers in the USA use every means possible to limit utilization, i.e., exclusions, copayments, etc., and even with Obama Care, utilization among the disadvantaged remains low. As one example, the avLOS in American states is correlated with average state income, see K.3 in Supplementary Material S1.

Supplementary Material S4 is also used to illustrate the role of WPD at the state level and the very high number of counties which have insufficient bed demand to support even the smallest viable hospital.

Section 3.5 and Figure A4 in Appendix A provided evidence that some countries have chosen to operate with small hospitals relative to their WPD. A recent study in Germany sought to explain the historic issues behind why this country had so many small hospitals [72]. However, WPD only explains a small part of why care is so expensive in the USA. Section 3.5 gives a number of hints as to this gap. As a benchmark, the average cost per birth is 2.8-times higher in the USA than in England [74,75]. Firstly, utilization rates for MRI scanners are 3-times lower in the USA than England, implying that the capital and workforce cost per scan should be 3-times higher. Examples were given of American counties with >3-times the number of hospitals that would occur in England [61,62]. This disparity occurs because the USA healthcare market is based on a misplaced belief that free markets are the most efficient. While this may be true for consumer goods, healthcare is not a consumer good. Consumerism is evident in the number of MRI scanners per head of population and in the proliferation of small (costly) hospitals as hospital chains compete for market share—a supposed competitive advantage in a free market.

Given the limited access to maternity and paediatric care in many areas of the USA [69,70], it would theoretically be desirable for the federal government to intervene [4].

However, substitute local data for your own country into S4 and compare the situation to England. Additionally, use the approach in Figure A4 to see the extent to which WPD is acting to determine the unavoidable effects of hospital size upon costs.

### 4.10. Is Deprivation the Main Driver of Obstetric/Pediatric Excess Bed Demand?

Deprivation is commonly used as an explanatory variable for excess admissions, and bed demand. However, my own research shows that social groups rather than deprivation give far greater predictive power, see B.11 in Supplementary Material S1. Social groups reflect health behaviours, while deprivation does not primarily do this [147]. Social groups are usually constructed using the same methods as for constructing consumer groups. Social groups are also likely to reflect case mix complexity. The maternity study [3] demonstrated how the trends in births in England profoundly depend on social group. Social groups are highly likely to reflect population density and will also reflect risk factors such as obesity, etc. [147].

A study regarding demand for ambulance services among children and the elderly demonstrated, in the 27 ambulance service areas in England, that population density, not deprivation per se, was the primary determinant for the call-out rate. Deprivation showed a very weak linear relationship, while population density had a strong non-linear effect with a sharp decline in call-out rates below 1000 persons/km^2^ (2600 persons per square mile) dropping to near zero rates in the lowest population density areas [148].

It should be noted that WPD is a primary factor in infectious disease transmission [94,95,96,97]. Far greater research is required on this topic to disentangle the effects of distance, WPD and measures of deprivation upon the expressed demand in hospitals and departments.

A recent study suggested that age standardized mortality rate (ASMR) in children may be an additional relevant factor [2,4]. On this occasion, ASMR is an alternate measure of the experienced ‘deprivation’ in relation to health. Further research is required on the social and other factors influencing bed demand in both obstetrics, paediatrics and other departments.

### 4.11. Year of Birth Cohorts and the Forecasting Spreadsheet

It is almost an industry standard to use 5-year age bands in capacity planning. No one questions this but simply follows the crowd. The cyclic behaviour of births seen in many countries shown in Figure 5 and Figure 6 implies that every 5-year age band will be subject to waves of births passing through the age band, which will interfere with the outputs based on this method.

The forecasting spreadsheet in Supplementary Materials S6 invites the reader to follow the admissions/occupied bed days for year-of-birth cohorts up to 19 years after birth. This approach seeks to direct analysis away from simplistic use of wider age bands in capacity planning. This is reinforced by giving typical 5-year age bands for paediatric admission rates in England (2012/13 to 2024/25) in Supplementary Material S6, which are then interpolated to give a single-year-of-age profile for admission rates relative to age 0. The key observation is that ages 0 and 1 dominate the admission rates, which explains why using births as a proxy works so well. See the tab ‘Admission rate by age’ in Supplementary Material S6.

Each birth cohort lies in a diagonal across the spreadsheet. This is based on the recognition that birth cohorts can have lifelong patterns of health. For example, birth during different parts of the solar cycle seem to influence longevity and disposition to certain physical and mental conditions [149]. Exposure to antibiotics and pathogens during prenatal and early life can influence health trajectories across the lifespan [150], and H1 influenza-exposed cohorts look to have worse health outcomes than H3-exposed cohorts [151].

Outside of the first 19 years of life, the WW II baby boom in England led to 1.8 million births between May 1946 to May 1948 which was 50% higher than the point of minimum births between November 1939 and November 1941. In 2025, this birth cohort was aged 79 to 81 years and represents a sudden onset of increased healthcare demand which moves forward in time. Indeed, this cohort began to appreciably affect inpatient demand around 2011 when they were 71 to 73 years old and began to die in appreciable numbers.

These combined factors are called age-period-cohort (APC) effects and influence the health response to the environment experienced in the current year. They are difficult to disentangle [152] but nevertheless are another source of variability/uncertainty regarding each year’s level of admissions and bed demand. Birth cohorts remain an area of international interest [153] and form part of the evolving patterns of volatile bed demand.

The forecasting spreadsheet S6 simply gives the opportunity to see the outworking of the APC effects using larger national and regional data and reinforces the futility of using averages in demand/bed capacity forecasting.

All APC models appear to focus on the babies and their life course, and I am not aware of any APC studies investigating the APC of the mothers and the complications associated with pregnancy and the effect on the babies at birth.

## 5. Why Is Variation Between Maternity Units So High?

Two reports in England noted large variation between maternity units in terms of occupied bed numbers, staff and equipment [67,68]. This is exemplified in the material given in Supplementary Material S3. This is not unique to England and a study in Belgium also noted wide variation in bed occupancy between units with the same number of beds [37]. Figure A1, Figure A2 and Figure A3 (Appendix A) all exhibit the same extreme variation in average bed occupancy for units with the same number of beds.

The proposed explanation for this extreme variation is that there is widespread ignorance among Health Departments and hospital managers regarding how to interpret the trends in births and how to convert births to beds [3].

The aim of the capacity planning series is to give some uniformity to international hospital capacity planning. While all parts of the series [1,2,3,4,5] imply a degree of flexibility is required, especially with regard to surge capacity during epidemics and pandemics, there is no point in carrying excess beds when there is little possibility of these beds ever being used. The space should be put to better use.

## 6. Limitations of the Study

Births are used as the closest approximation to women falling pregnant because data on births is widely available.

Note that annual births, monthly seasonality and bed days per birth are all subject to socio-economic, statistical, environmental and infectious forces, hence the need to operate with a slight excess of beds. This excess can be investigated using the bed calculators provided in Supplementary Material S2.

Hence, the study recommends that capacity planning leaves room for flexibility in the building design (as flexible floor space) to cope with unexpected changes in demand. All manners of financial, staffing and other issues will impact this imprecise ‘risk’ evaluation.

## 7. Key Recommendations

Several key recommendations arise from this study.

1. Although the USA and UK have some of the most extreme examples of cyclic birth trends, this does not imply that all areas within these countries will follow the same patterns [3]. Health departments should insist that statistical agencies prepare a wider range of birth forecasts, which can include those based on TFR, three-parameter models, and other pragmatic local approaches detailed in this and the previous study [3]. They must ensure that the potential range of births is communicated to all regional health authorities and hospitals. Hospitals should have contingency plans to deal with anticipated periods of higher births [3] and deal with surges in demand from seasonality.
2. The ideal position is that pregnancy, childbirth, neonatal and paediatric care be free of charge and funded from hypothecated state general taxation. For-profit health insurance with its inherent high transaction costs, and temptation to maximize profits, is incompatible with care delivered to those who are unable, by virtue of childhood, to earn money. The USA appears to exemplify this requirement with disturbingly poor childhood mortality across all age bands [4].
3. It must be clearly understood that small maternity/neonatal/paediatric will suffer from unavoidable high capital and staff costs per admission and that these costs will be further distorted by the allocation of shared overhead costs as was discussed previously [3].
4. Health Departments may need to operate maternity/paediatric units in remote locations where rationalization is not possible.
5. High inpatient occupancy (and related turn-away) is known to be associated with delays to admission and poor patient outcomes such as hospital-acquired infection rates [2,62,106]. Such studies are usually conducted at large units where bed occupancy is used as an (incorrect) proxy for turn-away—although the bed occupancy rate is also a proxy measure for busyness. An upper limit on turn-away should be stipulated. Given the fact that many units operate at an annual or quarterly turn-away less than 0.1%, it is suggested that no unit should operate at >5% turn-away in the worst month.
6. Bed demand is highly seasonal with occasional high years. The bed planning calculation is therefore one regarding available floor space rather than a fixed number of beds. The floor space simply provides the opportunity to flex the number of available beds. Such flexibility is profoundly important for staffing, which dictates against small units. It is recognized that units situated in small towns and remote areas will struggle to implement such flexibility unless on-call staff can be redeployed from elsewhere.
7. The inherent volatility in neonatal bed demand implies that the actual trends in occupied beds become the benchmark rather than futile attempts to separately forecast admissions and LOS—which are both part of the inherent volatility.

## 8. Research Topics

This study highlights several key areas for future research.

1. The evidence seems to show that small units are structurally expensive simply due to the high Poisson randomness in arrivals. More studies are needed in this area as somewhere around >30 beds could be the optimum size.
2. International studies are required regarding costs in small units (maternity, paediatric, etc.) with participants selected across a range of countries with disparate WPD.
3. Is neonatal mortality highest in countries with the highest proportions of small units, i.e., with less than 10 beds?
4. Are units with high turn-away associated with low avLOS, i.e., has low avLOS become an indicator of poor planning rather than an efficient unit?
5. The interaction between turn-away and staffing on adverse outcomes is an area in urgent need of investigation. This interaction is likely to depend on the size of the unit. Recognition needs to be given to the fact that high levels of transfers out of small hospitals will simply shift the poor outcomes to the receiving hospital.
6. International data on total curative beds need to be segmented to include data on paediatric, maternity and mental health beds and occupancy. Measures of the average number of available and occupied beds are required for each country.
7. A specific study is required regarding international levels of maternity and paediatric available and occupied beds.
8. A simple tool (as in Supplementary Material S2) should be freely available to allow hospitals to calculate their annual average and monthly levels of turn-away. The inputs are average beds available and average occupancy for any period. Ideally, this could be via the WHO, World Bank, or government health department websites.
9. While pathogen interference has been mostly researched for adults, additional research is required specific to neonates and children and how the pathogen mix varies from year to year. Common persistent pathogens need to be considered as contributory factors.
10. A review is required regarding the approaches in different countries to the issue of paediatric, maternity and other inpatient care for rural and remote populations. This will be influenced by relative wealth and how care is funded. See #11 below.
11. Specific research is required regarding how care is funded relating to distance, unit size, and cross-border flows in federal countries.
12. A study is required to document capital, staff and overhead costs per patient relating to unit size and how to calculate equitable adjustment factors to underpin HRG/DRG payment systems.
13. The factors regulating expressed demand require urgent research and WPD, distance, ASMR, deprivation and/or social group need to be disentangled.

## 9. Policy, Funding and Operational Implications

Around the world, the issue of ‘medical deserts’ is very common [71]. It may be a surprise to note that even in England there are eight small/remote hospital sites with cost uplifts due to small size ranging from +8.6% at the St Mary’s hospital (Isle of Wight, population 142,000) to +4.7% at the Cumberland Infirmary (Carlisle, Cumbria, population 75,000 in the town plus surrounding countryside) [141,142]. Remote was defined as having <200,000 population within 1 h road travel, and the next nearest ED being >1 h away by normal road travel time [143].

From Supplementary Material S4, we see that in the USA, a cutoff point of 200,000 population encompasses 89% of counties and would imply a single hospital with a maximum size of 470 beds. By comparison, Bay County (Florida) with a population near 200,000 is served by three major acute hospitals, implying greatly higher costs due to poor economy of scale. Two of these hospitals offer specialist/tertiary level care [154]. It is this type of excessive provision which explains why the average size of an acute hospital in the USA is only 190 beds (but less than half this number in the Midwest states), and 60% have <100 beds [155].

In the USA, hospitals that closed obstetric services reported that physician shortages (67%), financial losses (62%), clinical safety (56%), liability insurance costs (51%), and nurse shortages (39%) influenced the decision to close the unit. Among hospitals with obstetrics, more than half (55%) reported that their hospital was operating with a profit margin, but only 41% said their obstetric unit had more revenue than costs. Of the hospitals with obstetrics who responded about the future of their obstetric units, 77% were confident that they would continue providing obstetric care in 10 years; their open-ended responses highlighted the importance of hospital leadership’s commitment to maintaining obstetric services in their communities [156].

The cost of births at very small hospitals is problematic. Some staff will need basic training in delivering a baby and litigation insurance, which becomes part of the fixed costs. In New Zealand, the smallest midwife-led unit has four beds and births can occur elsewhere. The ratio of beds per birth is 10.4 and 11.2 in the two largest hospitals [33,34]. At one birth, this ratio lies somewhere between 10,000 and 1000 beds per birth, 10 births between 300 and 1200 and at 100 births between 70 and 150. These ratios reflect fixed capital costs and seemingly also direct costs. In the USA, births are occurring at the ED in those areas without a maternity/midwife unit [69]. This would not be problematic where there is no purchaser/provider split, as in New Zealand; however, it implies that the real cost of such births is probably very high.

The effect of relative wealth on hospital bed provision was addressed in parts 1 and 2 of this series [1,2]. Maternity costs in the USA are a structural issue. In 2000, some 61% of maternity units handled fewer than 1000 births per annum, which drove closures due to cost pressures, leading to 55% of units below 1000 births in 2019 [25]. These units, mostly servicing rural/remote populations, cannot reduce their costs and a national strategy is required for optimal placement which transcends state and county borders [3].

It could also be argued that the number of units permitted in medium/large towns and cities be limited to ensure lower costs through economy of scale.

In terms of safety, small rural units have problems recruiting staff and maintaining adequate staff to patient/birth ratios during both the seasonal and the frequent times of Poisson-induced higher-than-average demand [68,69,157,158,159].

The most relevant policy implication is the funding of maternity and paediatric services via hypothecated taxation and direct payment to providers. This minimizes the futile cost burden of private health insurance and claim processing. The next is around state supervision of capacity planning to ensure equitable distribution of resources and that hospitals operate at acceptable levels of average occupancy, turn-away and safety.

It may come as no surprise that in the UK, the governments of Northern Ireland, Scotland and Wales have all abandoned the purchaser/provider split and the HRG tariff. This is a pragmatic response to the fact that hospital costs are largely fixed irrespective of the (fluctuating) activity and case mix, and that the HRG tariff only amplifies the risk to purchaser and provider alike.

The reader is recommended to explore the issues around the limitations of HRG/DRG tariffs in O.1–O.21, financial risk in healthcare in N.1–N.39, and issues around funding formulae for capitation funding in P.1–P.8.

The US journal ‘Health Affairs’ documents the struggles to contain costs in that country. I would argue that these are largely directed at the margins rather than the core structural issues.

## 10. Conclusions

This study should be read in conjunction with the associated maternity [3] and paediatric [4] capacity planning studies. The use of queuing theory and the Erlang B equation for maternity unit capacity planning was first advocated in 1959 [8]; however, there appears to be widespread ignorance to its importance. Two bed calculators are provided in the Supplementary Material S2, which can be used for obstetric, maternity, midwife-led, birthing wards, obstetric theatre, neonatal and paediatric unit bed capacity. It can equally be applied to other smaller departments.

Tables/calculations can be easily generated linking births and average total bed days per birth to the required number of available beds. These calculators enable the rapid testing of multiple what-if scenarios with the first calculator allowing for the input of seasonality and day of week effects to give the maximum required beds in the highest birth month and for the impact of ‘elective’ C-section and other interventions on the weekday profile. Larger units benefit from considerable economies of scale in both throughput per bed and cost per patient.

The lines of turn-away from Erlang B are an excellent way to compare functionally similar units but with different sizes. The lines act as a framework to question why some units are operating at higher turn-away. While Monte Carlo simulation can be used to calculate bed capacity, the lines of turn-away will give similar answers but far more quickly.

The high WPD in England explains why the average cost per birth is low compared to other countries. WPD in the majority of world countries is low and this implies that small hospitals/units and medical deserts are a common issue outside of the few large cities. This is exemplified in Belgium, where 81% of maternity units in Brusells have >25 beds while 60% of units outside Brussels have <25 beds [37].

The reality of economy of scale on the unavoidable higher costs in smaller units implies that any HRG/DRG tariffs must have a mechanism to avoid underfunding such units. However, such units are often forced to operate at high occupancy and turn-away to remain financially competitive with larger units irrespective of the deleterious effects. This is especially the case since countries have different maternity unit size distributions depending on rural/remote population characteristics and on historic factors. In Belgium, the minimum size for a viable maternity unit has been estimated at 557 births per annum [39,40], which presents problems in countries with high proportions of rural/remote populations. The figure of 557 births may well be too low because the Walloon and Flanders regions of Belgium only have small hospitals and 557 births implies a midwife-led rather than an obstetric unit [37,38,39]. This disparity also exists when there is a very large gap between the smallest and largest units. Health departments seem to have forgotten the reality of this link. It is exceedingly unwise to pay all maternity/paediatric units using the same HRG/DRG tariff and not expect to inflict significant financial and safety issues upon smaller units.

The LOS for common birth events varies greatly between countries [66], although further studies are required regarding the link between LOS and patient/baby outcomes.

Forecasting future births is highly dependent on local factors including construction of new dwellings and the availability of employment suited to young families. This area requires greater input from health departments and statistical agencies.

Neonatal bed requirements are shown to be complex, which implies that units should be built with excess floor space to allow for unexpected/unplanned future trends.

Universal funding for maternity/paediatric care is the recommended standard; however, hybrid options do exist such as the American state of New York’s ‘Basic Health Program’ [160].

## Figures and Tables

**Table 1:** The link between births per annum, bed days per birth and required (available) beds at 0.1% turn-away for various levels of total bed days per birth in the maternity unit (antenatal + postnatal), illustrated up to 15 beds in the absence of seasonality.

Available Beds	3.2 Bed Days/Birth		4 Bed Days/Birth		5 Bed Days/Birth	
	Annual Births	Beds/1000 Births	Annual Births	Beds/1000 Births	Annual Births	Beds/1000 Births
1	1	8761	1	10,951	1	13,689
2	6	350	5	438	4	548
3	22	138	17	173	14	216
4	50	79.6	40	99.6	32	124.4
5	87	57.6	69	72	56	90.1
6	131	45.7	105	57.1	84	71.4
7	180	38.8	144	48.5	115	60.6
8	234	34.2	187	42.7	150	53.4
9	292	30.8	234	38.5	187	48.1
10	353	28.4	282	35.4	226	44.3
11	417	26.4	333	33	267	41.3
12	483	24.9	386	31.1	309	38.8
13	551	23.6	441	29.5	353	36.8
14	622	22.5	498	28.1	398	35.2
15	694	21.6	555	27	444	33.8

**Table 2:** Estimated additional cost per birth relative to England based on the size distribution of maternity units. Size weights are by 1000 birth increments and are given in Section 2.3.7.

Country	Estimated Additional Cost
England	0%
Northern Ireland	9%
Scotland	9%
Wales	10%
Australia	15%
Sweden	15%
New Zealand	19%
Belgium	42%
Switzerland	46%
USA	52%

## Data Availability

Most data are publicly available, and the source is given in the study. Any other data or supporting spreadsheet can be obtained from the author on request.

## Supplementary Materials

The following supporting information can be downloaded at https://www.mdpi.com/article/10.3390/ijerph23060711/s1, Document S1: Relevant publications. Spreadsheet S2: Erlang B-based bed number calculators. Spreadsheet S3: The 2013 England capacity shock. Spreadsheet S4: US states and counties. Spreadsheet S5: A birth forecasting tool with worked examples. Spreadsheet S6: Birth time cascades. Table S1: Measures of seasonality in European countries.

## Appendix A

**Table A1:** Weighted population density (WPD) for selected larger urban areas and some low WPD rural areas in England and Wales. The four highest/lowest WPD London Boroughs are included. WPD is calculated using lower super output areas (LSOA) which average 5 km^2^ and mostly have 1000 to 3000 population. Wales (W); London (L). Calculated from [50]. London, England and Wales in bold to allow for easy identification.

Area	WPD	Area	WPD
City of London (L)	20,573	Sandwell	5504
Tower Hamlets (L)	18,958	Hillingdon (L)	5494
Westminster (L)	16,941	Plymouth	5442
Hackney (L)	16,810	Oxford	5388
**London**	**10,129**	Bradford	5278
Portsmouth	9436	Sheffield	5239
Brighton and Hove	9150	Newcastle upon Tyne	5086
Leicester	8914	Leeds	4870
Manchester	8303	Bromley (L)	4812
Luton	7957	**England**	**4788**
Slough	7297	York	3832
Southampton	7190	Milton Keynes	3437
Liverpool	7142	Swansea (W)	3293
Watford	7124	**Wales**	**2434**
Bristol	7091	Herefordshire	1635
Reading	7048	Cornwall	1613
Nottingham	6961	Mid Devon	1296
Birmingham	6762	West Devon	905
Cardiff (W)	6456	North Norfolk	790
Richmond upon Thames (L)	6228	Gwynedd (W)	760
Coventry	6027	Rutland	714
Bexley (L)	5836	Ceredigion (W)	691
Kingston upon Hull	5689	Derbyshire Dales	628
Blackpool	5649	Powys (W)	347
