# Supplementary material for: Capacity Planning for Small Hospitals and Departments Illustrated Using Maternity and Paediatrics Departments: Roles for Weighted Population Density, Seasonality and Size, Myths Around Length of Stay and Factors Influencing Costs and Funding
Source: Int J Environ Res Public Health. 2026 May 27;23(6):711. doi: 10.3390/ijerph23060711 (PMC13299548; doi:10.3390/ijerph23060711)
Supplement: Supplementary file 1 [file ijerph-23-00711-s001.zip › Supplementary Materials S1.pdf]

Supplementary Material S1. Relevant publications by the author

The following is a list of publications arising from 30 years of research into the areas of forecasting healthcare demand, hospital bed modelling and the associated areas of healthcare costs and cost fluctuations (financial risk), all of which are interlinked.

The papers are written in a non-academic style so that busy healthcare managers and policy makers can understand the issues. The fundamental insight comes from hard-won understanding by long experience and extensive supporting literature. To avoid self-citation, articles will be referred to as A.1., B.11., etc., in the text. Contact the author at hcaf_rod@yahoo.co.uk if you are unable to locate a full-text version of any paper.

BJHCM = British Journal of Healthcare Management. **BJMMR** is now J Adv Med Medical Res (**JAMMR)**, articles archived at https://www.journaljammr.com/index.php/JAMMR/issue/archive.

A. Understanding Emergency Admissions and Unscheduled Care

1. Jones, R. 1997. Emergency admissions: Admissions of difficulty ***Health Service Journal*** 107(5546), 28-31.
2. Jones, R. 2009. Trends in emergency admissions. ***BJHCM*** 15(4), 188-196.
3. Jones, R. 2009. Cycles in emergency admissions. ***BJHCM*** 15(5), 239-246.
4. Jones, R. 2010. Emergency preparedness. ***BJHCM*** 16(2), 94-95.
5. Jones, R. 2010. Gender ratio and hospital admissions. ***BJHCM*** 16(11), 541.
6. Jones, R. 2011. Cycles in gender-related costs for long-term conditions. ***BJHCM*** 17(3), 124-125.
7. Jones, R. 2012. Gender ratio and cycles in population health costs. ***BJHCM*** 18(3), 164-165.
8. Jones, R. 2012. Environment induced volatility and cycles in population health. Positive Health Online 194 (May), http://www.positivehealth.com/article/clinicalpractice/environment-induced-volatility-and-cycles-in-population-health
9. Jones, R. 2013. Is the demographic shift the real problem? ***BJHCM*** 19(10), 509-511.
10. Jones, R. 2013. Trends in elderly diagnoses: links with multi-morbidity. ***BJHCM*** 19(11), 553-558.
11. Jones, R. 2014. What is happening in unscheduled care? ***J Paramed Pract*** 5(2), 60-62.
12. Jones, R. 2014. Forecasting conundrum: a disease time cascade. ***BJHCM*** 20(2), 90-91.
13. Jones, R. 2014. Long-term cycles in admissions for neurological conditions. ***BJHCM*** 20(4), 192-193.
14. Jones, R. 2014. Trends in admission for allergy. ***BJHCM*** 20(7), 350-351.
15. Jones, R. 2015. Forecasting medical emergency admissions. ***BJHCM*** 21(2), 98-99.
16. Jones, R. 2015. Estimating acute costs. ***BJHCM*** 21(3), 152-153.
17. Jones, R. 2015. Understanding growth in emergency admissions. ***BJHCM*** 21(4), 195-197.
18. Jones, R. 2015. Exploring trends in demand for urgent care. ***J Paramed Pract*** 7(10), 486-488.
19. Jones, R. 2016. The unprecedented growth in medical admissions in the UK: the ageing population or a possible infectious/immune aetiology? ***Epidemiology: Open Access*** 6(1), 1000219. The Unprecedented Growth in Medical Admissions in the UK: The Ageing Population or a Possible Infectious/Immune Aetiology? (omicsonline.org)
20. Jones, R. 2016. Rising emergency admissions in the UK and the elephant in the room. ***Epidemiology: Open Access*** 6(4), 1000261 Rising Emergency Admissions in the UK and the Elephant in the Room (omicsonline.org)

B. Trends in Emergency Department Attendances and Subsequent Admission

1. Jones, R. 2010. Forecasting emergency department attendances. ***BJHCM*** 16(10), 495-496.
2. Jones, R. 2012. Ambulance callouts and disruptive technology. ***BJHCM*** 18(2), 112-113.
3. Jones, R. 2012. Age-related changes in A&E attendance. ***BJHCM*** 18(9), 502-503.
4. Jones, R. 2013. Trends in unscheduled care. ***BJHCM*** 19(6), 301-304.
5. Jones, R. 2013. Hidden complexity in A&E trends in England. ***BJHCM*** 19(7), 354-355.
6. Jones, R. 2013. A&E attendance: the tip of a wider trend. ***BJHCM*** 19(9), 458-459.
7. Jones, R. 2014. Untangling the A&E crisis. ***BJHCM*** 20(5), 246-247.
8. Jones, R. 2015. A&E tipping points. ***BJHCM*** 21(6), 248-249.
9. Jones, R. 2015. A&E admissions: where next? ***BJHCM*** 21(6), 292.
10. Beeknoo N, Jones, R. 2016. Factors influencing A&E attendance, admissions and waiting times at two London hospitals. ***JAMMR*** 17(10), 1-29. http://www.sciencedomain.org/abstract/16193
11. Beeknoo N, Jones, R. 2016. Using Social Groups to Locate Areas with High Emergency Department Attendance, Subsequent Inpatient Admission and Need for Critical Care. ***JAMMR*** 18(6), 1-23. http://www.sciencedomain.org/abstract/16693

C. Forecasting and Understanding Demand

1. Jones, R. 1996. Estimation of annual activity and the use of activity multipliers. ***Health Informatics*** 2, 71-77.
2. Jones, R. 1996. How many patients next year? Healthcare Analysis & Forecasting, Camberley, UK. (Book) ISBN (10) 190093101X
3. Jones, R. 2010. Forecasting year-end activity. ***BJHCM*** 16(7), 350-351.
4. Jones, R. 2010. Forecasting demand. ***BJHCM*** 16(8), 392-393.

5. Jones, R. 2011. Cycles in inpatient waiting time. ***BJHCM*** 17(2), 80-81.
6. Jones, R. 2011. Death and future healthcare expenditure. ***BJHCM*** 17(9), 436-437.
7. Jones, R. 2012. Weathering the storm: Birth forecasting in turbulent times. ***Midwives Magazine*** 15(2); https://www.rcm.org.uk/news-views-and-analysis/analysis/weathering-the-storm
8. Jones, R. 2014. Expected trends in births and deaths to 2037. ***BJHCM*** 20(8), 402-403.
9. Jones, R. 2015. Unexplained infectious events leading to deaths and medical admissions. ***BJHCM*** 21(1), 46-47.
10. Jones, R. 2015. Forecasting medical emergency admissions. ***BJHCM*** 21(2), 98-99.
11. Jones, R. 2015. Estimating acute costs. ***BJHCM*** 21(3), 152-153.
12. Jones, R. 2015. Understanding growth in emergency admissions. ***BJHCM*** 21(4), 195-197.
13. Jones, R. 2015. Trends in demand for urgent care. ***J Paramed Pract*** 7(10), 486-488.
14. Beeknoo N, Jones, R. 2016. Using social groups to locate areas of high utilization of critical care. ***BJHCM*** 22(11), 551-560.
15. Beeknoo N, Jones, R. 2017. The demography myth - how demographic forecasting vastly underestimates hospital admissions, and creates the illusion that fewer hospital beds or community-based bed equivalents will be required in the future. ***JAMMR 19(2), 1-27. doi:*** 10.9734/BJMMR/2017/29984
16. Beeknoo, N.; Jones, R. 2017. Information asymmetry in financial forecasting within healthcare and simple methods to overcome this deficiency. ***JAMMR*** 20(4), 1-12. doi: 10.9734/BJMMR/2017/31474
17. Jones, R. 2017. What is driving growth in the English NHS? ***BJHCM*** 23(3), 134-137.
18. Jones, R. 2017. Volatility in emergency admissions per death. ***BJHCM*** 23(11), 552-554.
19. Jones, R. 2019. The nearness to death effect and why NHS pressures are going to intensify. ***J Paramed Pract*** 11(1), 28-30. https://www.magonlinelibrary.com/doi/10.12968/jpar.2019.11.1.28
20. Jones, R. 2019. Ignorance isn't bliss: behind the unequal distribution of end-of-life demand. ***J Paramed Pract*** 11(2), 77-79. https://www.magonlinelibrary.com/doi/abs/10.12968/jpar.2019.11.2.77
21. Jones, R. 2019. End-of-life demand is both highly volatile and shows unexpected trends. ***J Paramed Pract*** 11(3), 122-124. doi: 10.12968/jpar.2019.11.3.122
22. Jones, R. 2019. Unexplained periods of higher deaths contribute to marginal changes in health care demand and health insurance costs: International perspectives. ***International J Health Planning Management 35(3), 673-684.*** https://doi.org/10.1002/hpm.2917

D. Trends in Outpatient Attendance and Follow-up to First Appointment Ratio

1. Beauchant, S.; Jones, R. 1997. Socio-economic and demographic factors in patient non-attendance. ***BJHCM*** 3(10), 523-528.
2. Jones, R. 2000) Outpatient appointments: Feeling a bit peaky. ***Health Service Journal*** 110(5732), 28-31.
3. Jones, R. 2001) Outpatient appointments: A pretty little sum. ***Health Service Journal*** 111(5740), 28-31.
4. Jones, R. 2001) Outpatient waiting times: Quick, quick, slow. ***Health Service Journal*** 111(5778), 20-23.
5. Jones, R. 2009. What next for 18 weeks? ***BJHCM*** 15(8), 404-405.
6. Jones, R. 2009. How to maintain 18 weeks. ***BJHCM*** 15(9), 456-457.
7. Jones, R. 2012. Are there cycles in outpatient costs? ***BJHCM*** 18(5), 276-277.
8. Jones, R. 2012. Increasing GP referrals: collective jump or infectious push? ***BJHCM*** 18(9), 487-495.
9. Jones, R. 2012. GP referral to dermatology: which conditions? ***BJHCM*** 18(11), 594-596.
10. Jones, R. 2012. Trends in outpatient follow-up rates, England 1987/88 to 2010/11. ***BJHCM*** 18(12), 647-655.
11. Jones, R. 2014. Unexpected changes in outpatient first attendance. ***BJHCM*** 20(3), 142-143.
12. Jones, R. 2016. Recent trends in outpatient follow-up rates. ***BJHCM*** 22(2), 92-94.

E. Understanding Sickness Absence Rates—these follow the same curious patterns as deaths (see below)

1. Jones, R. 2016. Unusual trends in NHS staff sickness absence. ***BJHCM*** 22(4), 239-240.
2. Jones, R. 2019. NHS sickness absence – the hidden message that no one is listening to. doi: 10.13140/RG.2.2.13996.31365
3. Jones, R. 2019. Sickness absence trends for the Department for Work & Pensions (England) follow identical hidden on/off patterns to those seen for NHS staff. doi: 10.13140/RG.2.2.27457
4. Jones, R. 2019. All-cause mortality and NHS sickness absence rates in England show a lagged series of step-like changes. ***Achievements of Biology and Medicine*** (Transl) 33(1), 41-43.
5. Jones, R. 2020. NHS sickness absence in England – hidden patterns. ***BJHCM*** 26(4), 1-11. http://doi.org.10.12968/bjhc.2019.0026
6. Jones, R. 2021. Multidisciplinary insights into health care financial risk and hospital surge capacity, Part 2: High population density is associated with enhanced year-to-year volatility in many aspects of poor health including health care worker sickness absence. ***Journal of Health Care Finance. 47(3),*** Multidisciplinary Insights into Health Care Financial Risk and Hospital Surge Capacity, Part 2: High Population Density is Associated with Enhanced Year-to-Year Volatility in Many Aspects of Poor Health Including Health Care Worker Sickness Absence | Jones, PhD | Journal of Health Care Finance (healthfinancejournal.com)

F. Understanding Hospital Mortality

1. Jones, R. 2015. A 'fatal' flaw in hospital mortality models: How spatiotemporal variation in all-cause mortality invalidates hidden assumptions in the models. ***FGNAMB*** 1(3), 82-96. doi: 10.15761/FGNAMB.1000116
2. Jones, R. 2015. Links between bed occupancy, deaths and costs. ***BJHCM*** 21(11), 544-545.
3. Jones, R. 2016. Hospital bed occupancy and deaths (all-cause mortality) in 2015. ***BJHCM*** 22(5), 283-285.
4. Jones, R. 2016. Clear the decks of Summary Hospital-level Mortality Indicator. ***BJHCM*** 22(6), 335-338.
5. Jones, R. 2016. Bed occupancy and hospital mortality. ***BJHCM*** 22(7), 380-381
6. Jones, R. 2016. Hospital deaths and length of stay. ***BJHCM*** 22(8), 424-425.
7. Jones, R. 2016. Hospital mortality rates and changes in activity. ***BJHCM*** 22(10), 519-521.
8. Jones, R.; Sleet G, Pearce O, Wetherill M 2016. Complex changes in blood biochemistry revealed by a composite score derived from Principal Component Analysis: Effects of age, patient acuity, end of life, day-of week, and potential insights into the issues surrounding the 'Weekend' effect in hospital mortality. ***JAMMR*** 18(5), 1-28. doi: 10.9734/BJMMR/2016/29355
9. Jones, R. 2016. Trends in proportion of deaths occurring in hospital. ***BJHCM*** 22 (11), 572-573.
10. Jones, R. 2016. Trends in crude death rates in English hospitals. ***BJHCM*** 22 (12), 616-617.
11. Jones, R. 2017. Is the 'weekend' mortality effect real? ***BJHCM*** 23 (1), 39-41.
12. Jones, R. 2017. In-hospital deaths, all-cause mortality and medical admissions. ***BJHCM*** 23(5), 239-240.
13. Jones, R. 2018. Hospital mortality scores are unduly influenced by changes in the number of admissions. ***European Journal of Internal Medicine*** 51: e35-e37. https://doi.org/10.1016/j.ejim.2018.02.010
14. Jones, R. 2018. Unexpected trends in hospital standardized mortality indicate a novel cause. ***Eur J Internal Med.*** 52: e9-e11. https://doi.org/10.1016/j.ejim.2018.02.018
15. Jones, R. 2018. Hospital mortality scores are unduly influenced by changes in service configuration. ***BJHCM*** 24 (6), 297-301. https://doi.org/10.12968/bjhc.2018.24.6.297

G. COVID-19 and Excess All-cause Mortality

1. Jones, R. 2020. How many extra deaths really occurred in the UK? See http://www.hcaf.biz/2020/Covid_Excess_Deaths.pdf
2. Jones, R. 2021. The COVID-19 counting fiasco: Is the real total of deaths closer to 10 million? In-depth analysis from India and other countries. ***Journal of Health Care Finance 47(3),*** Spring: Special Edition, Special Guest Authors (healthfinancejournal.com)
3. Jones, R. 2021. Did the government of India mislead the world regarding the extent of the COVID-19 problem in its constituent states? ***Journal of Health Care Finance 47(3),*** Spring Special Edition, Special Guest Authors (healthfinancejournal.com)
4. Jones, R. 2021. The true COVID-19 death toll in India is likely to be greater than 1.2 million persons. ***Journal of Health Care Finance 47(3),*** Spring Special Edition, Special Guest Authors (healthfinancejournal.com)
5. Jones, R. 2021. Low COVID-19 testing in the majority of nations has resulted in gross undercounting of infections and deaths. ***Journal of Health Care Finance 47(4),*** Fall Special Edition, Special Guest Authors (healthfinancejournal.com)
6. Jones, R.; Ponomarenko, A. 2023. COVID-related age profiles for SARS-CoV-2 variants in England and Wales and states of the USA (2020 to 2022): impact on all-cause mortality. Infectious Disease Reports 15(5):600-634. https://www.mdpi.com/2036-7449/15/5/58
7. Jones, R.; Ponomarenko, A. 2023. Effect of age, sex, and COVID-19 vaccination history on all-cause mortality: unexpected outcomes in a complex biological and social system. Effect of Age, Sex, and COVID-19 Vaccination History on All-Cause Mortality: Unexpected Outcomes in a Complex Biological and Social System [v1] | Preprints.org
8. Jones, R.; Ponomarenko, A. Pathogens have different age/sex profiles for hospital admission and why COVID-19 variants may behave as if they were 'different' pathogens. Pathogens Have Different Age/Sex Profiles for Hospital Admission and Why COVID-19 Variants Behave as If They Were 'Different' Pathogens[v1] | Preprints.org

H. Understanding Excess Winter Mortality (EWM) and Winter Capacity Planning

1. Jones, R. 2017. The link between seasonal death rates and workloads. ***BJHCM*** 23(9), 448-450.
2. Jones, R. 2017. Anticipated ambulance workload during the 2016/17 winter**.** ***J Paramed Pract*** 9(2), 52-54.
3. Jones, R. 2019. Does on/off switching of deaths modify NHS winter workload? ***J Paramed Pract*** 11(4), 172-173.
4. Jones, R. 2019. Will the winter of 2019/2020 have unusually high service demand? Part 1: Lessons. ***J Paramed Pract*** 11(11), 492-494.
5. Jones, R. 2019. Will the winter of 2019/2020 have unusually high service demand? Part 2: Strategy. ***J Paramed Pract*** 11(12), 538-540.
6. Jones, R. 2020. Excess winter mortality (EWM) and stalling international improvements in life expectancy and mortality rates. ***BJHCM*** 26(12.; https://doi.org/10.12968/bjhc.2020.0020
7. Jones, R. 2021. Excess winter mortality (EWM) as a dynamic forensic tool: Where, when, which conditions, gender, ethnicity, and age. ***Int J Environ Res Public Health*** 18(4); 2161. https://doi.org/10.3390/ijerph18042161
8. Jones, R.; Ponomarenko, A. 2022. Trends in excess winter mortality (EWM) from 1900/01 to 2019/20 – evidence for a complex system of multiple long-term trends. ***Int J Environ Res Public Health*** 19, 3407. Trends in Excess Winter Mortality (EWM) from 1900/01 to 2019/20—Evidence for a Complex System of Multiple Long-Term Trends

9. Jones, R.; Ponomarenko, A. 2022. System complexity in influenza infection and vaccination: effects upon excess winter mortality. ***Infectious Disease Reports*** 14(3), 287-309. https://doi.org/10.3390/idr14030035
10. Jones, R. 2023. Could some vaccines have unanticipated effects against NHS winter pressures? J Paramed Pract 15(6), 6-9.
11. Jones, R. 2023. Winter pressures: policy versus reality. J Paramed Pract 15(7), 170-172.
12. Jones, R.; Ponomarenko, A 2023. COVID-19-Related Age Profiles for SARS-CoV-2 Variants in England and Wales and States of the USA (2020 to 2022): Impact on All-Cause Mortality. Infectious Disease Reports 15(5):600-634. https://www.mdpi.com/2036-7449/15/5/58

I. Excess All-cause Mortality in Influenza and COVID-19 Vaccination

1. Jones, R.; Ponomarenko, A. 2022. Trends in excess winter mortality (EWM) from 1900/01 to 2019/20 – evidence for a complex system of multiple long-term trends. ***Int J Environ Res Public Health*** 19, 3407. https://doi.org/10.3390/ijerph19063407
2. Jones, R.; Ponomarenko, A. 2022. System complexity in influenza infection and vaccination: effects upon excess winter mortality. ***Infectious Disease Reports*** 14(3), 287-309. https://doi.org/10.3390/idr14030035
3. Jones, R.; Ponomarenko, A 2022. Roles for pathogen interference in influenza vaccination, with implications to vaccine effectiveness (VE) and attribution of influenza deaths. ***Infectious Disease Reports*** 14(5), 710- 758. https://doi.org/10.3390/idr14050076
4. Jones, R.; Ponomarenko, A. Roles for age, gender, vaccination history and COVID-19 variants in all-cause mortality: unexpected outcomes in a complex system. ***In review*** Effect of Age, Sex, and COVID-19 Vaccination History on All-Cause Mortality: Unexpected Outcomes in a Complex Biological and Social System [v1] | Preprints.org

J. Was Austerity Directly Linked to Higher Deaths?

1. Jones, R. 2017. Did austerity cause the rise in deaths seen in England and Wales in 2015? ***BJHCM*** 23(9), 418-424.
2. Jones, R. 2017. Essays on rising mortality in England and Wales – a MEDLINE search is not infallible**. *J Roy Soc Med (JRSM)* 110(6),224.** doi: 10.1177/0141076817703864
3. Jones, R. 2017. Role of social group and gender in outbreaks of a novel agent leading to increased deaths, with insights into higher international deaths in 2015. ***Fractal Geometry and Nonlinear Analysis in Medicine and Biology*** 3(1), 1-7. doi: 10.15761/FGNAMB.1000146
4. Jones, R. 2017. Different patterns of male and female deaths in 2015 in English and Welsh local authorities question the role of austerity as the primary force behind higher deaths. ***Fractal Geometry and Nonlinear Analysis in Medicine and Biology*** 3(1), 1-4. doi: 10.15761/FGNAMB.1000145
5. Jones, R. 2019. Unanswered questions for the austerity theory. Healthcare Analysis & Forecasting. doi: 10.13140/RG.2.2.20357.60643 and also https://papers.ssrn.com/sol3/papers.cfm?abstract_id=3319211
6. Jones, R. 2019. Deaths in England and Wales are falling – does the austerity theory still apply? Healthcare Analysis & Forecasting. Available via SSRN. See https://papers.ssrn.com/sol3/papers.cfm?abstract_id=3381403
7. Jones, R. 2019. Austerity in the UK and poor health: were deaths directly affected? ***BJHCM*** 25(11), 337-347.
8. Jones, R. 2020. Excess winter mortality (EWM) and stalling international improvements in life expectancy and mortality rates. ***BJHCM*** 26(12).; https://doi.org/10.12968/bjhc.2020.0020

K. Understanding Hospital Length of Stay (LOS)—central to the next section on bed planning

1. Jones, R. 2009. Length of stay efficiency. ***BJHCM*** 15(11), 563-564.
2. Jones, R. 2010. Benchmarking length of stay. ***BJHCM*** 16(5), 248-250.
3. Jones, R. 2013. Average length of stay in hospitals in the USA. ***BJHCM*** 19(4), 186-191.
4. Jones, R. 2015. Is length of stay a reliable efficiency measure? ***BJHCM*** 21(7), 344-345.
5. Jones, R. 2015. Declining length of stay and future bed numbers. ***BJHCM*** 21(9), 440-441.
6. Jones, R. 2016. Hospital deaths and length of stay. ***BJHCM*** 22(8), 424-425.
7. Jones, R. 2016. Where next for overnight stay admissions, length of stay and bed days? ***BJHCM*** 22(9), 475-477.
8. Jones, R. 2017. Growth in NHS admissions and length of stay: A policy-based evidence fiasco. ***BJHCM*** 23(12), 603-606.
9. Jones, R. 2018. Maternity length of stay efficiency and neonatal admissions. ***BJHCM*** 24(3), 122-124. https://doi.org/10.12968/bjhc.2018.24.3.122

L. Understanding Hospital Bed Planning and Occupancy—bed demand is intrinsically linked to the forecasting demand section.

1. Jones, R. 1997. Emergency admissions: Admissions of difficulty ***Health Service Journal*** 107(5546), 28-31.
2. Jones, R. 2001. Bed occupancy: Don't take it lying down. Health Service Journal 111(5752), 28-31.
3. Jones, R. 2001. New approaches to bed utilisation – making queuing theory practical. Presented at New Techniques for Health and Social Care. Harrogate Management Centre Conference 27th September 2001.
4. Jones, R.. 2002. Principles for effective bed planning and management. Healthcare Analysis & Forecasting. (PDF) Principles for effective bed planning & management (researchgate.net)
5. Jones, R. 2003. Bed management - Tools to aid the correct allocation of hospital beds. Presented at ***Re-thinking bed management – Opportunities and challenges***. Harrogate Management Centre Conference, 27th January 2003. Presented at Re-thinking Bed

Management Opportunities & Challenges. Harrogate Management Centre Conference, London, Thursday 27 th February, 2003 (1library.net)
6. Jones, R. 2009. Emergency admissions and hospital beds. ***BJHCM*** 15(6), 289-296.
7. Jones, R. 2009. Building smaller hospitals. ***BJHCM*** 15(10), 511-512.
8. Jones, R. 2009. Crafting efficient bed pools. ***BJHCM*** 15(12), 614-616.
9. Jones, R. 2010. Myths of ideal hospital size. ***Medical Journal of Australia*** 193(5), 298-300.
10. Jones, R. 2011. Does hospital bed demand depend more on death than demography? ***BJHCM*** 17(5), 190-197. https://doi.org/10.12968/bjhc.2011.17.5.190 1/Method_for_determining_hospital_beds.pdf
11. Jones, R. 2011. Bed days per death: a new performance measure. ***BJHCM*** 17(5), 213.
12. Jones, R. 2011. Hospital bed occupancy demystified. ***BJHCM*** 17(6), 242-248. https://doi.org/10.12968/bjhc.2011.17.6.242
13. Jones, R. 2011. A&E performance and inpatient bed occupancy. ***BJHCM*** 17(6), 256-257.
14. Jones, R. 2011. Bed occupancy – the impact on hospital planning. ***BJHCM*** 17(7), 307-313.
15. Jones, R. 2011. The need for single room accommodation in hospital. ***BJHCM*** 17(7), 316-317.
16. Jones, R. 2011. Demand for hospital beds in English Primary Care Organisations. ***BJHCM*** 17(8), 360-367.
17. Jones, R. 2011. A paradigm shift for bed occupancy. ***BJHCM*** 17(8), 376-377.
18. Jones, R. 2011. Volatility in bed occupancy for emergency admissions. ***BJHCM*** 17(9), 424-430.
19. Jones, R. 2012. Maternity bed occupancy: all part of the equation. **Midwives Magazine** 15(1), http://www.rcm.org.uk/midwives/features/all-part-of-the-equation/
20. Jones, R. 2012. A simple guide to a complex problem – maternity bed occupancy. ***British Journal of Midwifery*** 20(5), 351-357.
21. Jones, R. 2013. A guide to maternity costs – why smaller units have higher costs. ***British Journal of Midwifery*** 21(1), 54-59.
22. Jones, R. 2013. Optimum bed occupancy in psychiatric hospitals. ***Psychiatry On-Line*** http://www.priory.com/psychiatry/psychiatric_beds.htm
23. Jones, R. 2013. The NHS England review of urgent and emergency care. ***BJHCM*** 19(8), 406-407.
24. Jones, R. 2014. Medical bed occupancy and cancelled operations. ***BJHCM*** 20(12), 594-595.
25. Jones, R. 2015. A&E tipping points. ***BJHCM*** 21(5), 248-249.
26. Jones, R. 2015. Bed occupancy, efficiency and infectious outbreaks. ***BJHCM*** 21(8), 396-397.
27. Jones, R. 2015. Links between bed occupancy, deaths and costs. ***BJHCM*** 21(11), 544-545.
28. Jones, R. 2016. Hospital bed occupancy and deaths (all-cause mortality) in 2015. ***BJHCM*** 22(5), 283-285.
29. Beeknoo N, Jones, R. 2016. Achieving economy of scale in critical care, and planning information necessary to support the choice of bed numbers. ***JAMMR*** 17(9),1-15. Achieving Economy of Scale in Critical Care, Planning Information Necessary to Support the Choice of Bed Numbers | *JAMMR* (journaljammr.com)
30. Beeknoo N, Jones, R. 2016. A simple method to forecast next years bed requirements: a pragmatic alternative to queuing theory. ***JAMMR*** 18(4), 1-20. A Simple Method to Forecast Future Bed Requirements: A Pragmatic Alternative to Queuing Theory | *JAMMR* (journaljammr.com)
31. Beeknoo, N.; Jones, R. 2016. The demography myth - how demographic forecasting underestimates hospital admissions and creates the illusion that fewer hospital beds or community-based bed equivalents will be required in the future. ***JAMMR 19(2), 1-27.*** The Demography Myth: How Demographic Forecasting Underestimates Hospital Admissions, and Creates the Illusion that Fewer Hospital Beds and Community-based bed Equivalents, will be Required in the Future | *JAMMR* (journaljammr.com)
32. Jones, R. 2016. Bed occupancy and hospital mortality. ***BJHCM*** 22(7), 380-381.
33. Jones, R. 2017. Is there scope to close acute beds in the STPs. ***BJHCM*** 23(2), 83-85.
34. Jones, R. 2017. What is driving growth in the English NHS? ***BJHCM*** 23(3), 134-137.
35. Jones, R. 2017. Flexibility, hospital bed numbers, and sustainability and transformation plans. ***BJHCM*** 23(7),344-345.
36. Jones, R. 2017. Deaths and acute hospital beds in the Sustainability and Transformation Plans. ***BJHCM*** 23(10), 498-499.
37. Jones, R. 2017. Bed occupancy continues to show on/off switching. ***BJHCM*** 23(11), 515-516.
38. Jones, R. 2018. Local 7-day patterns of on/off switching in acute bed occupancy. ***BJHCM*** 24(2), 100-102.
39. Jones, R. 2018. Do outbreaks of 'Disease X' regulate NHS beds and costs? ***BJHCM*** 24(4), 204-205.
40. Jones, R. 2018. Trends in available beds per death in England. ***BJHCM*** 24(7), 358-359.
41. Jones, R. 2018. Trends in critical care bed numbers in England. ***BJHCM*** 24(10), 516-517.
42. Jones, R. 2018. Hospital beds per death how does the UK compare globally. ***BJHCM*** 24(12), 617-622.
43. Jones, R. 2019. Condition specific growth in occupied beds in England following a sudden and unexpected increase in deaths. ***BJHCM*** 25(6), 1-8.
44. Jones, R. 2019. Have doctors and the public been misled regarding hospital bed requirements? ***BJHCM*** 25 (7), 242-250.
45. Jones, R. 2019. A pragmatic method to compare hospital bed provision between countries and regions: Beds in the states of Australia. ***Intl J Health Plan Mgmt*** 35(3), 746-759. https://doi.org/10.1002/hpm.2950
46. Jones, R. 2020. Curious patterns in hospital bed occupancy data. ***BJHCM*** 25(3), 71-72. https://doi.org/10.12968/bjhc.2020.0014
47. Jones, R. 2020. Would the United States have had too few beds for universal emergency care in the event of a more widespread Covid-19 epidemic? ***Int J Environmental Research and Public Health*** *17(14), 5210.* https://doi.org/doi:10.3390/ijerph17145210

48. Jones, R. 2020. How many medical beds does a country need? An international perspective. ***BJHCM*** 26(9), 248-259. https://doi.org/10.12968/bjhc.2020.0028
49. Jones, R. 2021. Were the hospital bed reductions proposed by English Clinical Commissioning Groups (CCGs) in the Sustainability and Transformation Plans (STPs) achievable? Insights from a new model to compare international bed numbers. ***Intl J Health Planning and Management*** 36(2), 459-481**.** https://doi.org/10.1002/hpm.3094
50. Jones, R. 2021. Multidisciplinary insights into health care financial risk and hospital surge capacity, Part 1: Nearness to death, infectious outbreaks, and Covid-19. ***Journal of Health Care Finance. 47(3),*** Vol. 47, No. 3, Winter 2021 (healthfinancejournal.com)
51. Jones, R. 2021. With 1.9 million hospital beds why is India struggling? ***Journal of Health Care Finance. 47(3),*** Special Guest Authors (healthfinancejournal.com)
52. Jones, R. 2021. Does the ageing population correctly predict the need for medical beds over the next 40 years? Part one: Fundamental principles ***BJHCM*** 27(8), ***doi: 10.12968/bjhc.2020.0156*** Does the ageing population correctly predict the need for medical beds?: Part one: fundamental principles | British Journal of Healthcare Management (magonlinelibrary.com)
53. Jones, R. 2021. Does the ageing population correctly predict the need for medical beds over the next 40 years? Part two: Wider implications. ***BJHCM*** 27(10), ***doi: 10.12968/bjhc.2021.0116*** Does the ageing population correctly predict the need for medical beds? Part two: wider implications | British Journal of Healthcare Management (magonlinelibrary.com)
54. Jones, R. 2021. A simple method to validate medical bed number calculations. ***Eur J Internal Medicine 94: P108-P109.*** https://doi.org/10.1016/j.ejim.2021.08.009
55. Jones, R. 2022. A pragmatic method to compare international critical care beds: implications to pandemic preparedness and non-pandemic planning. ***Int J Health Planning and Management 37(4), 2167-2182.*** https://doi.org/10.1002/hpm.3458
56. Jones, R. 2022. A model to compare international hospital bed numbers, including a case study on the role of indigenous people on acute 'occupied' bed demand in Australian states. Int J Environ Res Public Health 19: 11239. https://doi.org/10.3390/ijerph191811239
57. Jones, R. 2023. Addressing the knowledge deficit in hospital bed planning, defining an optimum region for the number of different types of hospital beds in an effective health care system. *Int. J. Environ. Res. Public Health* **2023**, *20*(24), 7171; **https://doi.org/10.3390/ijerph20247171**
58. Jones, R.P. Capacity Planning (Capital, Staff and Costs) of Inpatient Maternity Services: Pitfalls for the Unwary. Int. J. Environ. Res. Public Health 2025, 22, 87. https://doi.org/10.3390/ijerph22010087

M. The Link Between Deaths (All-cause Mortality) and Medical Emergency Admissions

1. Jones, R. 2012. Diagnoses, deaths and infectious outbreaks. ***BJHCM*** 18(10), 539-548.
2. Jones, R. 2013. An unexplained increase in deaths during 2012. ***BJHCM*** 19(5), 248-253.
3. Jones, R. 2013. Analysing excess winter mortality: 2012/13. ***BJHCM*** 19(12), 601-605.
4. Jones, R. 2014. Increased deaths in 2012: which conditions? ***BJHCM*** 20(1), 45-47.
5. Jones, R. 2014. Trends in death and end-of-life costs in the UK. ***BJHCM*** 20(6), 298-299.
6. Jones, R. 2014. Trends in emergency admissions per death. ***BJHCM*** 20(9), 446-447.
7. Jones, R. 2015. A previously uncharacterized infectious-like event leading to spatial spread of deaths across England and Wales: Characteristics of the most recent event and a time series for past events. ***JAMMR*** 5(11), 1361-1380. doi: 10.9734/BJMMR/2015/14285
8. Jones, R. 2015. Unexplained infectious events leading to deaths and medical admissions in Belfast**.** ***BJHCM*** 21(1), 46-47.
9. Jones, R. 2015. Unexpected Increase in Deaths from Alzheimer's, Dementia and Other Neurological Disorders in England and Wales during 2012 and 2013. ***Journal of Neuroinfectious Diseases*** 6:172. doi: 10.4172/2314-7326.1000172
10. Jones, R. 2015. Influenza-like-illness, deaths and health care costs. ***BJHCM*** 21(12), 587-589.
11. Jones, R. 2016. The real reason for the huge NHS overspend? ***BJHCM*** 22(1), 40-42.
12. Jones, R. 2016. Rising emergency admissions in the UK and the elephant in the room. ***Epidemiology: Open Access*** 6(4), 1000261
13. doi: 10.4172/2161-1165.1000261
14. Jones, R. 2016. Deaths and the marginal changes in healthcare costs ***BJHCM*** 22(10), 503-509.
15. Jones, R. 2016. Trend in proportion of deaths occurring in hospital. ***BJHCM*** 22(11), 572-573.
16. Jones, R. 2017. In-hospital deaths, all-cause mortality and medical admissions. ***BJHCM*** 23(5), 239-240.
17. Jones, R. 2017. Anticipated NHS demand in 2017/18. ***J Paramed Pract*** 9(6), 236-237.
18. Jones, R. 2017. What government data on death rates fail to show. ***BJHCM*** 23(8), 572-573.
19. Jones, R. 2017. A reduction in acute thrombotic admissions during a period of unexplained increased deaths and medical admissions in the UK. ***European Journal of Internal Medicine*** 46: e31-e33. http://dx.doi.org/10.1016/j.ejim.2017.09.007
20. Jones, R. 2018. Volatility in emergency admissions per death. ***BJHCM*** 23(11), 554-556.
21. Jones, R. 2018. Admissions for certain conditions show explosive growth in England following a sudden and unexpected increase in deaths. ***European Journal of Internal Medicine***. 2018; 54: e33-e35. doi: https://doi.org/10.1016/j.ejim.2018.03.005
22. Jones, R. 2018. Do outbreaks of 'Disease X' regulate NHS beds and costs? ***BJHCM*** 24(4), 204-205.
23. Jones, R. 2018. Clinical workload trends. ***BJHCM*** 24(6), 308-309.
24. Jones, R. 2018. Deaths in the UK show a large increase in 2018. ***BJHCM*** 24(8), 410-411.
25. Jones, R. 2018. Will 2018 set a record for deaths? ***BJHCM*** 24(9), 464-465.
26. Jones, R. 2018. End-of-life, unusual syndromic symptoms, and periods of high physician workload. ***Achievements of Biology and Medicine*** 31(1), 46-51. *http://files.odmu.edu.ua/biomed/2018/01/d181_46.pdf*

27. Jones, R. 2019. A need for transparency and evidence-based discussion. ***J Paramed Pract*** 11(5), 219-220.
28. Jones, R. 2019. The calendar year fallacy: The danger of reliance on calendar year data in end-of-life capacity and financial planning. ***The International Journal of Health Planning and Management*** 3***34(4), e1533-e1543.*** https://doi.org/10.1002/hpm.2838
29. Jones, R. 2019. Austerity in the UK and poor health: were deaths affected? ***BJHCM*** 25(11), 337-347.

N. Financial Risk in Healthcare—see sections on deaths/admissions and small-area spatiotemporal patterns to understand the basis for the very high financial risk in healthcare. Financial and capacity risk are interlinked.

1. Jones, R. Financial and operational risk in healthcare. ***HMC Conference 'Re-shaping Acute Services for the 21st Century', Barbican Centre, London. 26*** September 2002. (99+) (PDF) Financial and operational risk in health care provision and commissioning | Rodney Jones - Academia.edu
2. Jones, R. 2004. Financial risk in healthcare provision and contracts. ***Proceedings of the 2004 Crystal Ball User Conference***, June 16-18th, 2004. Denver, Colarado, USA. (11) (PDF) Financial risk in healthcare provision and contracts (researchgate.net)
3. Jones, R. 2008. Financial risk in practice-based commissioning. ***BJHCM*** 14(5), 199-204.
4. Jones, R. 2008. Financial risk in health purchasing Risk pools. ***BJHCM*** 14(6), 240-245.
5. Jones, R. 2008. Financial risk at the PCT/PBC Interface. ***BJHCM*** 14(7), 288-293.
6. Jones, R. 2009. Emergency admissions and financial risk. ***BJHCM*** 15(7), 344-350.
7. Jones, R. 2009. The actuarial basis for financial risk in practice-based commissioning and implications to managing budgets. ***Primary Health Care Research & Development*** 10(3), 245-253. https://doi.org/10.1017/S1463423609990089
8. Jones, R. 2010. What is the financial risk in GP Commissioning? ***British Journal of General Practice*** 60(578), 700-701. https://www.ncbi.nlm.nih.gov/pmc/articles/PMC2930237/
9. Jones, R. 2010. Cyclic factors behind NHS deficits and surpluses. ***BJHCM*** 16(1), 48-50.
10. Jones, R. 2010. Do NHS cost pressures follow long-term patterns? ***BJHCM*** 16(4), 192-194.
11. Jones, R. 2010. Nature of health care costs and financial risk in commissioning. ***BJHCM*** 16(9), 424-430.
12. Jones, R. 2010. Trends in programme budget expenditure. ***BJHCM*** 16(11), 518-526.
13. Jones, R. 2011. Cycles in inpatient waiting time. ***BJHCM*** 17(2), 80-81.
14. Jones, R. 2012. Time to re-evaluate financial risk in GP commissioning. ***BJHCM*** 18(1), 39-48.
15. Jones, R. 2012. Gender ratio and cycles in population health costs. ***BJHCM*** 18(3), 164-165.
16. Jones, R. 2012. Why is the 'real world' financial risk in commissioning so high? ***BJHCM*** 18(4), 216-217.
17. Jones, R. 2012. Volatile inpatient costs and implications to CCG financial stability. ***BJHCM*** 18(5), 251-258.
18. Jones, R. 2012. Cancer care and volatility in commissioning. ***BJHCM*** 18(6), 315-324.
19. Jones, R. 2012. Gender and financial risk in commissioning. ***BJHCM*** 18(6), 336-337.
20. Jones, R. 2012. End of life care and volatility in costs. ***BJHCM*** 18(7), 374-381.
21. Jones, R. 2012. Age and financial risk in healthcare costs. ***BJHCM*** 18(7), 388-389.
22. Jones, R. 2012. High risk categories and risk pooling in healthcare costs. ***BJHCM*** 18(8), 430-435.
23. Jones, R. 2012. Year-to-year volatility in medical admissions. ***BJHCM*** 18(8), 448-449.
24. Jones, R. 2012. Risk in GP commissioning: the loss ratio. ***BJHCM*** 18(11), 605-606.
25. Jones, R. 2012. Financial risk in GP commissioning: lessons from Medicare. ***BJHCM*** 18(12), 656-657.
26. Jones, R. 2013. Financial risk and volatile elderly diagnoses. ***BJHCM*** 19(2), 94-96.
27. Jones, R. 2013. Financial risk and volatile childhood diagnoses. ***BJHCM*** 19(3), 148-149.
28. Jones, R. 2013. Environmental volatility and healthcare costs. ***BJHCM*** 19(4), 198-199.
29. Jones, R. 2013. What every GP needs to know about financial risk in commissioning. ***General Practice Online*** http://www.priory.com/family_medicine/GP_commissioning_risk.htm
30. Jones, R. 2013. The funding dilemma: a lagged cycle in cancer costs. ***BJHCM*** 19(12), 601-605.
31. Jones, R. 2014. Financial volatility in NHS contracts. ***BJHCM*** 20(10), 489-491.
32. Jones, R. 2016. The real reason for the huge NHS overspend? ***BJHCM*** 22(1), 40-42.
33. Jones, R. 2019. Financial risk in health and social care budgets. ***BJHCM*** 25(2), 79-84.
34. Jones, R. 2019. The calendar year fallacy: The danger of reliance on calendar year data in actuarial calculations. ***Intl J Health Plan Management 34(4), e1533-e1543.*** https://doi.org/10.1002/hpm.2838
35. Jones, R. 2021. Multidisciplinary insights into health care financial risk and hospital surge capacity, Part 1: Nearness to death, infectious outbreaks, and Covid-19. ***Journal of Health Care Finance. 47(3),*** Vol. 47, No. 3, Winter 2021 (healthfinancejournal.com)
36. Jones, R. 2021. Multidisciplinary insights into health care financial risk and hospital surge capacity, Part 2: High population density is associated with enhanced year-to-year volatility in many aspects of poor health including health care worker sickness absence. ***Journal of Health Care Finance. 47(3),*** Vol. 47, No. 3, Winter 2021 (healthfinancejournal.com)
37. Jones, R. 2021. Multidisciplinary insights into health care financial risk and hospital surge capacity, Part 3: Outbreaks of a new type or kind of disease create unique risk patterns and confounds traditional trend analysis. ***Journal of Health Care Finance. 47(3),*** Vol. 47, No. 3, Winter 2021 (healthfinancejournal.com)

38. Jones, R. 2021. Multidisciplinary insights into health care financial risk and hospital surge capacity, Part 4: What size does a health insurer or health authority need to be to minimise risk? ***Journal of Health Care Finance. 47(3),*** Vol. 47, No. 3, Winter 2021 (healthfinancejournal.com)
39. Jones, R. 2021. The epidemiology of health and social care cost and capacity shocks. ***Odessa Medical Journal*** 2021(5), doi: 10.54229/2226-2008-2021-5-9

O. Limitations of the Healthcare Resource Group (HRG) Tariff—*this is intrinsically linked to financial risk (see above)*

1. Jones, R. 2008. Limitations of the HRG tariff: excess bed days. ***BJHCM*** 14(8), 354-355.
2. Jones, R. 2008. Limitations of the HRG tariff: day cases. ***BJHCM*** 14(9), 402-404.
3. Jones, R. 2008. A case of the emperor's new clothes? ***BJHCM*** 14(10), 460-461.
4. Jones, R. 2008. Limitations of the HRG tariff: the trim point. ***BJHCM*** 14(11), 510-513.
5. Jones, R. 2008. Costing orthopaedic interventions. ***BJHCM*** 14(12), 539-547.
6. Jones, R. 2009. Limitations of the HRG tariff: efficiency comparison. ***BJHCM*** 15(1), 40-43.
7. Jones, R. 2009. Limitations of the HRG tariff: the RCI. ***BJHCM*** 15(2), 92-95.
8. Jones, R. 2009. Limitations of the HRG tariff: local adjustments. ***BJHCM*** 15(3), 144-147.
9. Jones, R. 2010. A maximum price tariff. ***BJHCM*** 16 (3), 146-147.
10. Jones, R. 2010. Nature of health care costs and the HRG tariff. ***BJHCM*** 16(9), 451-452.
11. Jones, R. 2010. Emergency assessment tariff: lessons learned. ***BJHCM 16***(12), 574-583.
12. Jones, R. 2010. High efficiency or unfair financial gain? ***BJHCM*** 16(12), 585-586.
13. Jones, R. 2010. Is the HRG tariff fit for purpose? nhsManagers.net: Managers Briefing
14. Jones, R. 2011. Impact of the A&E targets in England. ***BJHCM*** 17(1), 16-22.
15. Jones, R. 2011. Costs of paediatric assessment. ***BJHCM*** 17(2), 57-63.
16. Jones, R. 2011. Is the short stay emergency tariff a valid currency? ***BJHCM*** 17(10), 496-497.
17. Jones, R. 2011. Limitations of the HRG tariff: the national average. ***BJHCM*** 17(11), 556-557.
18. Jones, R. 2011. Limitations of the HRG tariff: gross errors. ***BJHCM*** 17(12), 608-609.
19. Jones, R. 2012. Is the Health Resource Group (HRG) tariff fit for purpose? ***BJHCM*** 18(1), 52-53.
20. Jones, R. 2012. Limitations of the HRG tariff. Healthcare Analysis & Forecasting.
21. Jones, R. 2013. A guide to maternity costs - why smaller units cost more. ***British Journal of Midwifery*** 21(1), 54-59. https://www.magonlinelibrary.com/doi/abs/10.12968/bjom.2013.21.1.54

P.* Funding and the Funding Formula —*this is intrinsically linked to financial risk (see above)

1. Jones, R. 1994. GP Fundholding: Readies reckoner. ***Health Service Journal*** 104 (10th Feb), 31.
2. Jones, R. 2011. Infectious outbreaks and the capitation formula. ***BJHCM*** 17(1), 36-38.
3. Jones, R. 2013. A fundamental flaw in person-based funding. ***BJHCM*** 19(1), 32-38.
4. Jones, R. 2013. Population density and healthcare costs. ***BJHCM*** 19(1), 44-45.
5. Jones, R.; Kellet, J. 2018. The way healthcare is funded is wrong: it should be linked to deaths as well as age, gender and social deprivation. ***Acute Medicine*** 17(4), 189-193. See https://acutemedjournal.co.uk/original-articles/the-way-healthcare-is-funded-is-wrong-it-should-be-linked-to-deaths-as-well-as-age-gender-and-social-deprivation/
6. Jones, R. 2021. Multidisciplinary insights into health care financial risk and hospital surge capacity, Part 4: What size does a health insurer or health authority need to be to minimise risk? ***Journal of Health Care Finance. 47(3),*** Multidisciplinary insights into health care financial risk and hospital surge capacity, Part 4: What size does a health insurer or health authority need to be to minimise risk? | Jones, PhD | Journal of Health Care Finance (healthfinancejournal.com)
7. Jones, R. Are the English Integrated care Boards subjuct to unjustified cost pressures? BJGP Life. 2 August 2024, https://bjgplife.com/are-the-english-integrated-care-boards-subject-to-unjustified-cost-pressures/
8. Jones, R. Why perpetuate NHS funding gaps? BJGP Life, 8 August 2024. https://bjgplife.com/why-perpetuate-nhs-funding-gaps/

Q. Outbreaks of a New Type of Infectious Immune Impairment Affecting Deaths and Medical Admissions—The World Health Organisation's Disease X?

1. Jones, R. 2010. Unexpected, periodic and permanent increase in medical inpatient care: man-made or new disease. ***Medical Hypotheses*** 74: 978-83. http://dx. doi.org/10.1016/j.mehy.2010.01.011
2. Jones, R. 2010. Can time-related patterns in diagnosis for hospital admission help identify common root causes for disease expression. ***Medical Hypotheses*** 75: 148-154. http://dx. doi.org/10.1016/j.mehy.2010.02.009
3. Jones, R. 2010. The case for recurring outbreaks of a new type of infectious disease across all parts of the United Kingdom. ***Medical Hypotheses*** 75: 452-457. http://dx. doi.org/10.1016/j.mehy.2010.04.023
4. Jones, R. 2013. Do recuring outbreaks of a type of infectious immune impairment trigger cyclic changes in the gender ratio at birth? ***Biomedicine International*** 4(1), 26-39. https://www.bmijournal.org/index.php/bmi/article/download/27/25
5. Jones, R. 2013. Widespread outbreaks of a subtle condition leading to hospitalization and death. ***Epidemiology: Open access*** 4(3), 137. doi: 10.4172/2161-1165.1000137
6. Jones, R. 2014. Unexpected single-year-of-age changes in the elderly mortality rate in 2012 in England and Wales. ***JAMMR*** 4(16), 3196-3207. https://journaljammr.com/index.php/JAMMR/article/view/15127

7. Jones, R. 2015. Unexpected and Disruptive Changes in Admissions Associated with an Infectious-like Event Experienced at a Hospital in Berkshire, England around May of 2012. ***JAMMR*** 6(1), 56-76. [Unexpected and Disruptive Changes in Admissions Associated with an Infectious-like Event Experienced at a Hospital in Berkshire, England around May of 2012 | *JAMMR* (journaljammr.com)]
8. Jones, R. 2015. A previously uncharacterized infectious-like event leading to spatial spread of deaths across England and Wales: Characteristics of the most recent event and a time series for past events. ***JAMMR*** 5(11), 1361-1380. [A Previously Uncharacterized Infectious-like Event Leading to Spatial Spread of Deaths Across England and Wales: Characteristics of the most Recent Event and a Time Series for Past Events | *JAMMR* (journaljammr.com)]
9. Jones, R. 2015. Are emergency admissions contagious? ***BJHCM*** 21(5), 227-235.
10. Jones, R. 2015. Recurring outbreaks of an infection apparently targeting immune function, and consequent unprecedented growth in medical admission and costs in the United Kingdom: A review. ***Brit J Med and Medical Research*** 6(8), 735-770. [Recurring Outbreaks of an Infection Apparently Targeting Immune Function, and Consequent Unprecedented Growth in Medical Admission and Costs in the United Kingdom: A Review | *JAMMR* (journaljammr.com)]
11. Jones, R. 2016. A presumed infectious event in England and Wales during 2014 and 2015 leading to higher deaths in those with neurological and other disorders. ***J Neuroinfectious Diseases*** 7(1), 1000213 doi: 10.4172/2314-7326.1000213
12. Jones, R. 2017. A reduction in acute thrombotic admissions during a period of unexplained increased deaths and medical admissions in the UK. ***European Journal of Internal Medicine*** 46: e31-e33 doi: http://dx.doi.org/10.1016/j.ejim.2017.09.007
13. Jones, R. 2017. Age-specific and year of birth changes in hospital admissions during a period of unexplained higher deaths in England. ***European Journal of Internal Medicine*** 45: 2-4. doi: http://dx.doi.org/10.1016/j.ejim.2017.09.039
14. Jones, R. 2018. Periods of unexplained higher deaths and medical admissions have occurred previously – but were apparently ignored, misinterpreted, or not investigated. ***Eur J Internal Medicine*** 40: e18-e20. https://doi.org/10.1016/j.ejim.2017.11.004
15. Jones, R. 2018. Do outbreaks of 'Disease X' regulate NHS beds and costs? ***BJHCM*** 24(4), 204-205.
16. Jones, R. 2018. Deaths in 2017 reached a new (unexpected) high. ***BJHCM*** 24(5), 256-257.
17. Jones, R. 2019. Unusual international behaviour of deaths suggests a possible new kind of disease outbreak. Healthcare Analysis & Forecasting. Via SSRN http://ssrn.com/abstract=3364795
18. Jones, R. 2019. Why are deaths in the UK behaving in such a peculiar way? Healthcare Analysis & Forecasting. doi: 10.13140/RG.2.2.13685.42728

R. Small-area Spatiotemporal Patterns in the Spread of a New Type or Kind of Infectious Disease (The WHO Disease X?)

1. Jones, R. 2013. A recurring series of infectious-like events leading to excess deaths, emergency department attendances and medical admissions in Scotland. ***Biomedicine International*** 4(2), 72-86. http://www.bmijournal.org/index.php/bmi/article/view/35
2. Jones, R. 2014. Infectious-like Spread of an Agent Leading to Increased Medical Admissions and Deaths in Wigan (England), during 2011 and 2012. ***JAMMR*** 4(28), 4723-4741 [Infectious-like Spread of an Agent Leading to Increased Medical Admissions and Deaths in Wigan (England), during 2011 and 2012 | *JAMMR* (journaljammr.com)]
3. Jones, R.; Beauchant, S. 2015. Spread of a new type of infectious condition across Berkshire in England between June 2011 and March 2013: Effect on medical emergency admissions. ***JAMMR*** 6(1), 126-148. [Small Area Spread of a New Type of Infectious Condition across Berkshire in England between June 2011 and March 2013: Effect on Medical Emergency Admissions | *JAMMR* (journaljammr.com)]
4. Jones, R. 2015. Simulated rectangular wave infectious-like events replicate the diversity of time-profiles observed in real-world running 12-month totals of admissions or deaths. ***FGNAMB*** 1(3), 78-79. doi: 10.15761/FGNAMB.1000114
5. Jones, R. 2015. A new type of infectious outbreak? ***SMU Medical Journal*** 2(1), 19-25. http://smu.edu.in/content/dam/manipal/smu/documents/Journal%20Issue%203/A%20New%20Type%20of%20Infectious%20Outbreak.pdf
6. Jones, R. 2015. Small area spread and step-like changes in emergency medical admissions in response to an apparently new type of infectious event. ***FGNAMB 1***(2), 42-54. doi: 10.15761/FGNAMB.1000110
7. Jones, R. 2015. Infectious-like spread of an agent leading to increased medical hospital admission in the North East Essex area of the East of England. ***FGNAMB*** 1(3), 98-111. doi: 10.15761/FGNAMB.1000117
8. Jones, R. 2015. A time series of infectious-like events in Australia between 2000 and 2013 leading to extended periods of increased deaths (all-cause mortality) with possible links to increased hospital medical admissions. ***International Journal of Epidemiologic Research*** 2(2), 53-67. http://ijer.skums.ac.ir/article_12869_2023.html
9. Jones, R. 2015. Deaths and international health care expenditure. ***BJHCM*** 21(10), 491-493.
10. Jones, R. 2016. A fatal flaw in mortality-based disease surveillance. ***BJHCM*** 22(3), 143-145.
11. Jones, R. 2016. Deaths in English Lower Super Output Areas (LSOA) show patterns of very large shifts indicative of a novel recurring infectious event. ***SMU Medical Journal*** 3(2), 23-36. https://pdfs.semanticscholar.org/c3aa/71a1b78e053cba4a871093dd43aa896d9ef6.pdf
12. Jones, R. 2016. A regular series of unexpected and large increases in total deaths (all-cause mortality) for male and female residents of mid super output areas (MSOA) in England and Wales: How high-level analysis can miss the contribution from complex small-area spatial spread of a presumed infectious agent. ***Fractal Geometry and Nonlinear Analysis in Medicine and Biology*** 2(2), 1-13. doi: 10.15761/FGNAMB.1000129

13. Jones, R. 2017. Outbreaks of a presumed infectious agent associated with changes in fertility, stillbirth, congenital abnormalities and the gender ratio at birth. ***JAMMR*** 20(8), 1-36. doi: 10.9734/BJMMR/2017/32372
14. Jones, R. 2017. Outbreaks of a presumed infectious pathogen creating on/off switching in deaths. ***SDRP Journal of Infectious Diseases Treatment and Therapy*** 1(1), 1-6. http://www.openaccessjournals.siftdesk.org/articles/pdf/Outbreaks-of-a-presumed-infectious-pathogen-creating-on-off-switching-in-deaths20170606102727.pdf
15. Jones, R. 2017. Year-to-year variation in deaths in English Output Areas (OA), and the interaction between a presumed infectious agent and influenza in 2015. ***SMU Medical Journal*** **4(2),** 37-69. http://smu.edu.in/content/dam/manipal/smu/smims/Volume4No2July2017/SMU%20Med%20J%20(July%202017.%20-%204.pdf
16. Jones, R. 2017. Deaths and medical admissions – what is happening in the UK? ***FGNAMB*** 3(1), 1. doi: 10.15761/FGNAMB.1000143
17. Jones, R. 2018. Deaths and medical admissions in the UK show an unexplained and sustained peak after 2011. ***Eur J Internal Med 47: e14-e16.*** DOI: http://dx.doi.org/10.1016/j.ejim.2017.09.021

S. Cytomegalovirus (CMV) and Human Disease
1. Jones, R. 2011. CMV and health care costs. ***BJHCM*** 17(4), 168-169.
2. Jones, R. 2013. Could cytomegalovirus be causing widespread outbreaks of chronic poor health? In ***Hypotheses in Clinical Medicine,*** pp 37-79, Eds M. Shoja, et al. New York: Nova Science Publishers Inc.
3. Jones, R. 2014. A Study of an unexplained and large increase in respiratory deaths in England and Wales: Is the pattern of diagnoses consistent with the potential involvement of Cytomegalovirus? ***JAMMR*** 4(33), 5179-5192. A Study of an Unexplained and Large Increase in Respiratory Deaths in England and Wales: Is the Pattern of Diagnoses Consistent with the Potential Involvement of Cytomegalovirus? | ***JAMMR*** (journaljammr.com)
4. Jones, R.; Goldeck, D. 2014. Unexpected and unexplained increase in death due to neurological disorders in 2012 in England and Wales: Is cytomegalovirus implicated? ***Medical Hypotheses*** 83(1), 25-31. http://dx. doi.org/10.1016/j.mehy.2014.04.016
5. Jones, R. 2015. Roles for cytomegalovirus in infection, inflammation and autoimmunity. In ***Infection and Autoimmunity***, 2nd Edition, Eds: N Rose, et al. Elsevier: Amsterdam. Chapter 18, pp 319-357. doi:10.1016/B978-0-444-63269-2.00068-4
6. Jones, R. 2015. An unexpected increase in adult appendicitis in England 2000/01 to 2012/13), Could cytomegalovirus (CMV) be a risk factor? ***JAMMR*** 5(5), 579-603. An Unexpected Increase in Adult Appendicitis in England 2000/01 to 2012/13), Could Cytomegalovirus (CMV) be A Risk Factor? | ***JAMMR*** (journaljammr.com)
7. Jones, R. 2016. Is cytomegalovirus involved in recurring periods of higher than expected death and medical admissions, occurring as clustered outbreaks in the northern and southern hemispheres? ***JAMMR*** 11(2), 1-31. https://journaljammr.com/index.php/JAMMR/article/view/9833
8. Jones, R. 2017. International outbreaks of a novel type of infectious immune impairment: A call to action. **Досягнення біології та медицини** (***Achievements of Biology and Medicine - transl)*** 29(1), 75-81. http://files.odmu.edu.ua/biomed/2017/01/d171_75.pdf or BIO171a.pm6 (hcaf.biz)
9. Jones, R. 2017. Outbreaks of a presumed infectious agent associated with changes in fertility, stillbirth, congenital abnormalities and the gender ratio at birth. ***JAMMR*** 20(8), 1-36. Outbreaks of a Presumed Infectious Agent Associated with Changes in Fertility, Stillbirth, Congenital Abnormalities and the Gender Ratio at Birth | ***JAMMR*** (journaljammr.com)

Dr Rodney Jones has over 30-years' experience in healthcare demand forecasting, capacity and bed number planning, and financial risk in healthcare purchasing and commissioning. He has published over 300 papers and reports on these topics. His 'Research Interest' score places him in the top 10%of international researchers out of 25+ million ResearchGate members (top 25% for researchers of the same age). His papers have over 1,000 citations https://www.researchgate.net/profile/Rodney_Jones. Dr Rodney Jones can be contacted at hcaf_rod@yahoo.co.uk or +44 (0)7890 640399

ORCHID ID: 0000-0002-4810-7638
