# Supplementary material for: Capacity Planning for Small Hospitals and Departments Illustrated Using Maternity and Paediatrics Departments: Roles for Weighted Population Density, Seasonality and Size, Myths Around Length of Stay and Factors Influencing Costs and Funding
Source: Int J Environ Res Public Health. 2026 May 27;23(6):711. doi: 10.3390/ijerph23060711 (PMC13299548; doi:10.3390/ijerph23060711)
Supplement: Supplementary file 1 [file ijerph-23-00711-s001.zip › Supplementary Table S1 Seasonality in European countries.pdf]

Table S1. Seasonality calculations for European countries (2006-2015). Data from Eurostat [29]. The first two calculations use a moving 12-month calculation while the final column calculates the average for each month over the 10 years.

Country	Annual births	Highest relative to lowest			Highest relative to average			Fixed month average max
		Maximum	Median	Q3	Maximum	Median	Q3	
Liechtenstein	365	189%	84%	102%	66%	31%	36%	16%
Andorra	835	96%	58%	82%	38%	25%	31%	15%
Malta	4,106	189%	32%	51%	53%	15%	19%	9%
Iceland	4,645	35%	25%	29%	21%	13%	17%	9%
Luxembourg	5,913	31%	23%	24%	21%	10%	13%	11%
Montenegro	7,813	76%	31%	38%	40%	13%	16%	8%
Cyprus	9,517	55%	40%	46%	26%	18%	22%	18%
Estonia	15,072	37%	20%	25%	18%	9%	12%	10%
Slovenia	21,541	31%	20%	21%	14%	9%	9%	7%
Latvia	22,004	33%	19%	24%	18%	10%	12%	9%
Yugoslav Rep. of Macedonia	23,657	34%	23%	27%	17%	11%	13%	9%
Kosovo (UN Resolution)	27,351	81%	41%	43%	99%	15%	19%	14%
Lithuania	31,207	30%	22%	26%	15%	10%	12%	9%
Bosnia and Herzegovina	35,226	68%	26%	36%	39%	13%	16%	15%
Moldova	39,730	46%	33%	37%	27%	14%	17%	14%
Croatia	42,268	30%	23%	25%	18%	11%	13%	10%
Armenia	42,729	50%	45%	47%	26%	24%	25%	24%
Slovakia	57,966	36%	23%	29%	20%	10%	12%	10%
Georgia	58,381	70%	29%	30%	20%	12%	13%	10%
Finland	59,973	21%	16%	20%	11%	6%	7%	5%
Norway	60,899	36%	25%	28%	13%	9%	10%	9%
Denmark	61,943	24%	19%	21%	12%	9%	10%	7%
Serbia	68,958	33%	24%	28%	13%	11%	12%	11%
Ireland	72,202	31%	12%	13%	13%	6%	8%	6%
Bulgaria	73,690	23%	18%	19%	16%	9%	10%	8%
Austria	80,403	19%	15%	18%	11%	8%	8%	8%
Switzerland	81,544	18%	13%	14%	10%	7%	8%	9%
Hungary	95,390	28%	19%	23%	13%	10%	11%	9%
Portugal	96,816	28%	20%	23%	17%	12%	14%	11%
Greece	107,922	37%	27%	28%	16%	13%	14%	11%
Belarus	111,807	31%	23%	25%	15%	10%	12%	8%
Sweden	113,924	32%	29%	31%	11%	8%	9%	7%
Czech Republic	114,081	24%	18%	20%	12%	9%	10%	8%
Belgium	128,399	15%	11%	13%	8%	6%	7%	4%
European Free Trade Assoc.	147,452	20%	17%	19%	10%	7%	8%	7%
Azerbaijan	166,035	69%	43%	61%	22%	16%	18%	14%
Netherlands	182,649	18%	14%	16%	10%	7%	8%	7%
Romania	210,506	37%	22%	25%	20%	12%	14%	11%
Poland	396,798	36%	23%	28%	13%	9%	11%	9%
Spain	474,856	16%	12%	13%	9%	6%	7%	5%
Ukraine	502,250	39%	27%	31%	21%	13%	15%	10%
Italy	551,376	33%	25%	28%	17%	12%	13%	10%
Germany	697,971	24%	20%	21%	13%	10%	11%	10%
United Kingdom	800,944	14%	9%	11%	8%	5%	6%	5%
France	836,679	13%	9%	11%	7%	4%	5%	4%
Turkey	1,304,766	41%	36%	37%	16%	13%	14%	9%
Russia	1,768,354	28%	17%	18%	13%	8%	9%	7%
European Union (28 countries)	5,366,117	16%	13%	15%	9%	7%	8%	7%
